# Five Solar Cycles of Solar Corona Investigations

**DOI:** 10.1007/s11207-022-02007-3

**Published:** 2022-07-11

**Authors:** Ester Antonucci

**Affiliations:** Osservatorio Astrofisico di Torino, Istituto Nazionale di Astrofisica, Via Osservatorio 20, 10125 Pino Torinese, TO Italy

## Abstract

These are the memoirs of fifty years of research in solar physics, closely related to the history of three of the major solar space missions, from the Solar Maximum Mission, SMM, to Solar Orbiter, at present in navigation toward vantage points closer and closer to the Sun. My interest in solar physics was stimulated by the studies on cosmic rays at the University of Turin, and the research in this field initiated at Stanford University as a postdoctoral fellow in the team of John Wilcox with studies on the large-scale corona and its rotation. Thanks to Alan Gabriel, during my first space mission, SMM, I was involved in the operations and scientific data analysis of the Soft X-ray Polychromator. Together with Giancarlo Noci and Giuseppe Tondello, I participated in the realization of the UltraViolet Coronagraph Spectrometer, NASA/ASI, flown on-board SOHO. After this experience there was the opportunity to participate in the formulation of the proposal of the Solar Orbiter mission, and to guide the team, which for this mission developed the Metis coronagraph, up to the delivery of the instrument to the European Space Agency in 2017.

## Introduction

The Sun has been the object of my scientific interest for five full solar cycles, including the first years of research on galactic cosmic rays when I realized that the magnetism and the activity of the Sun were affecting the flux of these particles even at relatively high energies. The second half of the twentieth century was marked by extraordinary breakthroughs in our knowledge of the Sun, and the environment that the Sun creates, thanks to the access to space observations, and when I started my research activity in solar physics, in the 1970s, the solar emission at the short-wavelength wing of the electromagnetic spectrum was at last almost fully accessible. I had the opportunity to be involved in three of the major space missions dedicated to the study of the Sun: the Solar Maximum Mission (SMM) launched in February 1980, just seven years after the Apollo Telescope Mount (ATM) on Skylab (1973 – 1974), which was the first large observatory in space; the Solar and Heliospheric Observatory (SOHO), initially dedicated to the observation of the Sun at solar minimum, launched in December 1995 at the end of solar Cycle 22; and finally Solar Orbiter launched in February 2020, at the time of this writing on its way to reach perihelia closer and closer to the Sun. With regard to the last two missions, I was not only involved in the data analysis and interpretation but also in the development of two innovative instruments for the observation of the solar corona. The first of these was the UltraViolet Coronagraph Spectrometer (UVCS) on SOHO, a NASA coronagraph built with a significant contribution by part of the Italian Space Agency (Agenzia Spaziale Italiana, ASI). For the second instrument, ASI took the lead in providing the ultraviolet and visible-light coronal imager Metis for the Solar Orbiter mission. In my studies and professional life, my choices were mainly determined on the basis of scientific curiosity and interests that matured progressively, as well as by the influence of outstanding scientists I had the honor to meet and, in some cases, to work with.

## My Early Years

I was born in Boves, a town in the province of Cuneo (Piedmont), in the early morning of March 10, 1945 during the curfew of wartime. My first days of life were certainly not easy ones for my parents; bombings and conflicts were a daily reality. On the other hand, there was a widespread feeling that the end of the Second World War was imminent. It was indeed just a matter of weeks away, and Northern Italy would be liberated soon after, on April 25. I grew up in a loving and caring family. In the years immediately following the end of the war, still trying to overcome the moral and physical ruins of the war and to rebuild a country that had been heavily hit, people were aware that a hopefully brighter new era was beginning.

**Figure 1:**
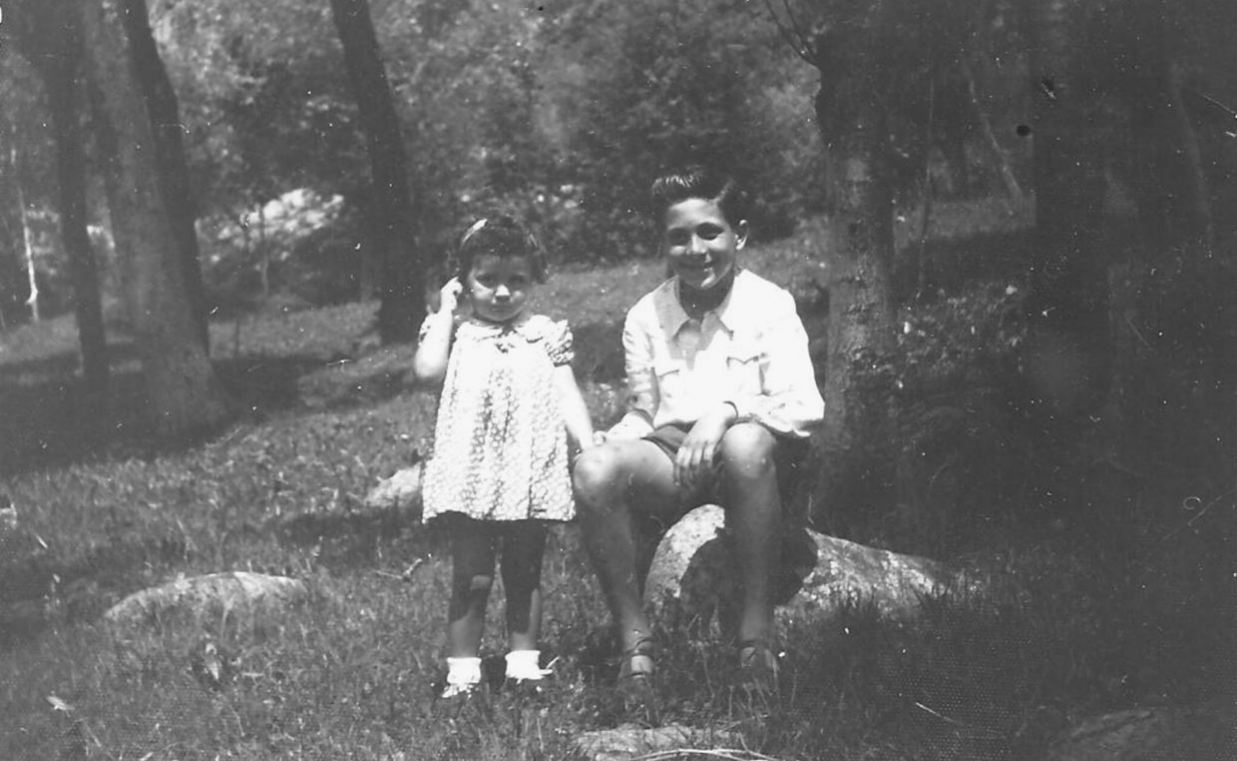
A summer day in Valdieri (Cuneo) with my brother Remo, 1947.

My father, Egidio, was born in Arpino, the ancient Roman town of Arpinum, birthplace of Marcus Tullius Cicero and Gaius Marius. When I was a child, people in Arpino were still tied to the glorious Roman past. The cyclopean walls of the acropolis, enclosing an almost unique ogive arch, however, testify to an important Pelasgian–Mycenean influence preceding the Roman era and place the origin of the town in more remote epochs. As a young man my father was serving in the Royal Guard in Rome when an army division, the Frontier Guard, was formed and toward the end of 1934 he was sent as part of this new army force to the French border in a small town of the Alps, Briga Marittima (formerly part of Italy, but ceded to France in 1947, and now named La Brigue). On Christmas Eve, just after he arrived at Briga, he met my mother, Elisabetta, who was back home after some years spent in Germany, the homeland of my grandmother who had married a young Italian man from a family in Briga. This is why my grandparents used to send their children to spend a few years with their relatives in South Germany. My parents married soon after they met and my brother, Remo, was born in Briga before they moved to Cuneo and then to Boves. During the last two years of the war, Boves was the site of many tragic events. My mother was a courageous woman who saved my father from being seized during a vindictive attack by the German army in retreat in the very last days of the war. She was perfectly at ease in her role of housewife and mother, although, given a chance, she would have liked to be a medical doctor. My father rejoined the army, which was reconstituted after wartime. He always encouraged me to study and follow my inclinations. When I was a child, summer holidays were often spent visiting Arpino and Sorrento, La Brigue, and Nice and biking and hiking in the valleys close to Boves during the weekends (Figure 1).

**Figure 2:**
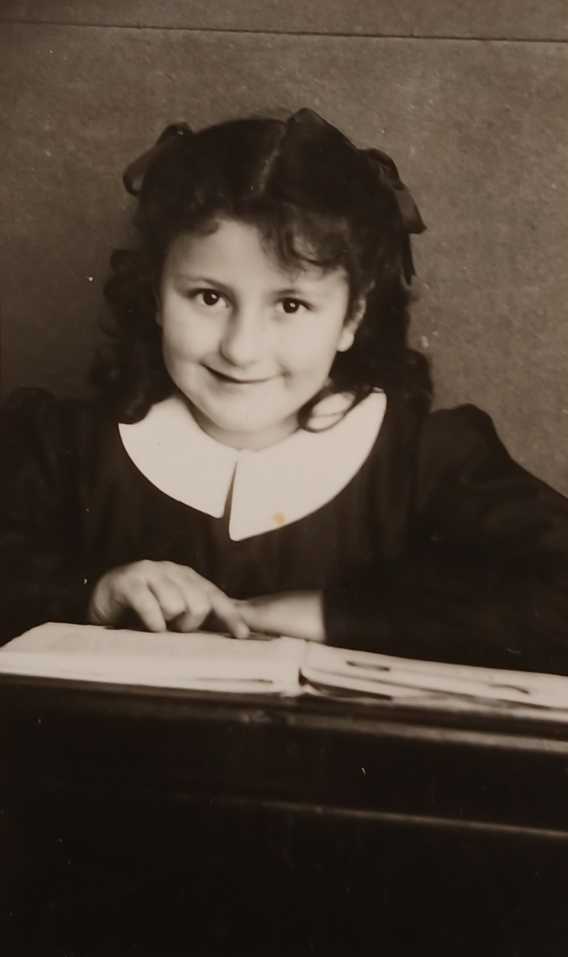
First year of elementary school in Boves, 1950 – 1951.

Of the times of the primary school in Boves in the 1950s (Figure 2), I remember long walks to go to school in cold and snowy winters. Fresh snow was always a lot of fun and as I walked to school, I had in front of me the rising sun and the Bisalta, a beautiful mighty mountain rising just at the edge of the inhabited area. I remember a class of little girls in black uniforms, older than me, since I started to attend school a year early, and the pleasure of learning from my severe but caring teacher, Dina Lanteri. One of my classmates was a war orphan, the symbol of the heavy price that Boves paid for the war; in consequence of the foolish and disastrous campaign in Russia alone eighty young *Alpini*, the army’s specialist mountain infantry, never came back home; they had been sent to die in the snow. I remained in touch with my teacher for the rest of her long life, she passed away, perfectly clear-minded, when she was 103 years old.

Arpino, with the same number of inhabitants as Boves, had a long cultural tradition and there the students could complete their education up through the Classical Lyceum.^1^ Boves instead was a rural town hosting only the primary school. Thus, when I was 10 years old, the only one of my classmates who was continuing her studies, I had to commute each day by train to Cuneo, 10 km away. In my long walks from the Cuneo train station to the school I sometimes dreamt about my future but my imagination did not go beyond the end of the century, which seemed so far away; sometimes I could see myself working in a vaguely defined scientific laboratory. In the days before the beginning of the school year, there was the ritual of carefully covering my school books, which I enjoyed as being the start of a new little cycle. Even now in the digital era, I much prefer to read the pages of books on paper that I can hold in my hands.

My family assured me a happy childhood and always supported and encouraged my choices, so that when I was 13 years old, as my brother did before me, I chose to attend the Scientific Lyceum named after Giuseppe Peano, the mathematician born in Cuneo who was one of the founders of mathematical logic. By that time, we were living in Cuneo, just two blocks from the Lyceum. I was fortunate once more in having the opportunity to profit from the lectures of excellent teachers, especially those teaching humanistic subjects.

My family has been always important to me and now that they are no longer with me, I fully realize how much I owe them: a positive attitude to life and friendship, the love for nature and the animal world, and the energy that I could spend in my personal and professional existence.

## University Years and Research on Cosmic Rays

During the last months of high school in 1963 when the time to enter the university was approaching it was quite difficult for me to decide the line of my future studies. Physics was not the only field I was interested in; I also loved architecture, philosophy, and mathematics. However, there were aspects of physics that were quite intriguing, such as the analogy of the planetary system around the Sun – the only one known at that time – and that of the electrons orbiting around the nucleus of an atom. When it was time to enroll at the University of Turin, the ultimate decision was to choose experimental physics. In the first years of university, I remember the perfect lectures held at the Mathematics Institute by Francesco Tricomi, well known for his studies on partial differential equations, as well as those of Gleb Wataghin, long-time director of the Physics Institute, who pioneered the research on cosmic rays in Turin. Later, the lectures of Carlo Castagnoli, who often referred to the most important experimental projects being carried on in particle physics, were a decisive factor in the choice of the subject of my thesis for the *Laurea*.^2^

### The Team of Carlo Castagnoli

Before taking over the chair in Turin in 1961, Castagnoli worked in Rome with Edoardo Amaldi, who was one of the ‘via Panisperna boys’ together with Enrico Fermi and Emilio Segrè, both Nobel laureates and both of whom emigrated to the USA when in the Fascist period the racial laws were enforced in Italy. Amaldi was a key person in establishing the Conseil Européen pour la Recherche Nucléaire (CERN) in Geneva in the years 1952 – 1954, the institution dedicated to the study of elementary particles. He was also a key person in starting the European space effort with the establishment of the European Space Research Organization – European Launcher Development Organization (ESRO/ELDO), precursors of the European Space Agency (ESA). In the wake of the Amaldi school, Castagnoli did orient the research of his newly formed team in Turin to particle physics, but with a focus on the particles that are accelerated in nature, that is, cosmic rays, a field of research already pursued in Turin. Moreover, he was convinced that cosmic rays should also be investigated as ‘astroparticles’ (a term at that time not yet in use) and encouraged studies in astrophysics and plasma physics. New stations were set up at Plateau Rosa on the Alps (elevation 3000 m) to detect air showers and in Turin under the Monte dei Cappuccini^3^ for the detection of the underground muon component. While the tunnel joining Italy and France was under construction, particle detectors were also positioned under the Mont Blanc. Some years later, the Mont Blanc station was set up to capture low-energy neutrinos. The first burst of neutrinos was detected on February 23, 1987, and the event was interpreted as probably associated with a supernova explosion (Aglietta et al., 1987).

Joining Castagnoli’s team in 1966, I was assigned to the tiny unit under the guidance of his wife, Giuliana Cini Castagnoli, scientific director for the Monte dei Cappuccini station, which became operative in that same year. She was involved in the set-up and operations of the station with the support of a young researcher, Maria Adele Dodero. The fruitful and friendly collaboration with Maria Adele lasted throughout my career. The object of my thesis research was to investigate the muon component of the galactic cosmic-ray flux at energies above 100 GeV. The $\mu $ mesons, secondary particles formed from the primary protons when they cross the Earth’s atmosphere, were detected with a set of ‘telescopes’ formed by solid and liquid scintillators positioned at a depth of 70-meter water equivalent (m.w.e.) under the Monte dei Cappuccini.

The focus was on searching for anisotropies in the flux of cosmic rays, either of a sidereal nature, which could enlighten us on the origin of such particles coming from sources outside the solar system, or due to modulations occurring in their propagation across the interplanetary environment. In those years interplanetary space was the object of great interest arising from the first exciting results of the space exploration initiated in 1959, which confirmed the existence in the heliosphere of the solar wind as predicted by Eugene Parker.^4^ According to the data acquired at the Cappuccini station, the existence of a sidereal anisotropy of the galactic cosmic rays at those energies remained uncertain, but we found cosmic-ray modulations of solar origin to be ascribed to the structure of the interplanetary medium and influenced by solar activity, which at that time was dominated by long-lasting active longitudes.^5^

In our laboratory I became accustomed to working with scintillators, photomultipliers, and the related electronics needed to build the telescopes for the detection of the $\mu $ mesons. However, data analysis was the research aspect I most enjoyed, especially because the data we were acquiring concerned an unexplored energy band. Programs and data were transformed in punched cards, then the heavy boxes containing the cards were delivered to the computer technician to be fed into a big IBM computer at the underground level of the Physics Institute (the first computer of the Institute, preceding the IBM one, was an Elea computer built by Olivetti in Ivrea). After a while, depending on how many others were before us, we picked up the results of our analysis. Most plots and graphs were, however, still drawn by hand and this gave us quite a lot of time to think about the data, and some of the effects could be indeed spotted in such a way before being confirmed by computer analyses.

The work with the Castagnoli team lasted about six years, from 1966 to 1972. Those years were extremely exciting for young students, since all universities in Europe were going through an extraordinary epoch of changes and novelties, debate and protest. The so-called ‘wave of 68’ in Turin begun as early as 1967 when the first student protests, assemblies, and occupations in some of the university departments had already started. Just two years later, in 1969, the prolonged protests and strikes of the metal-workers of the large FIAT factories in Turin heavily affected the whole city. In the end, the new statute of rights, granting more benefits to the industry workers, was signed. The Physics Institute was also involved in this new wave of endless discussions on politics, dealing with the need for justice and equality of rights all over the world, but, unlike other departments, studies and exams were not forgotten. Although understanding the students’ quest, I was quite disappointed when attending my first conference of the Italian Physics Society in Rome, where I was giving my very first talk in 1968, the participants at the meeting were met with protests by the students of Rome’s La Sapienza University.

In May 1969, the majority of the Castagnoli team attended the Ettore Majorana Summer School on space physics and astrophysics in Erice, Sicily. The school was exceptionally interesting for me, offering, for instance, the opportunity to attend the lectures of Bruno Rossi, a pioneer in space plasma research and in X-ray astronomy, who left Italy before the Second World War as a consequence of the racial laws, and of William A. Fowler, who a decade later won the Nobel Prize. Several new results in solar heliospheric physics, cosmic rays, and astrophysics were presented at this school, such as the recent detection of solar neutrinos and of the X-ray radiation from the Sun, and the measurements of the infrared cosmic background reported by Kandiah Shivanandan of the Naval Research Laboratory (NRL).

### Galactic Cosmic Rays Detected Underground

Great impetus was given to the search for cosmic rays with energy higher than those captured by the neutron monitors at ground level (that is, below 10 GeV) due to the stimulating atmosphere generated by the International Geophysical Year 1957 – 1958, the first large postwar international initiative by some of the scientists involved in geophysics and solar activity, held in correspondence to the peak of solar Cycle 19. These were also the years when the first Soviet and, four months later, the first American artificial satellites were launched into space (October 4, 1957 and February 1, 1958, respectively). The world was moving again and, this time, more peacefully.

Five underground stations were set up and became operative between 1958 and 1961 at depths between 30 and 60 m.w.e.. The Cappuccini station, set up in 1966, was the deepest one at 70 m.w.e.. We were accessing primary particles with energy above 100 GeV. At these energies, the cosmic-ray intensity modulation was thought to be of a sidereal origin. However, the results obtained in Turin showed that solar activity was also affecting the flux of these particles and in a different way from that for the lower-energy particles detected at ground level. In the years of maximum activity during solar Cycle 20, two rather well-defined long-lived active longitudes, almost 180° apart and with a strong north–south asymmetry, produced 80% of flares. The interpretation was that these active longitudes formed corotating streams due to the injection into the interplanetary space of magnetic inhomogeneities by individual flares, thus reducing the access of the high-energy galactic cosmic rays and inducing a long-lived modulation of the interplanetary medium observable in correspondence to their central meridian passage^6^ (Antonucci, Cini Castagnoli, and Dodero, 1971a). The recurrent decreases in particle flux were also associated with enhancements observed in the intensity of the interplanetary magnetic field measured with the instruments of Pioneer VIII, at the time at 26° East from Earth. The most interesting effect of the corotating structures screening the cosmic radiation was a twenty-seven-day periodicity not detected at lower energies (Antonucci, Cini Castagnoli, and Dodero, 1971b).

We also found an effect that nowadays would be considered relevant for space-weather forecasting. The high-energy cosmic-ray diurnal variation was strongly enhanced just a few hours after the occurrence of the flares giving rise to the Forbush decreases observed at much later times with the neutron monitors on the ground. This was interpreted as being ascribable to the larger particle gyroradius (above ≥10$^{7}\text{ km}$ for particles with energies ≥100 GeV) allowing a sampling of the interplanetary space conditions much closer to the Sun and of the magnetic cloud ejected by the flare when this reached dimensions comparable with the particle gyroradius itself (Cini Castagnoli and Dodero, 1969; Cini Castagnoli, Dodero, and Antonucci, 1970).

It was noted that the analysis of solar activity based only on flare occurrence was insufficient: ‘other phenomena not yet identifiable could contribute to the modulation’ (Antonucci, Cini Castagnoli, and Dodero, 1971c). Indeed, there was already an understanding of phenomena such as the association of flares with a cloud of plasma and magnetic inhomogeneities injected into the interplanetary space, the distortion of the magnetic field with the increase of the azimuthal component and the formation of a shock wave in the solar wind, the latter giving rise to the Forbush decreases observed in the lower-energy particle flux detected at ground level. However, coronal mass ejections, at first named coronal transients, had not yet been observed. Furthermore, it was not yet clear that the high-speed streams with a persistent character in certain phases of the cycle, which were ascribed either to Bartels’ unknown M regions or sometimes to recurrent flares, had their origin in coronal holes. Indeed, the first well-observed coronal transient was imaged during the Skylab era on June 10, 1973 and coronal holes were identified in rocket flights starting in 1970 and interpreted as the source of recurrent high-velocity solar-wind streams in 1973 (Krieger, Timothy, and Roelof, 1973).

## From Cosmic Rays to Solar Physics

### Solar Physics at Stanford University

The evidence of the Sun as regulator of the high-energy cosmic-ray flux and the exciting discoveries from space of the physical conditions of the interplanetary medium aroused my interest in solar and heliospheric physics. Once the cycle of the university studies was over, we were advised and encouraged to broaden our experience by spending some years as visitors in scientific institutes abroad. Thus, I got in touch with John M. Wilcox to explore the possibility of joining his group as a postdoctoral fellow. I was particularly interested in the results he obtained with Norman Ness on the interplanetary magnetic-field sector structure, evidence for persistent regular magnetic-field patterns in the heliosphere (Wilcox and Ness, 1965). John Wilcox welcomed my proposal and in November 1972, I left Turin to join his group at the Institute for Plasma Research at Stanford University, as a postdoctoral ESRO/ELDO fellow. This is how I began my studies in solar physics. John Wilcox had just moved from the University of California, Berkeley, to Stanford University with the intention to build a solar observatory, now named after him: the Wilcox Solar Observatory in the Stanford foothills.

**Figure 3:**
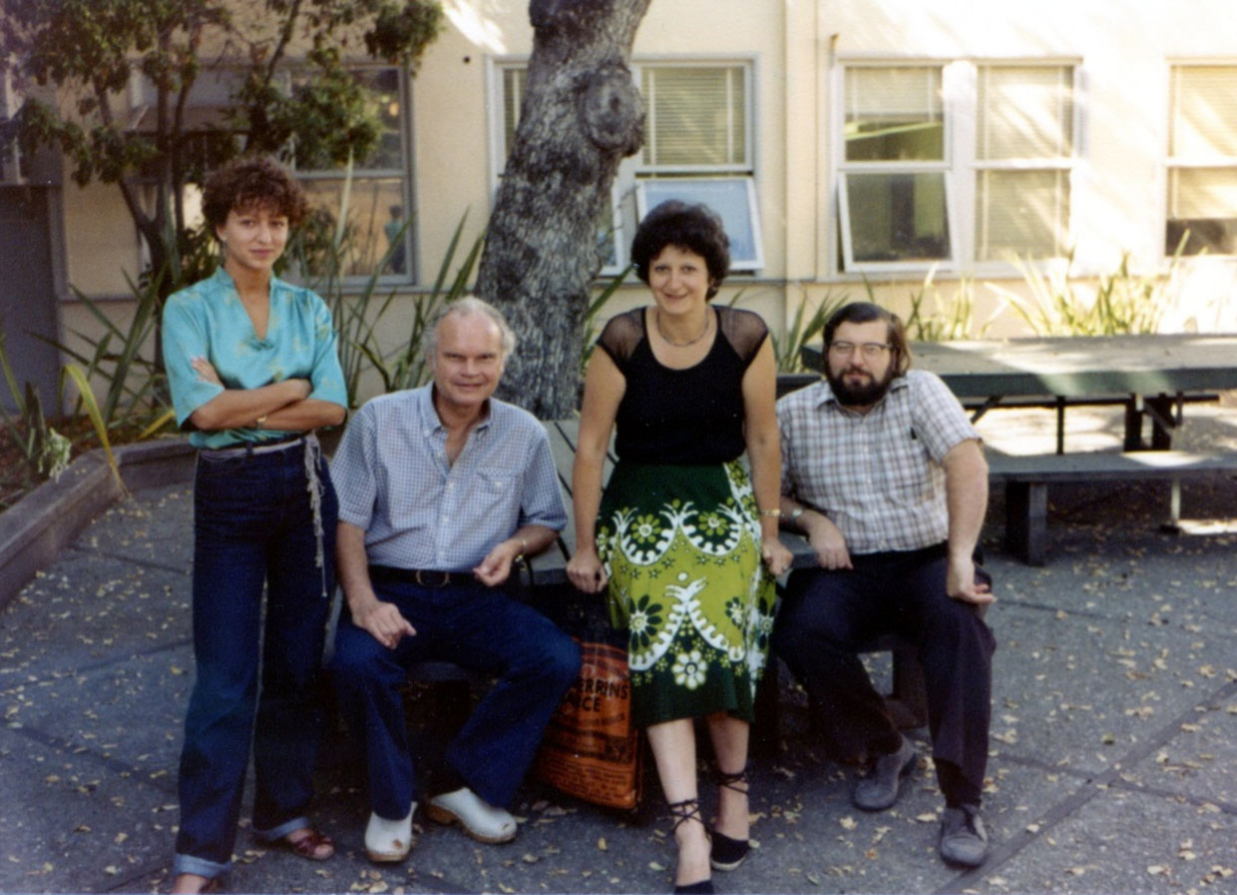
Stanford University, from the left Maria Grazia Borrini, John Wilcox, Ester Antonucci, Phil Scherrer, July 1980.

My first travel to the US was an adventure, I arrived two days later than scheduled because of an airline strike in Italy on the day of my departure and an airplane technical problem in the US on the way to San Francisco. Fortunately, upon arriving at the San Francisco airport John Wilcox rescued me and during my first days in Palo Alto I was a guest of his family. When I started my life on campus, I realized I was in one of the most important and challenging scientific environments and one of the most beautiful university sites. On the other hand, I also discovered to my great surprise that I was quite an exception, since there were no women in the Stanford physics departments. Indeed, graduate students, faculty, and research staff in the four large physics institutes – Plasma Physics, Applied Physics, Geophysics, and Radioastronomy^7^ – were all men. The minorities were represented by myself in the Plasma Physics department and a black graduate student in the Applied Physics department. Secretaries and librarians were almost exclusively of my gender, and they were delighted to support and help me in all possible ways. The magazine *Newsweek* contacted me for an interview, but I refused, not being sufficiently fluent in English to deal with such a delicate matter as minority issues. In the end, it turned out to be not difficult, although not immediate, to adjust to this situation, which was certainly unusual, not only for me but also for my colleagues. After my visit, Maria Grazia Borrini from Florence visited the Wilcox group for a while (Figure 3). A few years later, in 1978, the first American woman in space, Sally Ride, graduated in physics from Stanford University.

Life on campus was fantastic and certainly quite different from my previous life in an old university such as the University of Turin, dating back to the fifteenth century, whose departments were scattered all over the town center and not gathered in a campus as at Stanford. The Stanford International Center provided a great opportunity to meet new friends coming from everywhere and to attend interesting lectures and discussions, opening a new universe for me. A novelty was also the always positive attitude pervading almost everyone, compared with the thread of skepticism that sometimes can be sensed in people coming from the old European countries. There is no need to describe the atmosphere of the early 1970s in San Francisco and the universities. Not only the Stanford campus but also California was an enchanting state, with the lively and beautiful San Francisco, the ocean with its very cold waters and wild life, the deserts, and the forests so unexpected for a Mediterranean person. It was a state still preserving so many lands that were almost untouched by man, whilst in Europe every single square centimeter, except the mountains, bears the mark of centuries of history. It was a country much greener and with bluer skies than nowadays, when the almond and apricot trees were still shaping the landscape near Cupertino.

On my desk, on the first day of work at Stanford, I had all that was needed for my research: a paper notebook, a pen, and an absolutely necessary blue and red pencil to draw the polarity of the interplanetary magnetic field on 27-days Bartels charts. A new PDP/11 computer was located in front of the office I occupied in my first weeks, an office that then became the Wilcox office. This was a new way of interacting more easily with a computer; punched cards were no longer needed, but we still had to access the computer one at a time. Of that time only the beautiful old oak that was in front of my office survived and is still there. All the buildings have been replaced by new ones and the last time I visited the Stanford campus I had some difficulty in recognizing where ‘my’ mighty oak was.

In addition to Leif Svalgaard, visiting from Denmark, and myself, the Wilcox group included three graduate students: Phil Scherrer, involved in the project of the new solar observatory, Tom Duvall, and Phil Dittmer. Twice a day during coffee breaks in the Wilcox office we exchanged opinions, ideas and the results we were achieving. One day a telex informing the scientific institutions about the presence of a huge, unexpected perturbation with its origin in the Sun, propagating in space and potentially hitting the Earth, monopolized our attention. This was 1973, when Skylab was observing unambiguously the first coronal transients (MacQueen et al., 1974). We also attended a weekly science meeting in the office of Peter Sturrock, with Vahé Petrosian and their students, discussing the more recent solar-physics papers and conference contributions.

Soon after my arrival, I attended the fall meeting of the American Geophysical Union (AGU) in San Francisco. The first day of the meeting, a beautiful annular rainbow greeted us on our way to San Francisco along the highway on the hills. I have never again seen such a phenomenon. This was the first of a few great conferences and meetings I attended from 1972 to 1974, with the presence of astronauts just coming back from their journey to the Moon or from Skylab. Every result presented at these conferences was entirely new and exciting. At that time the largest meetings, such as those of the AGU, were attended by a few hundred participants and it was much easier to follow presentations even outside our own field of research, a way of broadening our views.

The results achieved by John Wilcox in the analysis of the Interplanetary Monitor Platform (IMP), Mariner and Pioneer data, obtained in the years 1963 – 1966, were paving the way for further studies on how the solar wind was organized within the sector structure in terms of magnetic-field direction and magnitude, plasma velocity, and proton density. This pattern, with alternating sectors of positive and negative magnetic-field polarity lasting several days, was found to corotate with the Sun and its influence on geomagnetic indexes and the low-energy cosmic-ray flux detected by neutron monitors was clear (see review by Wilcox, 1968). The other puzzling question, in order to unambiguously identify the interplanetary sector structure source, was how this persistent wind pattern relates to the large-scale photospheric magnetic field that was observed with the solar magnetograph at the Mount Wilson Observatory in the period 1959 – 1967. A quite intriguing finding was that the solar sector structure had little or no differential rotation in the low-latitude zones, that is, in the range 20° N – 20° S (Wilcox et al., 1970). Following a new approach – that is, studying the magnetic field of the Sun seen as a star with the Crimean tower telescope – Severny et al. (1970) established that the observed mean solar-field behavior allowed a prediction of the interplanetary field measured at Earth four or five days later. This was the cultural frame that allowed me to begin my research in solar physics.

### The Fe xiv Green-Line Corona and Solar Rotation

John Wilcox was expecting to obtain the first data of the new solar observatory under construction at Stanford during the time of my visit. This would have been my first opportunity to observe the Sun from the ground. Such an opportunity occurred once more in 1975 when Otto Kiepenheuer invited me to observe during the summer time at the Anacapri Observatory of the Fraunhofer Institute,^8^ but his sudden death just before the summer of that year, a great loss for the European solar-physics community, ended the chances of an experience as a ground-based observer.

There were delays in the completion of the observatory at Stanford and the first low-resolution maps of the Sun’s magnetic field date back to May 1976. Thus, even if a long set of the interplanetary magnetic-field polarity data – inferred by Leif Svalgaard (1972) from the perturbations of the geomagnetic field near the pole registered at Godhavn since 1926 – was available, there were no new solar data to play with. Thus, whilst each day I was learning a lot in that lively scientific environment, I realized I had to find some solar data to work with. Considering that no study on how the solar corona was organized in relation to the interplanetary sector structure had yet been made, I sent a letter to J. Sýkora, who I did not have the pleasure of knowing personally, asking for the data of the intensity of the Fe xiv green corona emission that he had compiled, transforming all green-line observations available from 1947 to 1968 into a homogeneous data set (Sýkora, 1971).

In the meantime, an intriguing peculiarity was noticed: during solar minimum the polarity of the inferred interplanetary magnetic field was found to be predominantly positive (away from the Sun); in other words, the sector structure was disappearing for a few solar rotations (references relative to the present understanding of this effect are reported in the review by Antonucci et al., 2020a). This magnetic configuration, associated with the position of the Earth along its orbit, could explain the anomalous cosmic-ray anisotropy observed with neutron monitors during the summer of 1954, by considering its dependence on the northern branch of the cosmic-ray intensity gradient across the equatorial plane (Antonucci, 1974a).

The green-line data, kindly provided by Sýkora, sounded coronal heights from 40 to 60 arcsec from the limb and were covering almost two full solar cycles. Thus, I presumed they were suitable for studying possible regular patterns of the corona within the magnetic sector structure. Indeed, we found that sector boundaries separating leading and following polarities of the interplanetary field were preferentially related to green corona enhancements, implying that enhanced coronal features present in each hemisphere were displaced by 90° or 180° in helio-longitude relative to the opposite hemisphere, depending on the presence of a four- or two-sector structure in the solar wind, respectively. In addition, the pattern reversed from one solar cycle to the next, according to the Hale law on the emergence of sunspot polarities (Antonucci, 1974b; Antonucci and Duvall, 1974; Antonucci and Svalgaard, 1974a). The present understanding of the boundaries later named Hale boundaries is reported in Antonucci et al. (2020a).

The set of green-line data was also suitable for a detailed investigation of the rotation of the corona through the solar cycle. It was not possible to apply to limb observations the typical method of tracing the motion of a solar feature due to solar rotation, thus I performed this study by applying autocorrelation techniques to the temporal series of the coronal data. The results were quite interesting: during the years before sunspot minimum, the degree of differential rotation of the corona was decreasing to approach rigid-rotation conditions. Moreover, differential and rigid rotation coexisted. Shorter-lived coronal emission was found to show the same differential rotation as the short-lived photospheric magnetic fields, whilst long-lived coronal enhancements were rotating nearly rigidly (Antonucci and Svalgaard, 1974b), as did the coronal holes observed with Skylab in the time frame 1973 – 1974 (Wagner, 1975). Active longitudes and rotational characteristics of the corona were also discussed by Sýkora (1971) on the basis of the same data set. The green corona emission, observed in the Skylab period, but not yet included in these first studies, was analyzed with Maria Adele Dodero when I returned to Turin, confirming that in the declining phase of the solar cycle coronal structures persisting more than one synodic period are rigidly rotating (Antonucci and Dodero, 1977; Antonucci and Dodero, 1979).

Intrigued by the coffee-break discussions about the new observational results on the coronal configuration, mentioned in the paper by Svalgaard, Wilcox, and Duvall (1974), I attempted to describe the evolution of the coronal pattern through the solar cycle as due to the rotation of the solar magnetic dipole. This hypothesis was phenomenologically consistent in my opinion with the general observational characteristics of the photospheric magnetic field and of the coronal patterns during the twenty-two-year magnetic cycle. Unfortunately, the referee thought that this was an awkward idea and the paper entitled ‘Solar Rotating Magnetic Dipole?’ was rejected (Antonucci, 1974c).

### Back in Turin in the Seventies

During the second year of the ESRO fellowship in 1974, I had to make a difficult choice. I was feeling completely ‘at home’ in Palo Alto and John Wilcox offered me the opportunity to continue to work in his group at Stanford also in the future. However, even if still today I consider California my second home, I chose to return to Italy since there was the chance to upgrade my permanent position at the University of Turin.^9^ A further extension of my visit in California would have paved the way to remain there forever. Back in Turin, I was teaching Laboratory Physics at the Institute of Physics and then Fundamental Physics at the Institute of Agriculture. These years were interesting but at the same time troublesome because of the new wave of protests that spread beyond the universities. This time the protests were more violent. In 1978, during the worst phase of terrorism, the former prime minister Aldo Moro was murdered, shot in Rome by members of the Red Brigades. When I was back in the US in 1980, reading the Italian newspapers I realized that some of the students who attended my lectures (I should say, disturbed my lectures) at the Institute of Agriculture later became leaders of one of the worst terrorist nuclei called ‘Prima Linea’ (Front Line). Fortunately, they were a minority and although very aggressive, nothing happened to me, even though at the Institute of Agriculture I was one of the few professors who never stopped giving their lectures. I was trying to make the point that the paralysis of the university was detrimental first of all for those students who could have a better chance to progress in their life only by pursuing their studies.

#### Chromospheric Rotation with the Ca ii K_3_ Filtergrams Detected at the Anacapri Observatory

Carlo Castagnoli was opening new lines of research, such as geophysics, and the Monte dei Cappuccini cosmic-ray station was no longer one of his priorities. In the years 1974 – 1975 the station became no longer operative. Thus, it was time to find a way to continue solar-physics research that nobody else was pursuing in Turin and to look for financial resources to support my own research. First, I needed data to continue for a while both solar and cosmic-ray research. Solar rotation was still an interesting topic to be explored by studying other layers of the solar atmosphere. For instance, chromospheric rotation at that time was inferred by tracing the motion of individual features. Hence, it would have been interesting to apply techniques of analysis analogous to those used for the coronal data to determine the rotation as a function of lifetime and size. The daily observations performed at the Anacapri Observatory were certainly suitable for this goal. Thanks to Kurt Sitte, often a visiting professor in Turin, I was introduced to the director of the Fraunhofer Institute, Karl Otto Kiepenheuer, and I was invited to visit Freiburg in order to present to him my project to analyze the Anacapri data. The digitization of the images of the Ca ii K_3_ filtergrams registered daily would have allowed a frequency analysis of the chromospheric emission. Our first conversation took place in 1974 in Kiepenheuer’s office, where I was invited to sit at the desk that in the past had belonged to Joseph von Fraunhofer.

**Figure 4:**
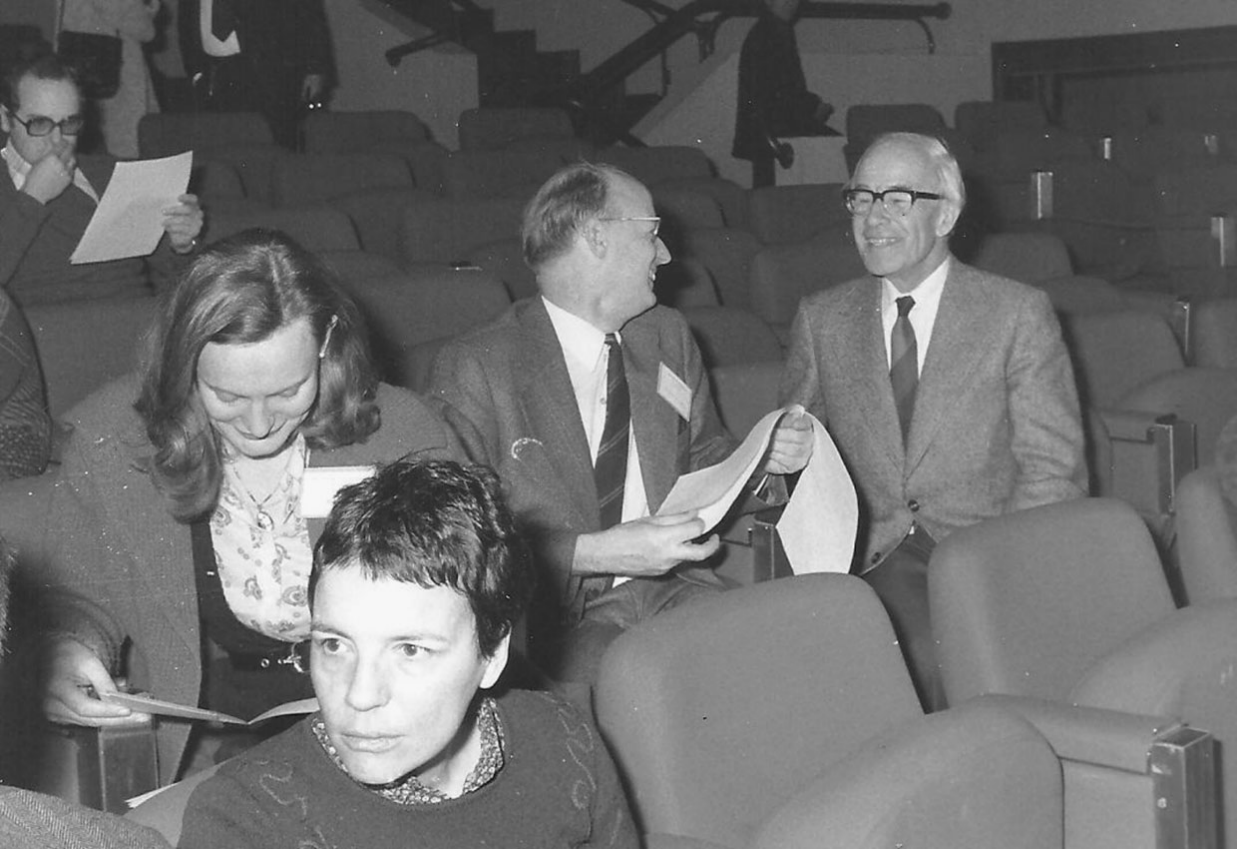
First European Solar Physics Meeting, Florence, February 1975. On the right is Otto Kiepenheuer.

Otto Kiepenheuer was a charismatic person indeed (Figure 4). Only years later, I became fully aware of the role that he played in safeguarding solar observatories and protecting solar physicists all over the European countries occupied by the German troops during the Second World War, as well as in developing solar physics in the postwar years. It is interesting to note that in wartime he pursued the innovative idea of observing the Sun from a vantage point outside the atmosphere in order to explore the solar ultraviolet radiation. This would have been possible in collaboration with Werner von Braun, who was developing the first powerful rockets capable of reaching altitudes well above the ozone layer absorbing the short-wavelength ultraviolet radiation emitted below 2900 Å. At the same time, he had the clear view that the corona was the key to the understanding of all solar–terrestrial relationships, which were the object of intense study in the Luftwaffe research centers, for military reasons of course. In Europe, space-weather studies, strongly supported by the Luftwaffe leaders, had started around 1939 under the coordination of Hans Plendl, who was setting up an efficient network of ionospheric stations to predict solar disturbances in radio transmission during the raids of the air force. Historical studies on the role played by Otto Kiepenheuer in the war years have been carried out by Michael P. Seiler (‘Solar research in the third Reich’, 2006). Unfortunately, Kiepenheuer did not see any of the results obtained on chromospheric rotation using the Anacapri data, as he suddenly died in 1975.

Digitization of the astronomical images was not easily performed in the early 1970s. Fortunately, the scientists of the Institute for Elaboration of Information (IEI) of Italy’s National Research Council (CNR) in Pisa, contacted thanks to Roberto Falciani of the Arcetri Observatory, became quite interested in the project of digitizing the filtergrams provided by the Freiburg Institute. Two years of daily chromospheric filtergrams, 1972 – 1973, were processed using a flying-spot photometer controlled by a PDP/8/I computer. It was quite complex work in those years. The 1024 × 1024 optical density arrays were recorded on large magnetic tapes stored in the computer room. When this laborious work was over, during the restoration of the Renaissance building that hosted the CNR’s Institute on its ground floor, the roof of the computer room partially collapsed, damaging most of the tapes, which were no longer recoverable. This is why in the first analysis of the data completed in 1976 the full two-year temporal sequences of data were used, whilst in the following papers the study was limited to the period May 8 – August 14, 1972.

The first analysis based on the power spectra of the temporal sequences of the full set of digitized data, as expected, showed that during the years before sunspot minimum the chromospheric features with a lifetime of the order of or exceeding one solar rotation were also almost unaffected by differential rotation (Antonucci et al., 1977). These results were consistent with the rotation behavior of the green corona and the Skylab coronal holes. In the following two papers, the shorter data set was then used to study short-lived and small-scale/middle-scale chromospheric features, tracing their average daily displacement by computing average crosscorrelations of brightness features (Antonucci et al., 1979a,b). These features were found to rotate differentially, as do the Ca faculae and bright mottles.

Eventually in 1985, after almost one solar cycle of observations obtained at the Wilcox Solar Observatory at Stanford, it was time to propose to Phil Scherrer and Todd Hoeksema a study of the rotation of the large-scale persistent patterns of the photospheric magnetic field to corroborate the chromospheric and coronal results. It was a pleasure to work again, although for a quite limited period, with the Stanford group, which later became fully involved in the detection of the solar magnetic field from space. During solar Cycle 21 in the interval from 1976 to 1986, the photospheric magnetic-field pattern persisting more than one solar rotation showed, as expected, broad latitude zones rotating rigidly. Furthermore, a strong north–south asymmetry was found in the properties of rotation, which was more rapid in the northern hemisphere than in the southern one. These results suggested an association of the northern structure with the four-sector structure and the southern field, likely contributing to a two-sector structure in the interplanetary magnetic field (Antonucci, Hoeksema, and Scherrer, 1990).

#### Modulations of Cosmic Rays Detected on the Ground

On my return to Turin the interest in cosmic rays was still alive, but now the focus was on the modulation of cosmic rays of lower energy, below or equal to 10 GeV, detected on the ground since these data allowed studies over one full solar magnetic cycle, from 1954 to 1973.^10^ The results were quite interesting. The annual and semiannual modulation of the cosmic-ray intensity, due to the existence of perpendicular gradients across the heliospheric equatorial plane, showed an unexpected phase reversal at the reversal of the solar and interplanetary magnetic dipoles at the solar-activity maximum (Antonucci, Marocchi, and Perona, 1978a^11^ and references therein). This twenty-two-year cycle in the modulation of cosmic rays pointed out the significance of particle drifts due to gradient and curvature effects in the spiral interplanetary magnetic field, which are influenced by the field polarity (Perona and Antonucci, 1976). In conclusion, at the Earth’s orbit the contribution of transverse diffusion to the cosmic-ray transport in interplanetary space was found to be not negligible with respect to the convection of the solar wind and to the radial diffusion due to magnetic inhomogeneities. Additional contributions to the yearly modulations could be ascribed to perpendicular gradients induced by a hemispheric asymmetry existing in the sunspot-area data collected at the Astrophysical Observatory of Catania from 1954 to 1976 (Antonucci, Marocchi, and Perona, 1978b).^12^ However, our colleagues in the Bologna cosmic-ray group were not so convinced about the validity of these results. Thus, some years later, in order to settle this question, we decided to work together on a somewhat more extended set of data, from 1953 to 1979, employing a different analysis technique. This collaboration eventually confirmed the scenario deduced from the previous studies. In addition, a residual yearly variation with maximum at the time corresponding to the local galactic magnetic-field direction was present and proposed to be of sidereal origin (Antonucci et al., 1985a).

A breakthrough in the understanding of solar rotation and cosmic-ray modulation occurred a few years later when helioseismology discoveries made it possible to measure the rotation of the interior of the Sun and when, in its long journey in space, the Ulysses spacecraft, launched in 1990, explored the out-of-ecliptic heliosphere. These two achievements opened new scenarios in both fields. With regard to helioseismology, 1974 was a crucial year. At the Anacapri Observatory, Franz Deubner obtained a set of photospheric observations that enabled him to resolve a few stable eigenmode ridges of the 5-minute oscillations in the famous frequency versus horizontal wavenumber diagram. His findings were consistent with the predicted existence of fundamental modes of standing acoustic waves at the subphotospheric level (Deubner, 1975).

#### The Erice Summer School in 1976

The third Ettore Majorana Summer School I attended in Erice, organized by Bruno Caccin in August 1976, was of crucial importance to me. Some of the lectures were dedicated to the ground-based observations of solar oscillations and the models put forward to interpret them. With regard to the solar atmosphere, Alan H. Gabriel (1976) presented his new magnetic model of the solar transition-region derived on the basis of space observations. The encounter with Alan Gabriel started a new chapter in my scientific interests and a long-lasting collaboration and friendship. A short time after having attended this summer school, I received an invitation to apply for a position at the Appleton Laboratory in Culham, Abingdon, UK, in view of the launch of NASA’s Solar Maximum Mission (SMM). SMM hosted the Soft X-Ray Polychromator (XRP), an instrument developed jointly by the Appleton Laboratory, the Lockheed Palo Alto Research Laboratory and the Mullard Space Science Laboratory. The XRP team was guided by three Principal Investigators, one for each of these institutions: Alan H. Gabriel, Loren W. Acton, and J. Leonard Culhane, respectively. Since I was not interested in a permanent position at the Appleton Laboratory, in agreement with Alan Gabriel I applied for an ESA research fellowship, which allowed me to participate in the XRP experiment and in this way to gain access to the interesting field of the X-ray spectroscopy of flaring plasmas, a field totally new to me. It was a fantastic opportunity and quite a challenge to be involved for the first time in a space mission.

**Figure 5:**
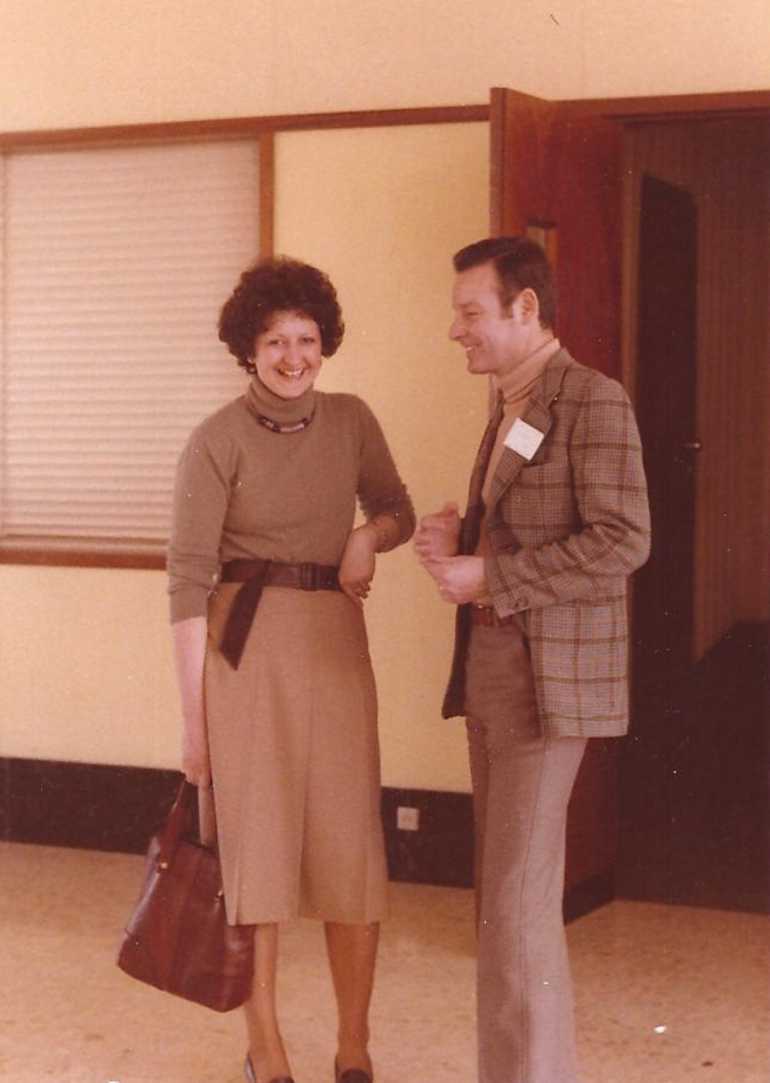
Second European Solar Physics Meeting in Toulouse, March 1978. With Giancarlo Noci and a heavy handbag (picture taken by Alan Gabriel).

**Figure 6:**
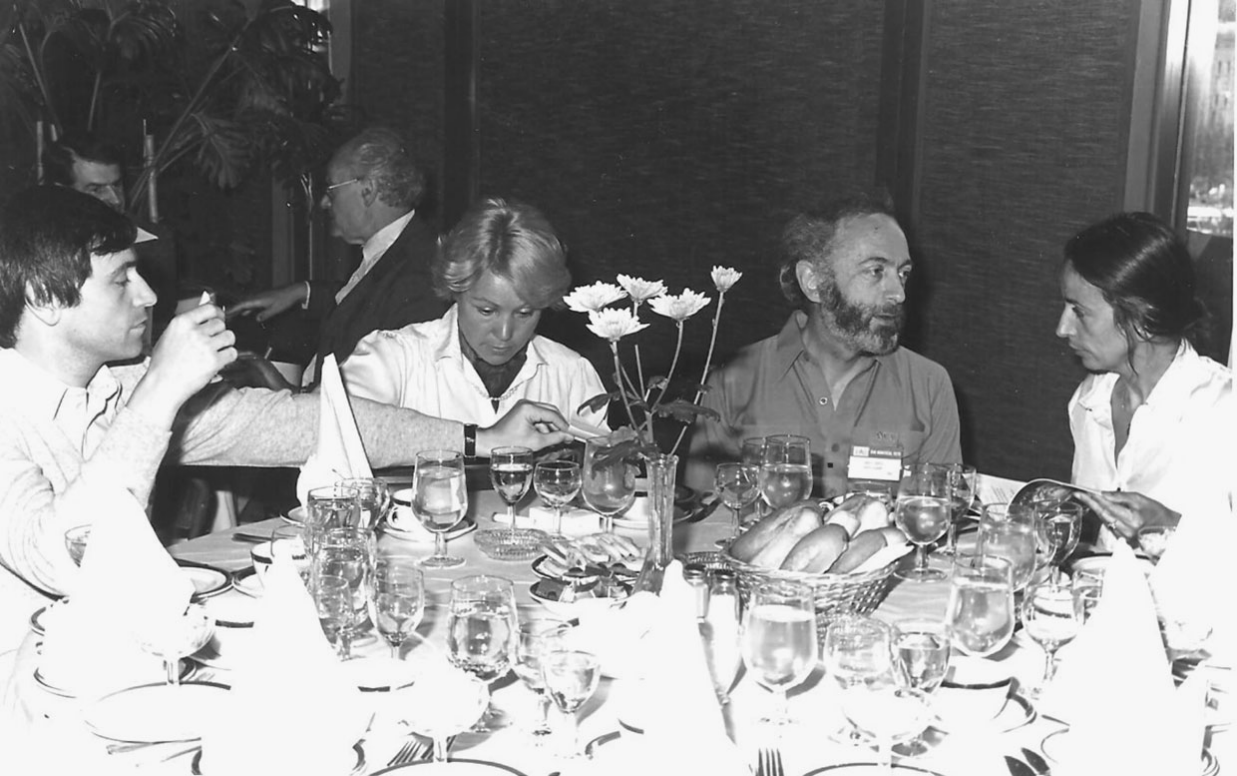
International Astronomical Union (IAU), XVII General Assembly, Montreal, August 1979. From the left: Serge Volonté, Michelle Loulergue, Alan Gabriel, and Françoise Bely-Dubau, belonging to the atomic-physics team supporting XRP-SMM (picture taken by Ester Antonucci).

In March 1978 during the second European Solar Physics Meeting in Toulouse (Figure 5) I discussed with Alan the research proposal I had to submit to NASA in view of my participation in SMM and, on this occasion, I met some of his collaborators who were deeply involved in the development of updated atomic-physics calculations for XRP (Figure 6). During a few visits to the UK in 1978 and 1979 I familiarized myself with flare data obtained during the Skylab mission, trying to interpret the oscillations identified in Extreme UltraViolet (EUV) lines during a loop brightening by Bruce E. Patchett (Antonucci, Gabriel, and Patchett, 1984). It took a long time to prepare and submit the paper, since in the meantime many exciting results were achieved thanks to the first XRP observations that completely absorbed our time. There were also the numerous documents relative to SMM and XRP to study, acronyms to memorize and so on, in order to be prepared to observe with XRP. In these years the Culham scientific area near Abingdon was chosen as the site of the Joint European Torus, the European project devoted to experiments of nuclear fusion that began in the early 1980s. Hence, in 1979 the Appleton Laboratory was incorporated in the huge area of the Rutherford Laboratory at Chilton, thereafter named Rutherford Appleton Laboratory (RAL).

## My First Space Mission – SMM

### The Solar Maximum Mission

**Figure 7:**
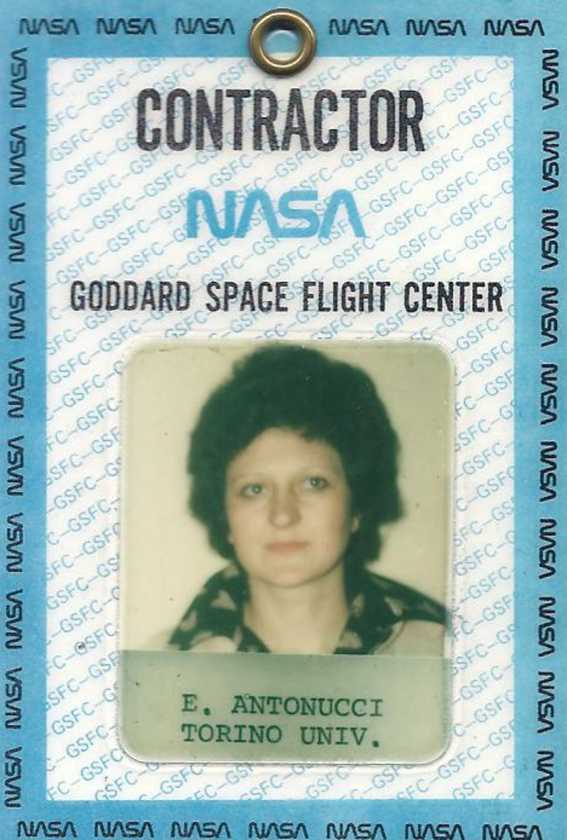
Pass to access the Goddard Space Flight Center, 1980.

The year 1980 was a milestone in my scientific career. In January I moved to Abingdon to collaborate with Alan Gabriel, thanks to the ESA fellowship that was granted to me. As the SMM launch was imminent, at the end of the month I moved to Greenbelt, Maryland, USA, to join as Co-Investigator the XRP team at the Experiment Operation Facility (EOF) of the Goddard Space Flight Center (GSFC-NASA) (Figure 7). I arrived just two weeks before the SMM launch. My arrival was probably wisely delayed until that moment so that there was not much time to realize how hectic was the activity of the XRP team at the EOF in preparation of the initiation of the mission and to realize that most of our offices in building 7, where we were spending very long days, were depressingly illuminated only by artificial light. Moreover, all the team members were much more experienced than myself. In the first days, a quick adjustment to the mixture of the various British and American accents and to the continuous use of acronyms was also necessary.

On February 14, whilst part of the team was at the Kennedy Space Center to be present at the launch of the spacecraft, most of us were watching the event at GSFC and when just after the launch the screen turned black for a second, I thought that the visit at NASA was ending in that moment. Instead, SMM was successfully launched and entered its nearly circular orbit around the Earth at an altitude of about 574 km with an inclination of 28.6° (Bohlin et al., 1980).^13^ This first successful step of the mission was celebrated the same day at Loren Acton’s house with all the XRP team members, since everybody immediately came back from the launch site.

Only six years had elapsed since the completion of the Apollo Telescope Mount (ATM) solar program on Skylab,^14^ the first observatory in space, when the SMM, the second large solar space observatory, was launched. The comparison with the many years needed nowadays to develop a new mission of the same complexity is amazing, also considering the quite limited means available in the 1970s and early 1980s in terms of computer capabilities and rapidity of communications.^15^

During the first SMM year I had the chance to meet, collaborate with and enjoy the company of new colleagues of the experiment teams, short-term visitors, and scientists supporting SMM operations coming from all over the world. Space solar physics has become since that time a truly global experience, a positive early example of involvement of scientists of all countries in pursuing the same research objectives. Some of the outstanding people I met during SMM times became lifelong friends.

SMM was the first solar satellite to be fully devoted to a specific scientific problem: the investigation of solar flares. Coordinated observations with five complementary instruments covering the energetic part of the electromagnetic emission from UV to gamma rays up to 160 MeV, and the visible-light coronagraph for the study of the ejecta during solar activity, were envisioned to address the ensemble of the complex phenomena occurring during such events. In order to obtain spatially coordinated flare monitoring, the instruments and module were designed to ensure an accurate and stable pointing at a selected active region and a good coalignment of the instruments with small fields of view. Imaging in hard X-rays with energies up to 30 keV and spectroscopically resolved soft-X-ray emission down to 1.7 Å could be achieved. With the support of optical and radio ground-based observatories the simultaneous coverage of the entire electromagnetic spectrum was ensured. An additional experiment, monitoring the total solar irradiance, turned out to be crucial to show beyond any doubt the solar-cycle dependence of this quantity. SMM had an important peculiarity: it was the first spacecraft designed to be retrieved by the Space Shuttle, which was going to be launched one year later. The many fundamental SMM results are summarized in two books: *Energetic Phenomena on the Sun*, The Solar Maximum Mission Flare Workshop Proceedings (eds. M. Kundu and B. Woodgate, NASA Conference Publication 2439, 1986) and *The Many Faces of the Sun* (eds. K., Strong et al., Springer, 1999).

#### Operations at the Experiment Operation Facility

The SMM observation program, highly structured in order to reach its goals, set an example for future solar space missions. The program was operated on a twenty-four-hour cycle and a detailed day-by-day planning of observations was carried out starting on February 17, 1980. I had the privilege of representing XRP at the first two SMM daily planning meetings, supported by experienced XRP people who were sitting not far from the planning table. XRP scientific operations started on February 19, as soon as the spacecraft was properly oriented. The analysis of the acquired scientific data was performed in as near as possible to a real-time manner to provide valuable feedback for the daily planning cycle. Evaluation of the scientific content and quality of the data was achieved within eight to twenty-four hours of its collection. The indepth analysis was also immediately initiated at the EOF, taking advantage of coordinated efforts with the other SMM experiments observing flare emission in different and complementary spectral ranges. The roles of the XRP planner and evaluator were performed on a rotational basis.

**Figure 8:**
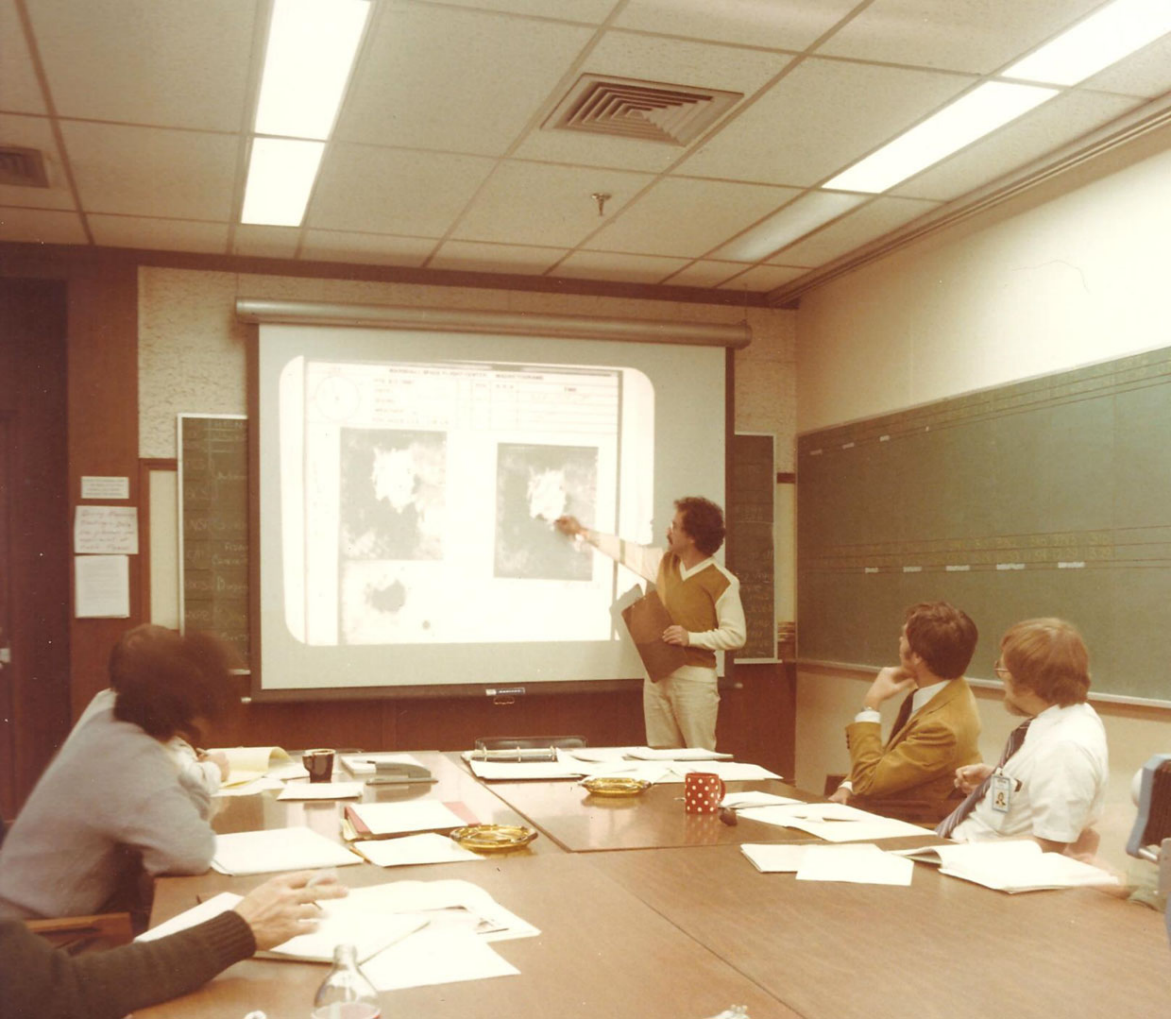
Experiment Operation Facility at Goddard Space Flight Center. Daily Solar Weather Report by David Speich during SMM operations,1980. At the right: W.J. Wagner and Bill Henze.

**Figure 9:**
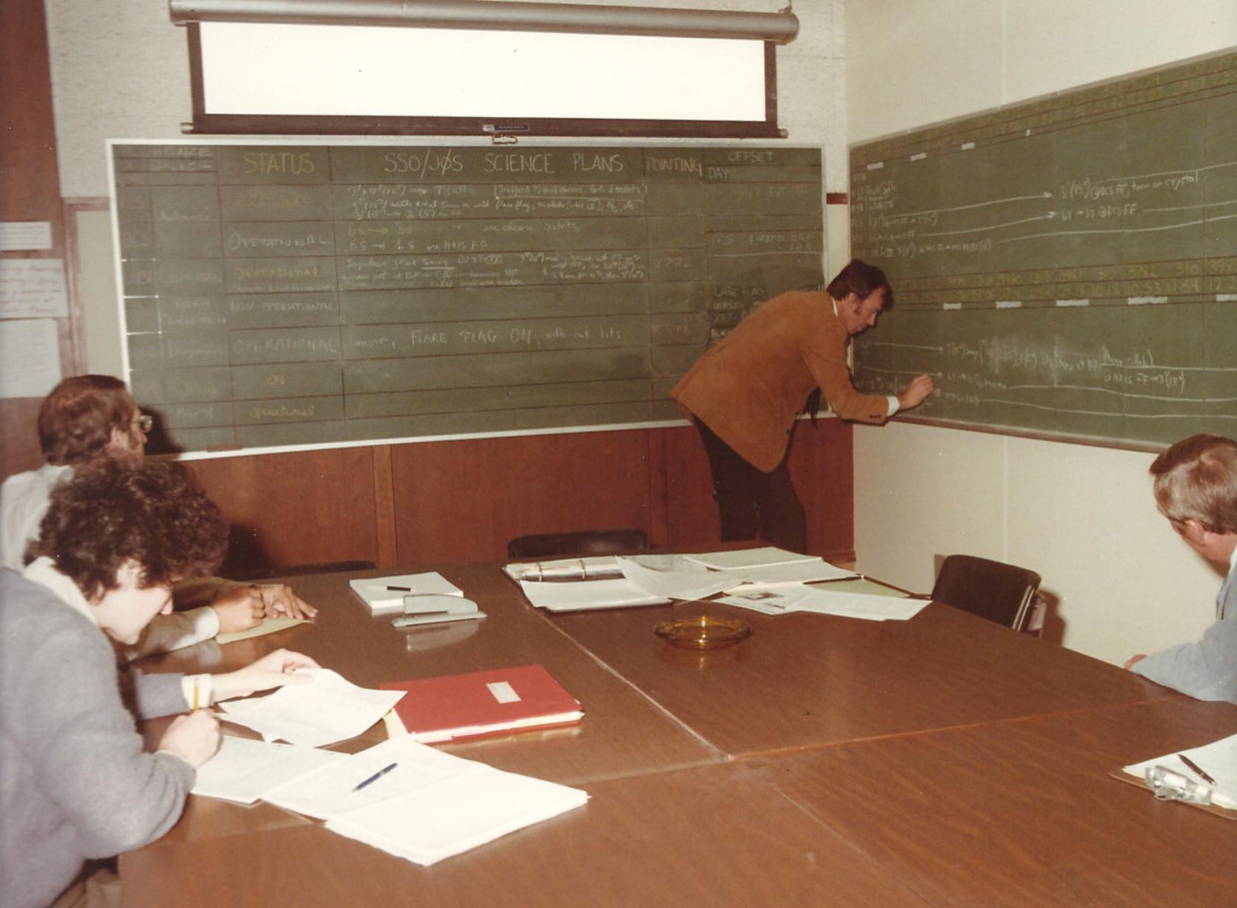
Experiment Operation Facility at Goddard Space Flight Center. SMM daily planning meeting during SMM operations, 1980. Bill Henze at the blackboard filling the instrument timelines. At the left: Ester Antonucci and Dave Rust.

During the daily planning at the SMM level, the most difficult decision concerned the active region to be selected for the pointing of the spacecraft.^16^ The choice of the active region was based on the daily forecast provided by the National Oceanographic and Atmospheric Administration (NOAA) (Figure 8).^17^ A large network of observatories, also part of the Solar Maximum Year effort, was involved in support of the NOAA forecast as well as in the coordinated observations program. Immediately following the daily solar-weather report, an orbit-by-orbit plan with the precise timeline of observing modes was formulated for the following day at the instrument level and presented by the instrument planner at the SMM planning meeting (Figure 9). The plan was formulated according to the identified Specific Scientific Objectives (SSO) addressed by ad hoc Joint Observing Sequences (JOS). During that SMM daily meeting the pointing decision sometimes turned out to be quite a challenge and gave rise to lively discussions. Each day we attended several meetings at the SMM and XRP levels before the scientific plans could be converted to command loads to be uplinked to the spacecraft overnight. Thus, the meaning of the mission acronym slowly evolved to become ‘So Many Meetings’. Punctuality and efficiency were a must and meetings were short and exhaustive, qualities that were an absolute prerogative of the time.

Although we were at the peak of Cycle 21, the first days were not very successful in our search of flaring regions. At last, on February 26, SMM pointed at the right active region and at 3:20 UT the first flare was observed. However, no major flare was observed until March 29. Then on April 7, the first class-M flare (M8) was detected and on May 21 the first class-X flare (X1).^18^ Finally, the first white-light flare detection occurred on July 1, 1980. With time, flares of increasing importance were observed and the challenge became to prepare and improve codes in order to quickly interpret the data, try to understand the observations, and present as soon as possible the preliminary results to the solar community.

**Figure 10:**
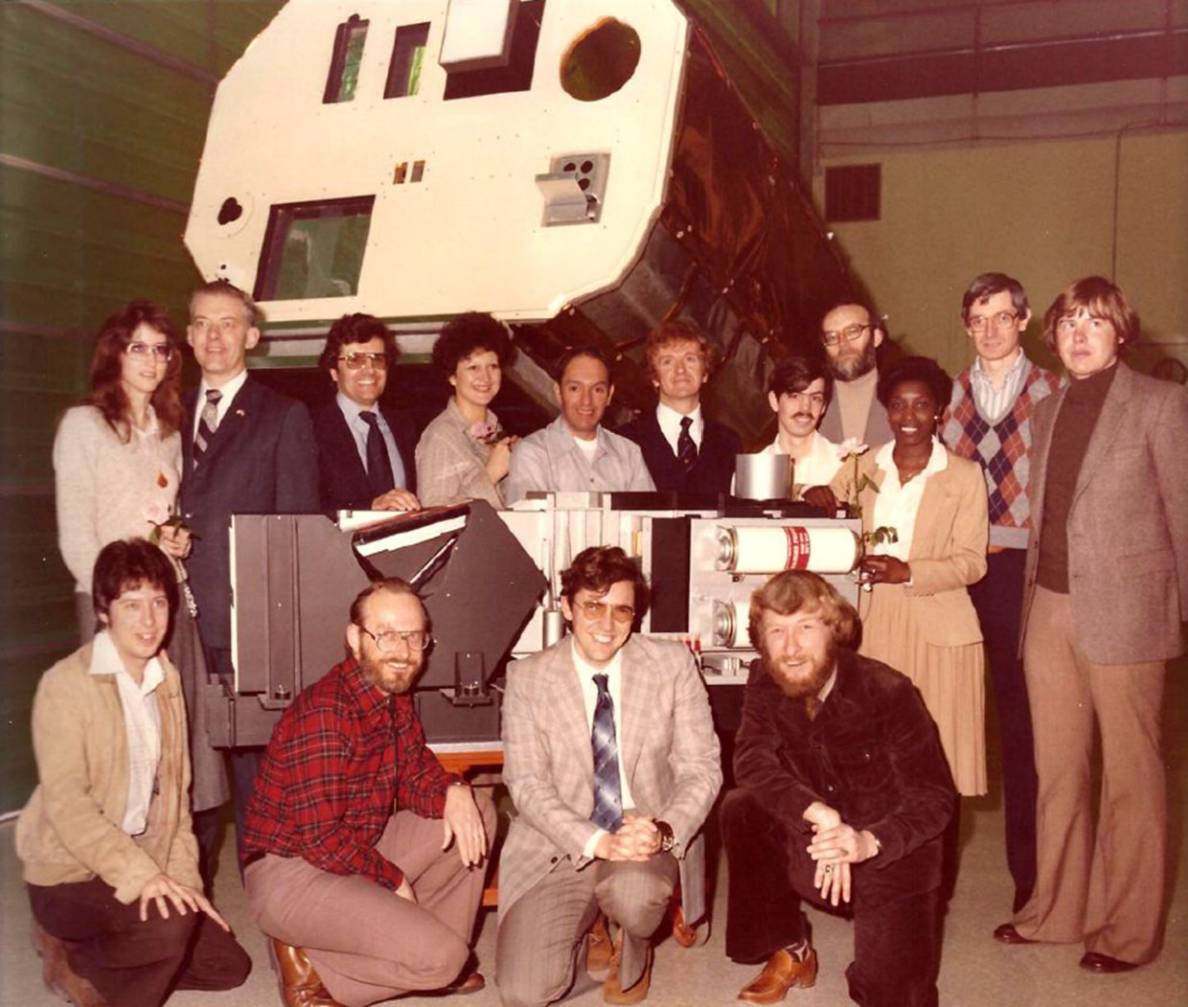
The XRP team at the EOF at the beginning of 1981. From the left standing: Becky Caffey, Kermit Smith, Chris Rapley, Ester Antonucci, Jake Wolfson, Ken Phillips, John Leibacher, Viola Duckett, John Sherman, and Bob Bentley; in the lower row: Nick Veck, Loren Acton, Keith Strong, and Roger Burdett.^20^.

Unfortunately, on November 19, 1980, following two previous gyro failures also the third of the four gyros of the attitude control subsystem failed and this ended the first phase of the mission, at least for the instruments with limited fields of view, such as XRP. The evening that this problem was discovered, in the role of the SMM chief planner, I received a dramatic call from GSFC. This was an awful moment since there was nothing we could do. I thought this was the end of XRP. The satellite could no longer be pointed with accuracy, thus after nine months of operations only the nonimaging instruments were able to continue their observations as the Sun was still observable in their field of view. In these moments, the only consolation was that XRP had already collected a wealth of flare spectra in its fifteen channels, thus the experiment’s success was ensured in any case. A picture of the XRP team that was present at the EOF in the first nine months of operations is shown in Figure 10.

**Figure 11:**
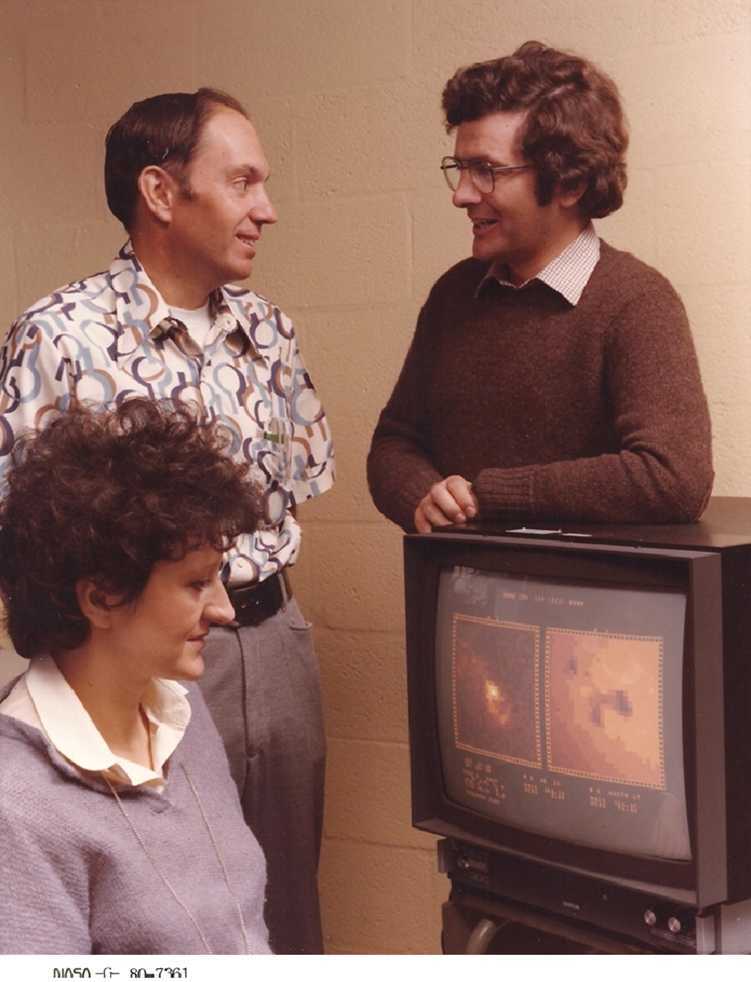
Experiment Operation Facility at Goddard Space Flight Center, 1980. The three XRP Deputy Principal Investigators, from right to left, Chris Rapley, Mullard Space Science Laboratory, Jake Wolfson, Lockheed Palo Alto Research Laboratory, Ester Antonucci, Rutherford Appleton Laboratory/University of Turin.

The days following this dramatic failure were days of great stress. The number of meetings increased up to seven in one day, and, as one of the three XRP deputy principal investigators,^19^ I had to attend most of them (Figure 11). I was convinced that there was nothing to do any longer, at least as far as our instrument was concerned. Instead, much to my surprise, since SMM was the first application of the Multi-Mission Spacecraft designed to be serviced by the Shuttle, very soon the idea of repairing the spacecraft in orbit was put forward by the US colleagues, even if the Space Shuttle was not yet ready to fly. After three years of preparation – part of the astronaut preparation took place right in building 7 – the repair mission indeed took place successfully. It was an incredible adventure for the time. SMM was repaired in orbit by the crew of the Space Shuttle Challenger on mission 41 C. The pilot of this mission, F.R. Scobee, was the commander of Challenger’s tragic last flight. For this reason, the book *The Many Faces of the Sun* (eds. Strong et al., 1999) is dedicated to his memory. The spacecraft-retrieval process turned out to be highly dramatic, but, in the end, successful. The spacecraft was repaired onboard the Shuttle and released in orbit on April 10, 1984. The second phase of the mission ended five years later in November 1989, with the spacecraft debris dispersed into the Indian Ocean. It was a success story, although with breath-taking moments, also considering that the spacecraft was designed for a minimum of one year of operational life. Keith Strong and Joan Schmelz describe in detail the dramatic phases of the repair enterprise in the introductory chapter of *The Many Faces of the Sun*.

### Science with the Bent Crystal Spectrometer of the Soft X-Ray Polychromator

#### Diagnostic-Tools Development

XRP was a complex experiment designed to measure the light emitted in the spectral range from 1 to 22 Å, where a large part of the energy output of flares is observed. The instrument included two subsystems, the Bent Crystal Spectrometer (BCS) and the Flat Crystal Spectrometer (FCS), for a total of fifteen spectroscopic channels (Acton et al., 1980). The BCS consisted of eight curved crystals simultaneously diffracting and dispersing photons into position-sensitive detectors. The very clever and innovative concept of using bent crystals made possible at once the detection at high resolution of the entire spectra of highly ionized heavy ions, each wavelength being reflected by a different part of the crystal surface. Thus, it was possible to trace the temporal evolution of the spectral emission with an unprecedented resolution.^21^ This combination of wavelength and time resolution was not achievable with traditional flat crystals, which could scan a given wavelength range only by changing the Bragg angle with time. This led to a breakthrough in the study of the rapidly changing plasma conditions characterizing the initial phase of flares, crucial to understanding the flare process. The 6$'\ \times $ 6′ field of view allowed one active region to be studied at a time. The BCS was an extremely successful instrument, whereas the use of the FCS was somewhat limited from the beginning of March 1980 onwards, due to a problem concerning the crystal drive.

XRP science benefited from the parallel observations and results of two almost simultaneous space missions: the US Air Force P78-1 and the Japanese Hinotori solar missions, launched in 1979 and 1981, respectively. The SOLFLEX instrument onboard the P78-1 satellite was designed to explore soft X-rays down to 1.8 Å, although with a somewhat reduced temporal resolution due to the use of traditional Bragg crystals (Doschek, 1983). The Hinotori instruments detected the wavelength region from 1.72 to 1.95 Å, scanned by utilizing the spin of the spacecraft itself (Tanaka et al., 1982 and references therein), and further extended the energy range of the hard-X-ray imaging up to 40 keV.

At the beginning of the XRP operations, there was a demand to further contribute to the development of the tools needed to interpret the data. Hence, I started to develop computer codes enabling us to interpret the spectra of the He-like Ca xix and Fe xxv,^22^ and the H-like Fe xxvi highly charged heavy ions detected in three distinct BCS channels. These spectra, in addition to the resonance, intercombination and forbidden transition lines, are densely populated by satellite lines formed in the process of dielectronic recombination or inner-shell excitation,^23^ situated on the longer-wavelength side of the resonance line (Gabriel, 1972). These lines represent a crucial diagnostic tool for measuring the electron temperature and the ionization conditions of the flare plasma.

The simplest approach to analyze the BCS data I adopted was to calculate a synthesized theoretical spectrum derived from a set of trial plasma parameters and to adjust the initial parameters (electron temperature, Doppler temperature, and relative population of adjacent ion stages) in order to best fit the observed spectra. This method made it possible to properly take into account the dependence of the line intensity on the presence of merging nearby lines (that is, the blending of all lines, as well as their width determined by thermal and dynamic plasma conditions and the Lorentzian instrumental profile characteristic of the crystal response).

During his visits at the EOF, Alan Gabriel guided me in the use of the atomic data relative to the ion transitions observed with BCS to be included in the computation of the synthetic spectra. By mid-March the codes were ready in a preliminary form, taking into account the most important satellites, sufficient for a first interpretation of the observed spectra. During the summer several visitors from Europe supported us. Visiting the EOF for an extended period, Jacques Dubau helped to improve the BCS codes to include the complete set of satellite lines, which became available thanks to the calculations of all relevant atomic transitions performed by the French–Belgian atomic physicists collaborating with Alan Gabriel (Bely-Dubau et al., 1982a,b).

#### First BCS Results

The first intense flare, detected on March 29, was immediately analyzed and a component of reduced intensity and blue-shifted relative to the principal component, moving at about 300 km s^−1^, was identified in the Ca xix spectra during the flare impulsive-phase. Moreover, the blue-shifted component was present throughout the hard-X-ray burst duration. This turned out to be a characteristic feature of the impulsive-phase Ca xix and Fe xxv spectra, which also showed pronounced nonthermal widths. In other words, these spectra were markedly different from those observed during the gradual phase, characterized by thermally broadened lines and the absence of blue-shifts. Further, no blue-shifts were found in flares close to the limb. These results were interpreted as evidence for plasma rising in the solar atmosphere, as predicted in the case of chromospheric evaporation. According to the theory, whenever the energy release in flares is so sudden that the chromosphere is unable to radiate it at a sufficiently high rate, the chromospheric plasma heats up to coronal temperatures and as a consequence the large pressure difference established between the dense chromosphere and the tenuous corona drives high-velocity upflows of large amounts of plasma (e.g., Antiochos and Sturrock, 1978). In this way, most of the flare energy is transformed into energy of the thermal plasma at temperatures above 10 million K and is radiated away predominantly in soft X-rays. In order to explain three soft-X-ray flares observed with OSO-III in 1967, Neupert (1968) first suggested as a likely interpretation of the data that ‘a portion of the chromosphere is heated to sufficiently high temperatures (as high as 20 – $40\times 10^{6}$ K) to account for the existence of the Fe xx-Fe xxv and is ejected into the lower corona’.

In the hypothesis that the blue-shifted emission was due to plasma rising in the solar atmosphere and accumulating in a magnetically confined coronal region, it was possible to verify that the observed mass and energy flows were sufficient to create the soft-X-ray source and to account for all radiation and heat-conduction losses. Hence, plasma upflows were beyond doubt interpreted as the manifestation of chromospheric evaporation (Antonucci et al., 1982a).^24^ It was also clear that the energy transported by the upflows was far more important than that appearing in the form of nonthermal turbulent motions. These results were preliminarily presented, together with the first well-resolved Fe xxvi spectrum emitted by a plasma region at 29 × 10^6^ K, at the Solar Maximum Year workshop in Crimea in March 1981 (Antonucci et al., 1982b). The two resonance transitions and dielectronic satellites of the Fe xxvi spectrum were first detected during the white-light flare of July 1, 1980 25 and fitted with a synthesized spectrum calculated according to the theory developed by Dubau et al. (1981). The indepth discussion of Fe xxvi spectra analysis is found in Parmar et al. (1981). Subsequently, in several flares Tanaka et al. (1983) measured temperatures of the thermal plasma above 30 × 10^6^ K with the Hinotori spectrometer.

The flare scenario that emerged during the first year of analysis was substantially confirmed by analyzing the large set of X and M flares detected in 1980 (Antonucci, Gabriel, and Dennis, 1984; Antonucci et al., 1985b). The complementary information – on the volume of the soft-X-ray source (3.5 – 8 keV) in the corona inferred from the Hard X-ray Imaging Spectrometer (HXIS) (Van Beek et al., 1980), on the energy released above 25 keV deduced from the Hard X-Ray Burst Spectrometer (HXRBS) (Orwig, Frost, and Bennis, 1980), and, in a few cases, on the loop footpoint geometry identified in the images of the hard-X-ray channel of the HXIS images, 16 – 30 keV (Hoyng et al., 1981; Antonucci, Marocchi, and Simnett, 1984) – made it possible to compute the total energy input by nonthermal electrons in the chromosphere in the assumption of thick-target interaction. The value obtained was fairly consistent with the soft-X-ray energy present at the coronal level as deduced from the XRP data. In other words, the energy input to the chromosphere in the form of fast electrons during the impulsive phase, which drives the evaporation process, was found to be of the same order of the thermal energy content of the soft-X-ray emitting plasma, which in turn was of the same order of the conduction and radiation losses observed during the flare-decay phase. The mass transfer into the corona was also consistent with the plasma density derived on the basis of considerations on the emission measure and on the volume where the plasma has propagated. These results on evaporation, including the X13 flare on April 24, 1984 observed in the second phase of the SMM mission, and the issues relative to energy deposition and transport processes were summarized in Antonucci (1989) and references therein.

Enhanced emission in the blue wing of the Ca xix and Fe xxv lines was also observed with SOLFLEX (Doschek et al., 1980; Feldman et al., 1980) and red-shifts in the Fe xxv spectra were observed by Korneev et al. (1980) with the Intercosmos-4 instruments, but the presence of persistent blue-shifts throughout the impulsive phase was not so unambiguously established and systematically detected, likely because of the somewhat limited temporal resolution of the classic Bragg spectrometers. Moreover, for the first time all satellite lines were properly taken into account in the BCS data analysis codes.

There was a heated debate about these results. Models of chromospheric evaporation predicted the blue-shift of the principal component of the resonance line itself and not only of a component of reduced intensity. However, only in a few cases was there evidence of an initial dominant blue-shifted component moving upward at low velocities present for a very short time. Another issue was that, in most cases, previous studies did not identify the blue-shifted component, probably because this can be easily masked by the blending of satellite lines with the red wing of the resonance line, possibly resulting in the spurious detection of an additional broadening of the resonance line. A further perplexity concerned the crucial question of whether the blue-shifted component was indeed capable of injecting sufficient hot plasma into the corona to justify the observed soft-X-ray source at the peak of the flare. A final important issue was whether the chromosphere was heated by nonthermal electrons accelerated during the primary energy release or by thermal conduction from a hot coronal source where energy is released.

In the three sessions of the Solar Maximum Mission Flare workshop organized at the GSFC and held between January 1983 to February 1984, one of the workshop teams addressed the topic ‘Chromospheric Explosions’. No other title could have been more appropriate. The team members were quickly drawn into lively discussions on the dynamics of the flare impulsive-phase and George A. Doschek, the team leader, very cleverly organized the team work as well as the conclusive paper in the form of a debate on three main controversial issues, with subteam coordinators arguing pro and contra a given interpretation (Doschek et al., 1986). The first issue concerned chromospheric evaporation, with George Doschek and myself as subteam coordinators. I learned a lot in these ‘explosive’ debates on different ideas and interpretations, although I remained convinced that the XRP observations identified the true signatures of chromospheric evaporation and that this was the main process involved in the formation of the soft-X-ray flare.

### Return to Europe

Due to the failure of the SMM pointing system, there was no reason for me to remain any longer at the EOF. Thus, I left Greenbelt at the end of January 1981 and continued the collaboration with Alan Gabriel in the UK. I rented an old cottage in a country village some 10 miles from RAL and from Oxford, on the way to the enchanting Cotswolds region. In my village the microclimate was much milder and less windy than in Chilton. The winter of 1981 was quite unusual: extremely cold and with a lot of snow, which caused a great deal of trouble in a country not used to such harsh winters and driving on snow. The following year, at the end of February, I was back in Italy where I resumed teaching my courses in experimental physics at the university, with a promotion to Associate Professor in 1983. Thus, my ‘space’ adventure as part of the XRP team at the EOF ended, but not the work on the BCS data, the collaboration with Alan Gabriel and the frequent visits at the GSFC in the second phase of the mission.

**Figure 12:**
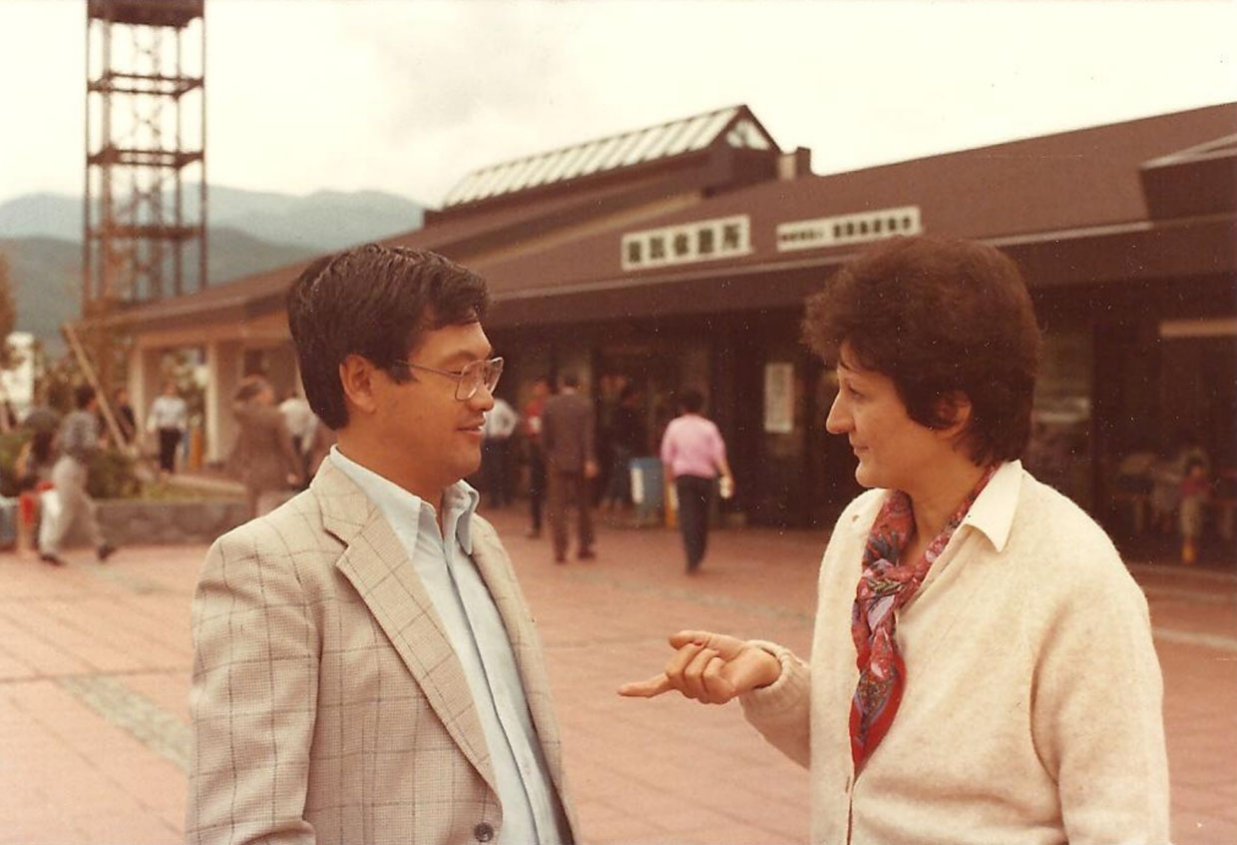
Symposium on Solar Flares, 1982. Trip to Nobeyama. Katsuo Tanaka and Ester Antonucci (picture taken by Kees de Jager).

In these years there were many workshops, and conferences dedicated to the solar maximum physics entailing a lot of traveling within European countries and in the US. After the Crimea meeting in March 1981, I was invited for the second time to the USSR for the International Workshop on Solar Maximum Analysis in Irkutsk, Siberia, in 1985. This was quite interesting since the USSR pioneered the soft-X-ray spectroscopy of the highly ionized Fe ions (Grineva et al., 1973) and there was great interest in the BCS results. There was a lot of interest in the SMM results in the Asian countries as well. I attended my first meeting in Tokyo in October 1982 (Figure 12). At that meeting the scientists taking part in the discussion of the presentations had to state their name when they spoke, since the comments were recorded for publication purposes. Worried because during my first comment I had forgotten to say my name, a colleague reassured me that I was certainly going to be recognized, as the only woman at that meeting. It was certainly not the last time that this situation occurred.

Japan was starting a very successful story of space missions dedicated to solar physics with the launch in 1981 of Hinotori, leading to important contributions in soft-X-ray spectroscopy of the active corona thanks to Katsuo Tanaka. Thereafter, the Yohkoh mission was launched in 1991 with instruments suitable for continuing soft-X-ray flare spectroscopy and the Hinode mission in 2006. In the last letter that I received from Katsuo Tanaka, aware of his hopeless health conditions, he expressed his wish to live at least until the launch of Yohkoh, but his wish was not fulfilled. Very sadly, this letter arrived to me the very day I learned of his death.

I was also invited to present the BCS results at the IAU General Assembly in New Delhi in November 1985 and one year later in November 1986 at the International Symposium on Space Physics in Beijing. The meetings in India and China were an immersion in completely new dimensions. Visiting China, I was aware that this was a country with significant potential, considering its great culture with roots dating back a few thousand years. However, it was difficult to imagine the rapid and astonishing development of space science and technology that would occur in the next few decades.

#### A Sad Epoch

After Tanaka’s death, in the span of just a few years, from 1995 to1997, we sadly lost a number of dear colleagues and friends who had greatly contributed to solar science from space and passed away while still too young: Chung-Chieh Cheng of NRL, Bruce Patchett of RAL, and Brunella Monsignori Fossi of the Arcetri Astrophysical Observatory. They are commemorated in: ‘Remembering Brunella Monsignori-Fossi’ (Landini, 1997); ‘Dedication to Bruce Patchett’ (Gabriel, 1997); ‘Dr. Chung-Chieh Cheng’s Contributions to Coronal Physics’ (Wu, 1997).

#### Further Science with the XRP Data

The studies of soft-X-ray flares with XRP data continued in parallel with the interest in future space missions until 1995, the year of the launch of the second mission I was involved in: the ESA-NASA SOHO mission. The main lines of research addressed in flare physics were still concerned with the interpretation of the two unambiguous systematic signatures of the impulsive phase: the nonthermal line broadening, much enhanced with respect to that observed in quiescent active regions, and the significant blue-shifted emission, evidence for the dominant mode of mass and energy supply to the corona during flares. These signatures revealed that the soft-X-ray emission in the 1 – 22 Å range was not only a manifestation typical of the gradual phase, as had been considered before the SMM era, but was a powerful means to investigate the impulsive phase of flares.

#### Soft-X-Ray Line Broadenings During Flares

The soft-X-ray line broadening observed with BCS throughout the impulsive phase was addressed on the basis of two models, both based on the process of magnetic reconnection. In both cases, we attempted to explain the source of the excess widths in the preflare and flare regimes as associated with the primary energy release.

In collaboration with Robert Rosner and Kanaris Tsinganos of the Center for Astrophysics (CFA) in Cambridge, Massachusetts, the line broadening was interpreted in terms of their model of local heating resulting from reconnection due to magnetic-field line stochasticity. In this scenario, the turbulent broadening of the spectral lines emitted from the high-temperature plasma could be entirely explained as due to the outflowing motions from many reconnection sites where particles were accelerated and scattered throughout the flaring loop. This suggestion was dictated by the symmetry of line broadening (isotropic turbulence), which likely occurs in preexisting coronal material, filling about 5% of the total volume seen in soft-X-ray emission (Antonucci, Rosner, and Tsinganos, 1986).

Another scenario based on the same process was then proposed with Boris Somov of Moscow State University. Line broadenings could be interpreted as a signature of magnetic reconnection in current sheets forming in active regions that provide the energy necessary for generating solar flares, as well as the kinetic energy of fast hydrodynamic flows and jets due to plasma ejected in opposite directions from the reconnecting current sheet with a velocity comparable to the Alfvén velocity (see the book *Physical Processes in Solar Flares* by Boris Somov, 1992). If many reconnecting current sheets operate at the same time, the effect of the jets would be observed as a symmetrical line broadening significantly larger than the thermal line broadening. The largest broadenings are expected at the rupture of the current sheet in connection with the transition from the preflare to the flare regimes (Antonucci, Dodero, and Somov, 1993). A comparison of nonthermal broadenings of the Fe xxv emission observed at flare onset with the predictions of the high-temperature turbulent current sheet model (Somov, 1992) suggested the presence of either several small-scale or a few large-scale reconnecting current sheets with internal temperature ^≤^ 80 × 10^6^ K in the flare region. In this model, the velocities of the plasma emerging from the reconnection site, inferred to be ^≤^ 1100 km s^−1^, depend on the temperature inside the current sheet. The number and/or geometrical complexity of the reconnecting current sheets ensures the substantial isotropy of the velocities in the flare region such that the observed nonthermal velocities are independent of flare longitude (Antonucci, Benna, and Somov, 1996).

#### Comparison of Flaring Loops with Simulations

During the SMM mission a large effort was dedicated to performing simulations of the hydrodynamics and magnetohydrodynamics of flaring loops and to comparing the spectral emission resulting from these simulations to the soft-X-ray spectral observations in order to discriminate between possible physical flare models, in particular between thermal and nonthermal models. The different approaches are summarized in Chapter 10 of *Many Faces of the Sun* (Strong et al., 1999).^26^ Simulations of the profiles of the individual spectral lines were first performed by Doschek et al. (1983).

Following the SMM workshop, in collaboration with the Palermo group led first by Giuseppe Vaiana and then, after his death in 1991, by Salvatore Serio, we analyzed how the plasma in a coronal loop responded to the deposition of energy. The hydrodynamic simulations were performed on the basis of the Palermo–Harvard numerical hydrodynamic model for a magnetically confined plasma (Peres et al., 1982). Comparing simulations with observations we concluded that in the case of thermal heating, evaporation represents the most important process in generating the flare soft-X-ray source (Antonucci et al., 1987b), whilst the electron-beam model did not fully reproduce the flare soft-X-ray line profiles. Electron beams with soft spectra and low-energy cutoff, most closely resembling thermal heating, were the ones more consistent with the observations (Antonucci et al., 1993 and references therein).

In addition to the simulations of flaring loops, we continued to pursue phenomenological studies of the evaporative plasma flows. In a number of very energetic flares, such as the class X13 flare of April 24, 1984, impulsive phase upflows characterized by a broad velocity distribution with a tail at a very high velocity (up to 1000 km s^−1^) were detected in the Fe xxv spectra (Antonucci, Dodero, and Martin, 1990a). In the case of this extremely energetic flare, it was also possible to find successive injections of very hot plasma of 30 – $40 \times 10^{6}$ K. In any case, the emission measure of the high-velocity tail turned out to be relatively unimportant in the energy budget of the flare.

Several other interesting results were achieved. Examples worth mentioning are: the velocity–temperature distribution in the evaporating plasma (Antonucci, Dodero, and Martin, 1990b), the law relating nonthermal velocities derived from the soft-X-ray lines and plasma temperature (Antonucci and Dodero, 1995); the presence of a superhot coronal condensation at about 40 × 10^6^ K identified in very impulsive events both on the basis of the Fe xxvi spectra (Tanaka et al., 1982) and on a differential emission measure analysis (Martin, Antonucci, and Somov, 1993); and the Fe and Ca abundance values derived from the XRP data that were indicative of the existence of Ca-rich and Fe-rich flare events (Antonucci and Martin, 1995). Using XRP and SOX-Hinotori data, we also proposed a revised ionization balance for iron, based on the relative abundances of Be-like and Li-like ions to He-like ions (Antonucci et al., 1987a), as well as for calcium based on the relative abundances of Li-like ions to He-like ions (Antonucci et al., 1984). In addition, we derived the argon/calcium abundance ratio on the basis of XRP and P78-1 data (Antonucci et al., 1987c).

Even at the time of this writing, no other instrument has provided soft-X-ray spectroscopy of the flaring plasma as complete as that of the XRP and in this sense some of the XRP results remain unique. The main results on flare dynamics obtained by the high-resolution soft-X-ray spectrometers operating during Cycle 21, including XRP, are summarized in the Chapter 10 on ‘Flare Dynamics’ in *The Many Faces of the Sun* (Strong et al., 1999).^27^

## SOHO – the First European Solar Space Mission

### From the SOHO Proposal to the Spacecraft Launch

Chatting at RAL with Bruce Patchett I learned that he was working on a proposal entitled Solar High-resolution Observatory (SOHO) to be submitted in response to an ESA call for ideas for new missions in November 1982 (proposers: M. Malinovsky-Arduini, H.F. van Beek, J.-P. Delaboudinière, M.C.E. Huber, P. Lemaire, and B. Patchett). The intention to respond to this call was at first discussed at a meeting in Paris by Roger Bonnet, Monique Arduini, Martin Huber, Bruce Patchett, and Alan Gabriel. The aim of the mission was the study of the physical processes involved in heating the solar corona and accelerating the solar wind, basic mechanisms that are still not fully understood. SOHO also included the objectives of the Grazing Incidence Solar Telescope (GRIST) mission studied by ESA at phase-A level. As the great opportunities that a European solar space mission could offer became clear to me, I became interested and then involved in the long-term SOHO venture.

During the ESA assessment study, from February to August 1983, the original scientific goals of SOHO were merged with those addressed during the phase-A study of the mission named Dual Spectral Irradiance and Solar Constant Orbiter (DISCO), proposed to investigate the interior of the Sun via helioseismology. The purpose then became to study the Sun from its deep core to the outer corona as well as the solar wind in the corona and in the heliosphere, with a payload including in situ instruments in addition to the remote-sensing ones. A continuous view of the Sun and a low relative velocity between Sun and spacecraft, which could be achieved by choosing a halo orbit around the first Lagrangian point L1 (at a distance of 1.5 × 10$^{6}\text{ km}$ from Earth), was considered essential in order to best address this broader spectrum of scientific objectives. The mission was then renamed Solar and Heliospheric Observatory. The phase-A study was conducted from November 1983 to December 1985, with the final presentation of the results in January 1986. The spacecraft industrial study^28^ was carried out in the assumption that the mission could be developed in collaboration with NASA. At a certain point, ESA also decided to conduct a rider study to the phase-A study, aimed at considering as an alternative a solely ESA mission with reduced payload in case the International Solar-Terrestrial Physics (ISTP) program were not approved.^29^ At this point, I was part of the science team of the rider study, contributing to the definition of the model payload of the reduced SOHO version.

When in 1983 Roger Bonnet was appointed Director of the scientific program of ESA,^30^ he acted to strengthen ESA science by setting up a grand long-term plan of scientific investigations from space that could attract sufficient financial resources to be fully implemented. In a short time, his effort led to the definition of ‘Space Science – Horizon 2000’, a well-structured and well-balanced program aimed to promote a vigorous growth of space research in Europe. In this scenario in 1984, SOHO was identified as part of the first cornerstone of the Horizon 2000 program together with Cluster, a four-spacecraft mission designed to study plasma structures in three dimensions. This was a great achievement for the European solar community. The final decision on the first cornerstone was taken in February 1986 when it was decided that SOHO was to be implemented in collaboration with NASA, responsible for the launch and the operations and to be included in the ISTP program, of ESA, NASA and ISAS, Institute of Space and Astronautical Science, the Japanese space agency.^31^ Following the announcement of opportunity issued on March 1987 the payloads were selected on March 1988.^32^ The industrial phase B started in October 1989. From January 1986 to 1988 I was able to witness the mission advancements as a member of the ESA Solar System Working Group.^33^ The eventful history of the SOHO mission is reported in great detail in Huber et al. (1996).

**Figure 13:**
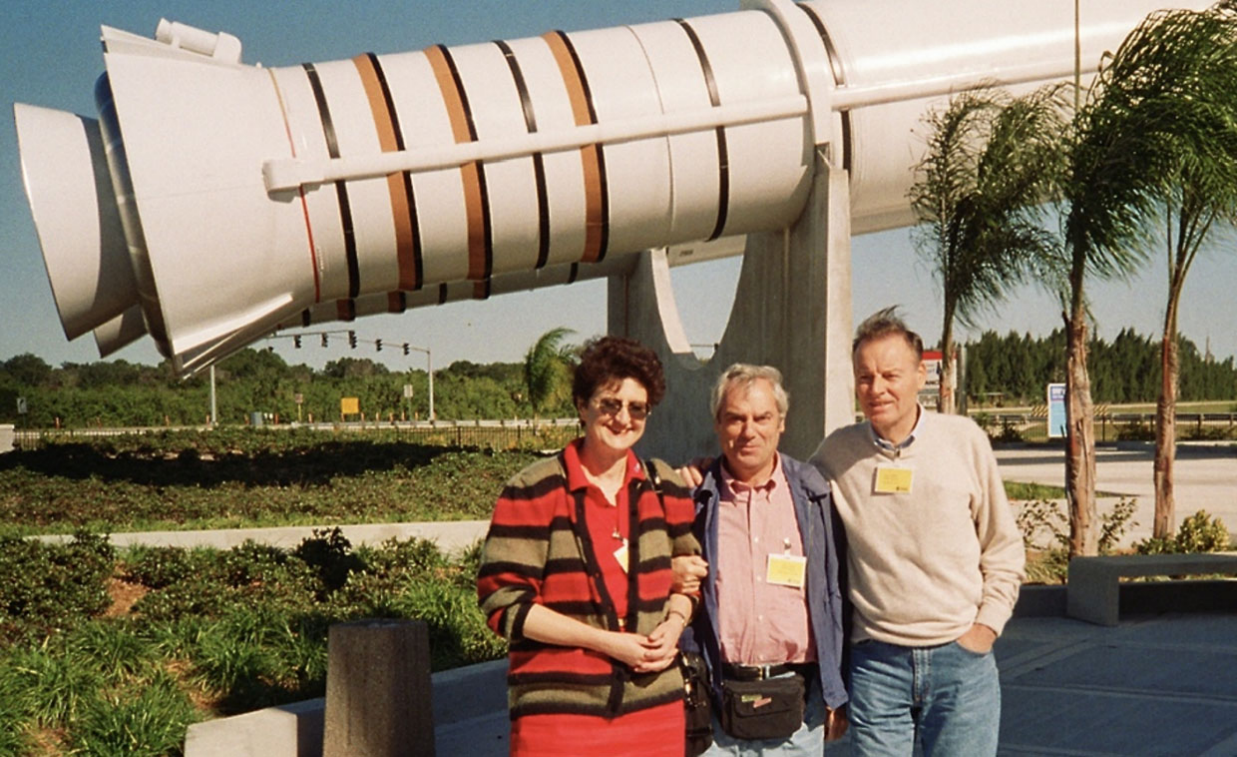
Waiting for the SOHO launch at Cape Kennedy, November 1995. From the right: Giancarlo Noci, Giuseppe Tondello, and Ester Antonucci.

SOHO became the third large solar observatory in space, after Skylab and SMM. Its payload turned out to be the most comprehensive set of solar and heliospheric instruments ever placed on the same platform, for a total weight of 640 kg. The spacecraft was launched on December 2, 1995 from Cape Kennedy (Figure 13), only six years after the end of the SMM mission. The spacecraft entered a halo orbit at L1 on February 14, 1996, exactly on the sixteenth anniversary of the SMM launch. The SOHO payload is described in the volume of *Solar Physics* dedicated to the SOHO mission (Editors: Domingo, Fleck, and Poland, 1995).

Like SMM, SOHO was affected by an unfortunate event. The spacecraft was lost after about two and a half years of operations, at the end of June 1998. The failure was due to operational problems. After various efforts to reestablish contact with SOHO, a scientist coming from the SMM experience, Alan Kiplinger, suggested the successful idea to try to locate the spacecraft with the support of the Arecibo radar. On July 28, 1998 the Arecibo–Goldstone bistatic radar configuration confirmed that SOHO was still in its expected position and was not rotating excessively fast.^34^ The first signal from the spacecraft was received on August 3: SOHO was still alive. The spacecraft was reoriented and the Sun-pointing attitude was fully recovered in September 1998. At the end of December of the same year the last gyroscope was lost but the engineers were able to overcome the problem and SOHO became the first three-axis stabilized spacecraft operated without gyros. With the exception of the 1998 summer of distress, SOHO continued its incredible success story for more than two and a half solar cycles under the guidance of the ESA project scientists: initially Vincente Domingo and later Bernhard Fleck. Designed for a two-year lifetime, eight of the twelve SOHO instruments are still operational at the time of this writing. In the first twenty-five years, more than 6000 papers based on SOHO data have been published in the refereed literature and more than 5000 scientists have been involved in data analysis.

On October 21, 2020, the PROSWIFT (Promoting Research and Observations of Space Weather to Improve the Forecasting of Tomorrow) Act, signed into law by the President of the United States, after it had passed both chambers of Congress, declared SOHO an infrastructure of critical importance to the nation’s space-weather architecture.^35^

### The UltraViolet Coronagraph Spectrometer

The idea of participating in the payload development started to come to my mind when SOHO entered the assessment study. It would have been quite natural for me to be part of the science team of the Coronal Diagnostic Spectrometer (CDS) led by Bruce Patchett at RAL, or of the Solar Ultraviolet Measurements of Emitted Radiation (SUMER), accepting a few years later an invitation by Ian W. Axford, Director at the Max Planck Institute. However, I thought it was perhaps the right time to undertake some action in view of the design and realization of the SOHO payload with other interested Italian colleagues, rather than being exclusively interested in the SOHO science.^36^

The payload designed during the SOHO phase-A study included a UV coronagraph in order to detect the source regions of the solar wind in the atmosphere of the Sun with a new spectroscopic technique. Martin C.E. Huber of the Technische Hochschule, Zurich, chair of the ESA Solar System Working Group at the time of the SOHO assessment and phase-A study, brought to the attention of the study teams the concept of a novel EUV coronagraph based on Doppler dimming. The idea of determining the outward expansion of the hot corona by measuring the Doppler dimming of the resonantly scattered UV lines – first the dominant H i Ly$\alpha $ line at 1216 Å emitted by neutral hydrogen atoms – was put forward in the 1970s by Giancarlo Noci of the University of Florence (as quoted by Withbroe et al., 1982), who later also suggested the diagnostic method based on the O vi 1032 – 1037 doublet, which turned out to be crucial for extending the range of the measurable velocities of coronal outflows (Noci, Kohl, and Withbroe, 1987). John Kohl, who had started developing at CFA a UV coronagraph-spectrometer based on Noci’s idea, was one of the US scientists supporting the SOHO phase-A study team, which also included Giuseppe Tondello of the University of Padua, an expert in laboratory UV spectroscopy. In July 1983, John Kohl visited Florence to discuss with us the opportunities offered by SOHO to pursue the new approach to ultraviolet coronagraphy, which exploited the Doppler-dimming diagnostics.

In the opinion of both Tondello and myself, the best way to get Noci fully involved, not only from the scientific point of view, but also from that of the hardware development in the SOHO project was to join forces and collaborate with Kohl and Huber in proposing a UV coronagraph with spectroscopic capabilities, never flown before on a long-term space mission. Our intent became to contribute to the coronagraph with the study and development of the spectrometer subsystem. Thus, in 1983, the trio formed of Noci, Tondello, and myself, quite complementary in terms of competence and personality, initiated a challenging and I would say successful experience. Starting in 1984 we held numerous meetings with leading figures in Italian industry – at first a bit hesitant to be involved in the development of scientific hardware – with the Italian Space Office to ensure the needed endorsement and financial support, and with Kohl’s team to define the partnership and to contribute to the instrument design.

**Figure 14:**
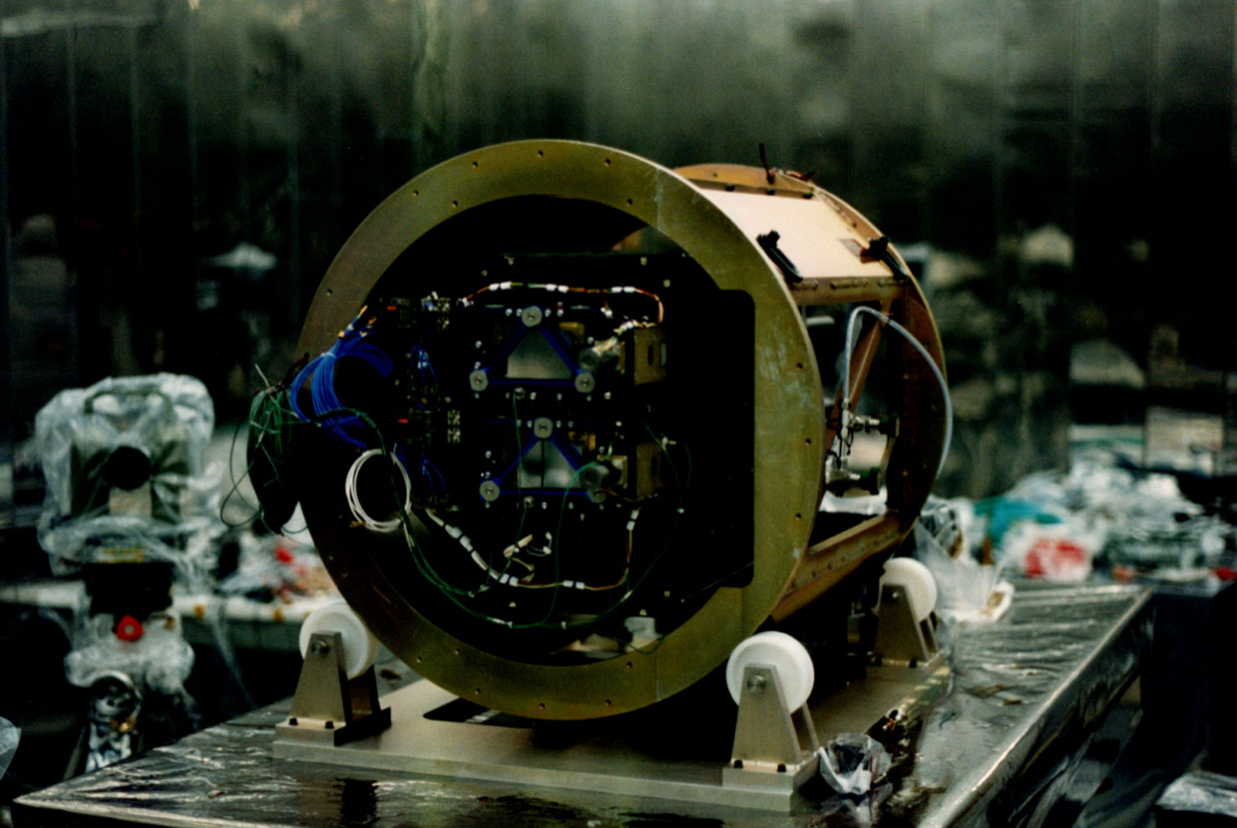
The spectrometer subsystem, Italian contribution to the UVCS, on the Alenia Space premises in Turin.

The description of the main features of the UV coronagraph for inclusion in the model payload dossier was ready by January 1986. During the Initiation Meeting with Kohl, held at the CFA in March 1987, the baseline design and the definition of the main tasks to be carried out in Italy by Aeritalia^37^ in Turin and Officine Galileo^38^ in Florence were agreed upon in view of the submission of the proposal of the Ultraviolet Coronagraph Spectrometer (UVCS). The best and final UVCS proposal based on the revision of costs, complexity and weight requested by NASA was submitted in January 1988. A decisive meeting concerning the contribution of the spectrometer subsystem was held at the Space Office of the Ministry for Scientific Research in Rome in the presence of the three of us – Noci, Tondello, and myself – on December 30, 1987, in order to be able to submit in time the revised proposal with the full approval of the Italian part. The meeting turned out to be quite positive, with a more than substantial increase in terms of financial support (unfortunately I had to forget my ‘white’ week of crosscountry skiing in the Alps!). In the best and final proposal, the Italian Space Agency (Agenzia Spaziale Italiana, ASI)^39^ was responsible for providing the spectrometer of the UVCS coronagraph (Figure 14), with Kohl and Noci in the roles of UVCS Principal and Co-Principal Investigator, respectively.

To become a US instrument supported by NASA with ASI and Swiss participation, the first step in the UVCS selection procedure was to undergo a preliminary NASA evaluation, which turned out to be negative: UVCS was not recommended for funding-allocation reasons. Notwithstanding this initial very serious problem, the proposal was later approved during the ESA selection process, with the justification that UVCS was crucial to meeting SOHO’s scientific objectives and the Italian scientists and resources were important to the ESA Scientific Program.^40^ In the end, as many as four coronagraphs were onboard SOHO: the three coronagraphs of the LASCO suite, built by the Naval Research Laboratory, the University of Birmingham, the Max Planck Institute fur Aeronomy, and the Laboratoire d’Astronomie Spatiale (Marseille), observing for the first time the corona from 1.1 to 30 solar radii (Brueckner et al., 1995), in addition to UVCS for the UV spectroscopy of the solar-wind acceleration region from 1.5 to 10 solar radii (Kohl et al., 1989; Kohl et al., 1995a, 1995b). In Italy, an industrial team comprised of Alenia Space in Turin and Officine Galileo in Florence was involved in the realization of the UVCS.

**Figure 15:**
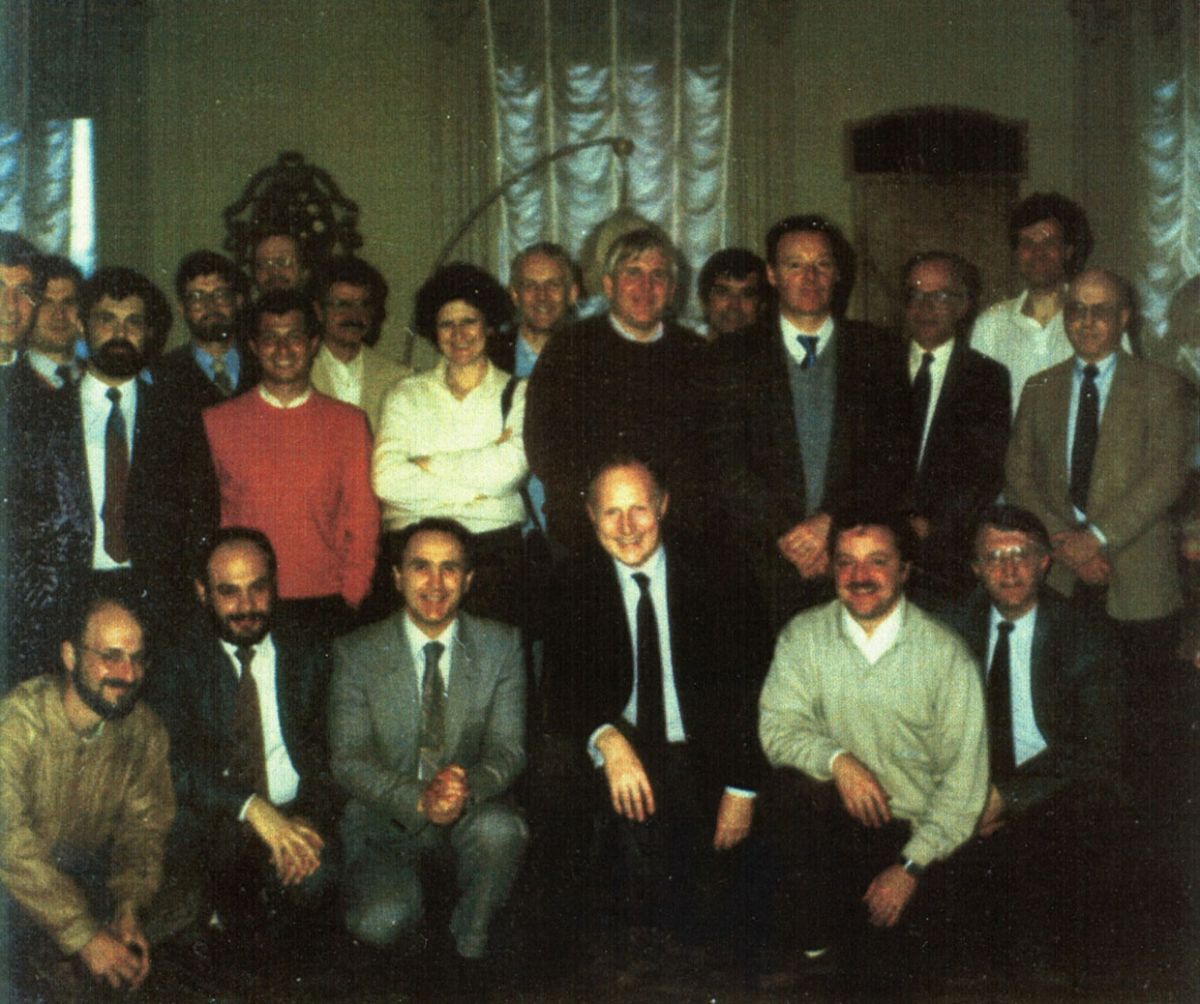
First UVCS team meeting after payload selection held in Turin on April 11 – 13, 1988. From the left, standing scientists: Daniele Spadaro, Joseph V. Hollweg, Fabio Reale, Ester Antonucci, George L. Withbroe, John L. Kohl, Marco Malvezzi, Giancarlo Noci, Roger Kopp, and Heinz Weiser. Scientists in the lower row from the left: Alfred Buergi and GianPaolo Tozzi; from the right: Joseph Lemaire, Stefano Livi, and Martin C.E. Huber. Engineers from industry also formed part of the team and included in the picture.

At the end of March, Riccardo Giacconi, Nobel Prize winner in 2002 for his work in X-ray astronomy, was invited to the Alenia premises to attend a presentation of the scientific space projects under industrial development in Turin: the satellite SAX^41^ and the UVCS spectrometer. On this occasion he demonstrated great interest in UVCS and this was very encouraging at the start of this new undertaking. Soon after, during the kickoff meeting that took place in April 1988 at the Physics Institute in Turin, we duly celebrated the UVCS selection with the US and Swiss colleagues (Figure 15). The first SOHO workshop was held in Annapolis in August 1992, where a brief description of UVCS was presented (Kohl and Noci, 1992; see also Noci et al., 1994).

The progress of the UVCS in the development phase was not quite straightforward, since UVCS was a complex and heavy instrument, including a series of mechanisms, such as the mechanism needed to roll the heavy structure around the axis of the instrument in order to allow the observation of different sectors of the corona so that an image of the full corona could be acquired with subsequent rolls. The guidance of NASA, ESA, and ASI and the endurance of the Italian trio, together with Martin Huber in supporting the Principal Investigator, turned out to be crucial to overcoming the various technical and managerial difficulties and in the end to deliver an instrument only slightly descoped with respect to the initial design.

UVCS successfully operated for more than one solar cycle, from 1996, when SOHO reached L1, until 2013. The wealth of spectroscopic data collected with UVCS has not yet been fully exploited and still contains a lot of information to be mined.

### The Parentheses of the Orbiting Solar Laboratory and the EURECA Platform

#### X-Ray UltraViolet Imager for the Orbiting Solar Laboratory

In the year of the UVCS approval, NASA invited ASI to take primary responsibility for the X-ray UV Imager (XUVI) for the Orbiting Solar Laboratory mission (OSL). The invitation letter by L.A. Fisk, Associate Administrator for Space Science and Applications, NASA, was sent in October 1988. One year later OSL was declared a candidate for a new start in the fiscal year 1992, with launch foreseen within the time frame 1998 – 1999. Thus, thanks to the experience gained from UVCS, it was time for me to take responsibility for this new interesting undertaking.

OSL was designed as a satellite in polar orbit around the Earth with one main scientific element: a meter-class telescope working in the visible and near-UV range, a project led by Alan M. Title of the Lockheed Research Laboratory. The other two complementary instruments foreseen onboard were the high-resolution UV spectrograph (1200 – 1700 Å) led by Gunther E. Bruckner of the NRL, and the X-ray UV Imager (40 – 400 Å), led by myself. The other two Principal Investigators could count on their long experience as space scientists, and I could count on their support, and on the precious advice of US coinvestigators such as Marylin E. Bruner of the Lockheed Research Laboratory, Leon Golub of the CFA, Roger J. Thomas of the GSFC, and Donald F. Neidig of the Phillips Laboratory of the US Airforce. On the Italian side, I had the full collaboration of Marco Malvezzi from the University of Pavia and Luigi Ciminiera of the Politecnico di Torino, with part of the UVCS team.

The XUVI, a normal-incidence telescope with mirrors coated with multilayers, was an ambitious instrument and working on its definition was quite an instructive effort. The multilayer mirror technology was new and had been tested in rocket-flight experiments. The instrument was comprised of two complementary units: the high-resolution imager resolving 0.25 arcsec (pixel size smaller than 200 km) on a limited field of view and the full-disk imager resolving 2.3 arcsec, each acquiring high-resolution images in UV spectral bands capable of covering a wide plasma-temperature range; in other words, the instrument was designed to allow the simultaneous observation of all layers of the solar atmosphere from the cool chromosphere and transition region to the hot corona, detecting the XUV radiation from plasma emitting at the various temperature regimes from 10^5^ K to a few 10^7^ K (Antonucci et al., 1992). One of the main goals was to get closer to resolving the individual magnetic fluxtubes present in the solar atmosphere. The phase-A/B study of the instrument was approved by NASA during a successful review that took place in Turin.

The participation in OSL was a positive though brief experience, with its most pleasant moment when Daniel S. Spicer and I organized the OSL workshop on the island of Capri, in May 1991, addressing the mission scientific objectives.^42^ Unfortunately, everything ended a few months later, at the time of the Iguazu solar-physics meeting in Argentina at the beginning of July. The news of the selection of the High Energy Solar Spectroscopic Imager (HESSI) mission instead of OSL as the new entry in the NASA scientific program was announced to the audience of the meeting during an afternoon session, just a few seconds before my talk on the XUVI project. It was shocking, but I decided in any case to present the work done in defining our innovative instrument, and before dinner I did not miss the opportunity to toast the good fortune of HESSI with Brian Dennis, one of my old SMM friends. After all, the selected mission, later renamed Reuven Ramaty High Energy Solar Spectroscopic Imager (RHESSI) was continuing the SMM tradition, dear to me, and it turned out to be extremely successful. In any case, the OSL effort was not a total loss, since other missions have profited from the OSL concepts and studies.

While OSL was on hold, the XUVI was also discussed in the frame of possible scientific utilization of the Space Station and presented in a simplified configuration as a EUV complementary instrumentation (Antonucci, 1992) for SIMURIS, Solar, Solar System and Stellar Interferometric Mission for Ultrahigh Resolution Imaging and Spectroscopy,^43^ a mission concept proposed by Luc Damé.

#### MGS – the Multilayer Grating Spectrometer

In February 1991 ASI organized a workshop in Rome with the intent to stimulate and coordinate the interests of the scientific community on the opportunities offered by the flight of the European Retrievable Carrier (EURECA-3) foreseen within the Space Station Columbus Precursor Flights program. Being three-axis stabilized and pointing at the Sun, in my opinion the EURECA platform offered interesting opportunities for testing new technologies for solar telescopes. Thus, with the colleagues already involved in XUVI, in March 1991 we submitted to ESA a proposal (for which I was responsible) to fly a new kind of instrumentation, the Multilayer Grating Spectrometer (MGS). The proposal was selected by ESA and the accommodation study on EURECA-3 was carried out in 1991. The instrument-interface issues were discussed in detail in a meeting at ESTEC in view of a phase-B completion foreseen at the end of 1992 and of a 1996 flight.

The scientific goal of the MGS was to acquire high spatial and spectral resolution monochromatic images of the full Sun in each of the intense lines emitted in the spectral region 170 – 230 Å, corresponding to the temperature regime between 10^5^ and 10^7^ K, in order to derive density, temperature, and velocity maps of the full solar disk with a spatial resolution of 10 arcsec. The novelty consisted in covering a standard grating with a suitable multilayer coating to enhance the normal incidence reflectivity so that an entire high-resolution spectrum in the selected wavelength window could be acquired for each spatial pixel. A full solar map could be acquired in less than 60 minutes. MGS was based on a technological innovation devised by Thomas et al. (1991), who manufactured at the GSFC a multilayer grating for an XUV normal incidence telescope that could achieve very high spectral resolution. In addition to the benefits in terms of expected solar-physics results, the participation in the EURECA project offered the opportunity to test the long-term performance of this new technology for optical components in the soft-X ray/UV domain, since the instrument could be retrieved and analyzed in the laboratory after flight.

However, following the first one, the EURECA flights provisionally scheduled in 1992 – 1993 were canceled. In order to take advantage of this experience, in the following years we also pursued a study for accommodating this instrument on an Express Pallet of the Space Station. Although the Space Station did not ensure the best operational conditions for such an instrument, the proposal was formulated and submitted to ESA in 1997, but it was not selected. In the end the MGS (Antonucci et al., 1998) was never built and never flown.

In a sense, neither the XUVI-OSL nor the MGS-EURECA experiences were a failure. They were instead excellent training opportunities that prepared me for the role of Principal Investigator of the UV coronagraph of the Solar Orbiter, my last space mission.

### An Unexpected Surprise

In 1994, the year before the UVCS launch, I was very honored to be nominated a corresponding member in the International Academy of Astronautics during the Academy meeting in Jerusalem. The list of the members of the Basic Sciences section included only one Italian name at that time, Giuseppe Occhialini, who, however, had passed away in 1993.^44^ This was the first recognition I received from an international academy. It was also very important for me because it gave me the opportunity to meet some of the pioneers of the early phase of the space adventure that had begun in the twentieth century. I have always been convinced that the generation of scientists and engineers preceding my generation was really an exceptional one, and that we owe to them infinite gratitude and admiration.

### UVCS – SOHO Scientific Planning at the EOF

The first year of observations with UVCS was again a busy period of traveling back and forth to the US. I quite frequently visited the SOHO Experiment Operation Facility in building 1 at the GSFC. At the EOF the experiment teams were planning the observations, sending the commands and receiving the data from the instruments in an almost real-time manner. A good share of the UVCS team at the EOF was formed by young Italian graduate students and postdoc researchers from Florence, Turin, Padua, and Palermo, sent to collaborate with Kohl’s staff. On January 29, 1996, two weeks before the insertion of SOHO in the halo orbit at L1, the detectors of the UVCS were switched on. Although preliminary scientific data were acquired even before, regular scientific operations began in April. In the first year of observations the outer corona showed a quite regular dipolar configuration, a perfect example of solar-minimum corona, especially suitable for studying the transition from the fast to the slow wind in the quiet solar atmosphere. In the first week of science planning, from April 1 to April 9, I had the privilege of playing the role of lead observer. We verified and optimized observing sequences, starting with the observation of the plasma dynamics in polar coronal holes and tested the first full synoptic sequence, prepared by John Raymond of the CFA, aimed at obtaining daily images of the global corona and the solar wind right in its acceleration region, via successive scans in altitude at a given latitude and rolls of the coronagraph to observe the entire corona. After several weeks of almost continuous presence at the EOF the team needed a rest, at least at Easter, thus I suggested that we could prepare and upload a 72-hour long polar-observation sequence. The Easter break was rewarded with an unprecedented observation of polar plumes in the outer corona, extending out to about 2 R_⊙_ (Antonucci et al., 1997b; Giordano et al., 1997). By June 1996 several Joint Observing Programs developed in collaboration with the other SOHO instruments were performed, also taking into account quiescent streamers and a few active ones.

During my weeks as lead observer two interesting events occurred. At the beginning of June 1996 UVCS caught the first CME in ultraviolet light in the outer corona. During the event peculiar mass motions consistent with untwisting magnetic fields around an erupting flux tube were observed, confirming a previous Skylab observation (Antonucci et al., 1997a). On May 1, 1997 the LASCO team, working in the office next door, spotted a sun-grazing comet in the large field of view of their coronagraphs. This was an unexpected opportunity and I proposed that the team try to observe the comet along its path across the solar corona. Guessing the speed of the comet we predicted its successive positions and by accordingly moving the UVCS slit we did indeed succeed in detecting the comet several times as it approached the limb of the solar disk in the plane of the sky. The comet was again spotted and traced along its path when it reemerged at the opposite solar limb. To be able to perform this observation in ultraviolet light was quite a rewarding experience, although the team involved in the comet chase had to stay at the EOF for many long hours.^45^ Upon a preliminary analysis the H i Ly$\alpha $ data looked very interesting; however, I did not pursue their study any further.

Taking advantage of the quiet Sun conditions, I proposed a sequence of supersynoptic, global Sun observations lasting two weeks, making it possible to observe the full outer corona at very high spatial and spectral resolution. The supersynoptic observation was first performed in August 1996, on the occasion of the Whole Sun Month campaign, which ran from August 10 to September 8, 1996. The same sequence was repeated several times in the following months, continuing as long as the large-scale configuration of the corona remained relatively stable.^46^ This program allowed a detailed study of the typical solar minimum configuration and dynamics of the wind in the solar corona.

### What We Learned with the UVCS Spectroscopic Observations of the Corona

As in the case of the XRP observations, the innovativeness of the instrument made it possible to observe new phenomena and as a consequence obtain unexpected results. When the first UVCS observations were performed, the observed profiles of the O vi lines were quite surprising. The widths of such lines, if simply interpreted as thermal broadenings, yielded temperatures up to two orders of magnitude in excess with respect to the expected coronal temperature of the order of 1 million K. The H i Ly$\alpha $ 1216 Å lines emitted by neutral hydrogen were found to be broadened to a lesser extent. A first quick analysis of the wind-velocity regimes in the corona – using the diagnostic technique based on the O vi 1032, 1037 Å doublet ratio to mark the boundary of the regions where the wind speed exceeded 100 km s^−1^ – showed that spectral lines were wider in the regions where the solar wind flows and the largest line widths were observed in the core of coronal holes (see, for example, Antonucci et al., 1997c). Further analyses confirmed that the observations unquestionably showed the existence of two phenomena: extreme kinetic temperatures deduced from the broadenings of the line profiles across the magnetic field, which were correlated with the wind speed; and a high degree of anisotropy in the kinetic temperatures – that is, quite different ion/atom velocity distributions across and along the magnetic field – suggesting preferential deposition of energy across the magnetic field.

These early findings implied two immediate corrections. On the one hand, when preparing an observation, there was the need to modify the masks that were applied to the spectra in order to isolate the spectral lines to be observed, by taking into account their real, unexpectedly large, width. On the other hand, there was the need to upgrade and adapt the Doppler-dimming codes to correctly interpret the data, since in the case of the anisotropy of the velocity distribution of the atoms/ions, during the resonant scattering process the incident photons would encounter in the corona absorbing profiles with different widths depending on the direction of incidence. Furthermore, while visiting the Institut d’Astrophysique Spatiale (IAS) in Paris, directed in those years by Alan Gabriel, Renato Martin, the first PhD student in solar physics at the Physics Institute in Turin, noticed that in addition to the C ii line considered by Noci, Kohl, and Withbroe (1987), a second C ii line present in the blue wing of the O vi 1037 line should be considered when computing the Doppler dimming of the O vi emission. This was an important point, since the presence of this line allowed us to extend the diagnostic capability of the O vi doublet to much higher velocity values exceeding 400 km s^−1^. Giancarlo Noci ensured that this point was taken into proper account in the codes developed in Dodero et al. (1998) and Li et al. (1998). Following in-depth analyses of the wind speed with the updated diagnostic codes, the velocity distribution of neutral hydrogen atoms and O vi ions was indeed confirmed to be anisotropic to the highest degree in the core of coronal holes where the fast wind originates (Kohl et al., 1997, 1998; Antonucci, 1998; Cranmer et al., 1999; Antonucci, Dodero, and Giordano, 2000).

Thanks to the UVCS data, it was now possible to observe the flow of the fast wind in the corona out to several solar radii during its acceleration process. In the core of coronal holes neutral hydrogen and protons were traced approximately out to 4 solar radii and the oxygen component out to 5 solar radii, where its flow speed approaches that of the heliospheric fast wind. The remarkable acceleration of the oxygen component was interpreted as the signature of energy dissipated by ion-cyclotron resonant Alfvén waves in the outer corona (Cranmer, Field, and Kohl, 1999) and energy was found to be dissipated at the maximum rate across the magnetic field at about 2.9 solar radii (Telloni, Antonucci, and Dodero, 2007), that is, beyond the sonic point, as expected on the basis of the solar-wind theory.

**Figure 16:**
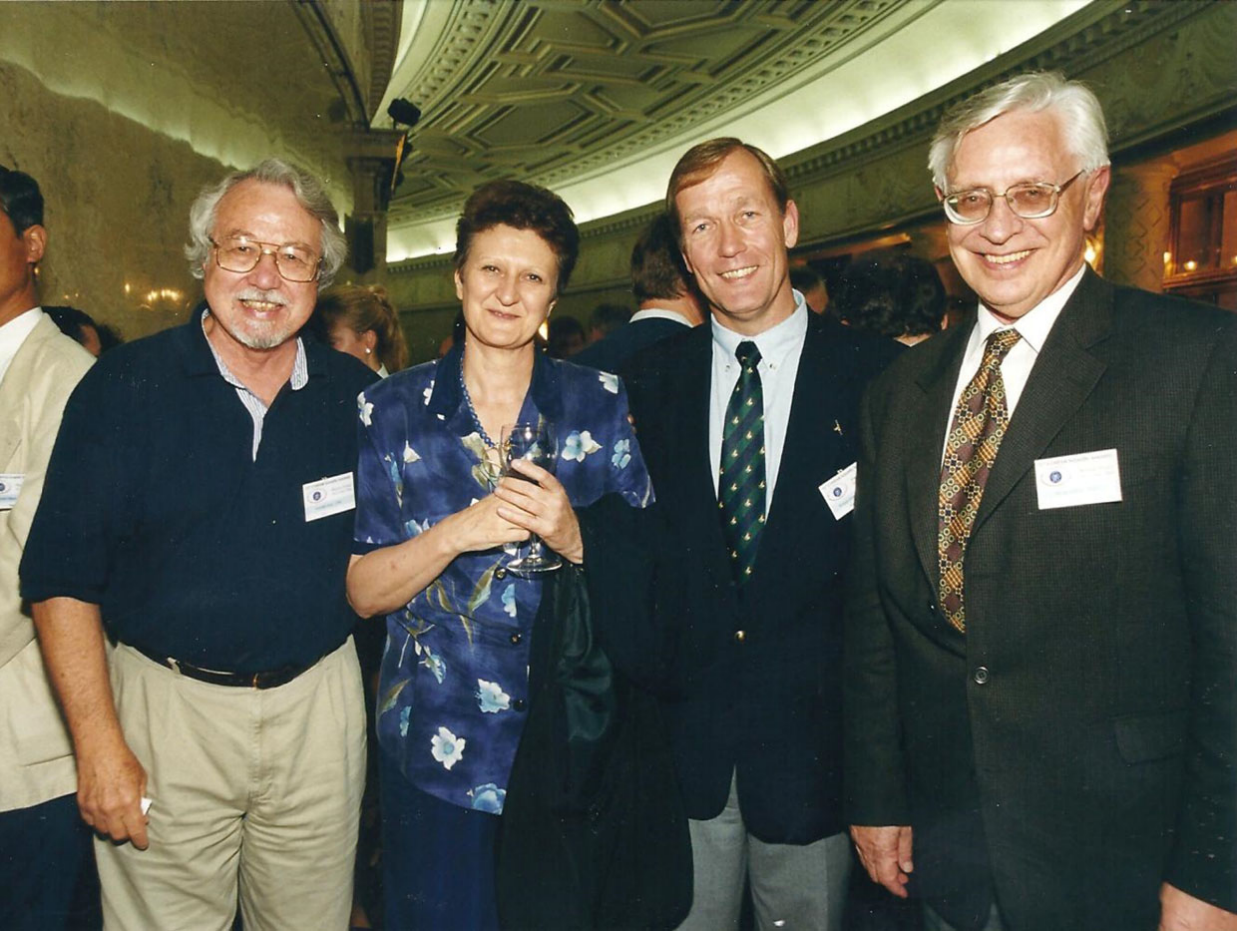
COSPAR Scientific Assembly in Warsaw, 2000. From the right: Ian W. Axford, Wim Hermsen, Ester Antonucci, and Norman F. Ness.

The dynamics of the corona was studied in detail from the fast-wind regime observed in the core of the solar-minimum polar holes to the slower wind close to the interface with the equatorial streamers. It was also possible to relate the wind speed to the flux-tube areal expansion. In particular, the slow wind emerging from the open magnetic-field lines characterized by nonmonotonic expansion – separating the substreamers present within the solar minimum equatorial streamers – was found to be related to variations in the abundance of oxygen, observed as a streamer core dimming in O vi (Noci et al., 1997). A wealth of results was achieved thanks to the UVCS observations of solar wind, streamers, coronal mass ejections, and comets, throughout Cycle 23. These results, presented at several international conferences (Figure 16), are in part reported in various review papers (e.g., Abbo et al., 2016; Cranmer, Gibson, and Riley, 2017; Antonucci et al., 2020a; and references therein). Many more results remain to be extracted from the UVCS data collected over one full solar cycle.

## Solar Orbiter, the Second European Solar Space Observatory and My Last Space Mission

### The Origin of the Solar Orbiter Idea

In 1993 when space solar physicists were still engaged in the payload integration and verification activities in view of an imminent SOHO launch, Per Maltby organized in Oslo a workshop sponsored by ESA on the ‘Scientific Requirements for Future Solar-Physics Space Missions’^47^ to explore new ideas for the next European solar observatory in space. Although it turned out to be an extremely long-lived mission, SOHO was designed for operations lasting two years, to be extended to six years in case of success, hence it was time for the community to start thinking of the future of European solar science from space. The next step in the solar exploration was going to be a mission characterized by a close approach to the Sun to investigate in detail its atmosphere from the layers below the photosphere, accessible via helioseismology, to the outer corona at all latitudes, including the solar poles and to navigate the unexplored circumsolar regions. However, the difficult path to achieve the approval for the next solar mission in the ESA science program lasted more than one solar cycle, and almost another whole solar cycle was needed before the new mission was finally flown.

The idea of placing telescopes aboard a solar orbiter emerged from a rich scenario of hypotheses and preliminary studies circulating in the 1990s among scientists and space agencies.^48^ The crucial event in the genesis of the next European observatory in space, Solar Orbiter, took place in March 1998, when ideas for future solar missions were discussed at the ESA conference ‘A Crossroads for European Solar and Heliospheric Physics’,^49^ organized in Tenerife by the ESA Solar Physics Planning Group chaired by Eric Priest and the Istituto de Astrofisica de Canaries. This event had been planned in view of the forthcoming ESA call to submit proposals for the two flexible missions F2 and F3, with launches expected with the time frame 2007 – 2009. The call for proposals was issued in the following year, on September 30, 1999, in the frame of the Horizon 2000 Plus Program,^50^ further extending to the year 2016 the planning of the ESA scientific program that had begun with Horizon 2000.

The outcome of the Tenerife conference was a consensus to recommend that ESA fly, as the next solar-heliospheric mission, an observatory in orbit around the Sun. In the session dedicated to the future missions, Eckart Marsch presented the concept of InterHelios (Marsch et al., 1998). The profile of this mission was similar to the one later adopted for Solar Orbiter, except for its orbit, which laid in the ecliptic plane. The key idea was to exploit the heliosynchronous segments of the orbit at heliocentric distances near 0.3 AU to study the characteristics of the near-Sun solar wind and identify its source regions with a combination of in-situ and remote-sensing instruments. In the same conference session, on behalf of a European team exploring a possible collaboration with NASA for the development of a Solar-Terrestrial Relations Observatory (STEREO) multispacecraft fleet, Bothmer et al. (1998) presented as one of the possible mission configurations a scenario including an out-of-ecliptic spacecraft. The inclusion of a polar element was inspired by the feasibility study of a Solar Polar Sail Mission carried out by Marcia Neugebauer and collaborators in 1998 at the Jet Propulsion Laboratory (JPL).^51^ The polar element was presented at the meeting as a plausible ESA contribution to the STEREO mission.

More than two decades earlier, in September 1974, I had attended the presentation of the European space programs during a conference at the European Space Research Institute (ESRIN) in Frascati.^52^ One of the most stimulating talks concerned the out-of-ecliptic mission jointly studied by Europe^53^ and NASA. The mission approved two years later consisted of two spacecraft envisioned as flying in formation out to Jupiter and then to head for the two opposite solar poles. Eventually, in 1990, only the ESA spacecraft, Ulysses, was flown. In the same 1974 meeting the idea of imaging the solar polar regions, was brought forth. To me, the perspective of exploring the poles of the Sun was quite fascinating and, after many years, the Tenerife forum turned out to be an excellent opportunity to explore this possibility by merging the study of the circumsolar regions and the observation of the poles put forward in the InterHelios and STEREO presentations, respectively. Thus, both during the discussions at the meeting and in private conversations, I tried to make the point that an InterHelios-type mission with an orbit sufficiently inclined with respect to the ecliptic plane to image the poles would have greatly enhanced the science objectives of the next European solar mission. During the session on future missions, in the heat of the argument I almost sat right down on the floor since I forgot that the movable seats in the congress room had to be pulled down horizontally before one could sit. This event was quite funny and had the ‘honor’ of being mentioned by Eric Priest in his speech during the closing dinner as the ‘trick of the disappearing lady’.

The concept of an ESA Solar Orbiter mission as further development of InterHelios was better substantiated and formulated in a preassessment study conducted by ESA in 1999 in order to evaluate the potential risks and technology issues related to the mission profile. The outcome of this study was the basis for the proposal we submitted on January 27, 2000 in response to the ESA call for the flexi-missions F2 and F3.

### The Solar Orbiter Odyssey

I spent the first decade of the new century being fully involved in the long odyssey of Solar Orbiter, participating in the proposal of the mission, in the related studies promoted by ESA and coordinating, with the full support of ASI, the Italian interests in this mission until the final approval. There was a great synergy within the European group of scientists, growing in number over time, who worked for the Solar Orbiter’s positive outcome, instrumental in overcoming the various obstacles.

The proposal ‘Solar Orbiter – High-Resolution Mission to the Sun and Inner Heliosphere’,^54^ coordinated by Eckart Marsch and Rainer Schwenn and submitted to ESA in 2000, envisioned a mission that had characteristics close to those of the mission actually flown in 2020. Our idea was to develop a near-Sun, out-of-ecliptic mission with perihelion below 0.21 AU and a maximum orbit inclination of 38°, consisting of a three-axis stabilized spacecraft constantly pointing at the Sun with a pseudosynchronous viewing ten days long. The strawman payload included a UV and visible-light (VL) coronagraph^55^ based on the UVCS diagnostic techniques. The proposed mission was selected a few months later by the ESA Science Program Committee, thus the future observation of the polar regions was ensured. The main remaining problem concerned its budget, which exceeded the typical budget for a flexible mission. As a mitigation, Solar Orbiter could take advantage of some of the technological developments required for the cornerstone Mercury mission, later renamed BepiColombo, in honor of Giuseppe Colombo of the University of Padua. Within the same timeframe, NASA’s Living with a Star initiative was included in the fiscal year 2001 presidential budget released on February 7, 2000, with program elements being a new space-weather network including a Solar Dynamics Observatory. The Solar Orbiter selection was immediately followed by a ‘delta’ assessment study.^56^ Yves Langevin of the IAS kindly assisted the Solar Orbiter study team on the aspects concerning the mission design and analysis. In 2004 the ESA Science Program Committee confirmed the selection of Solar Orbiter within the Horizon 2000+ Program, but this was not yet the definitive word; we had no idea what a long path we still had to face before the final approval.

Solar Orbiter was integrated into the new Cosmic Vision 2015 – 2025 ESA scientific program in 2008, with the consequence that it was placed back into competition with other missions. Following a second assessment study^57^ the mission was finally selected as a ‘candidate’ for the first medium-class mission, M1, of the Cosmic Vision Program in 2010, and after a further definition study,^58^ Solar Orbiter was definitively selected as the first element of Cosmic Vision 2015 – 2025 in 2011. NASA’s contribution, including the launcher and science instruments, was crucial for mitigating the budgetary constraints. The fact that the mission was actually approved two years after the selection of the scientific payload implied that the design studies of the instruments and the spacecraft were not perfectly phased in the first years.

**Figure 17:**
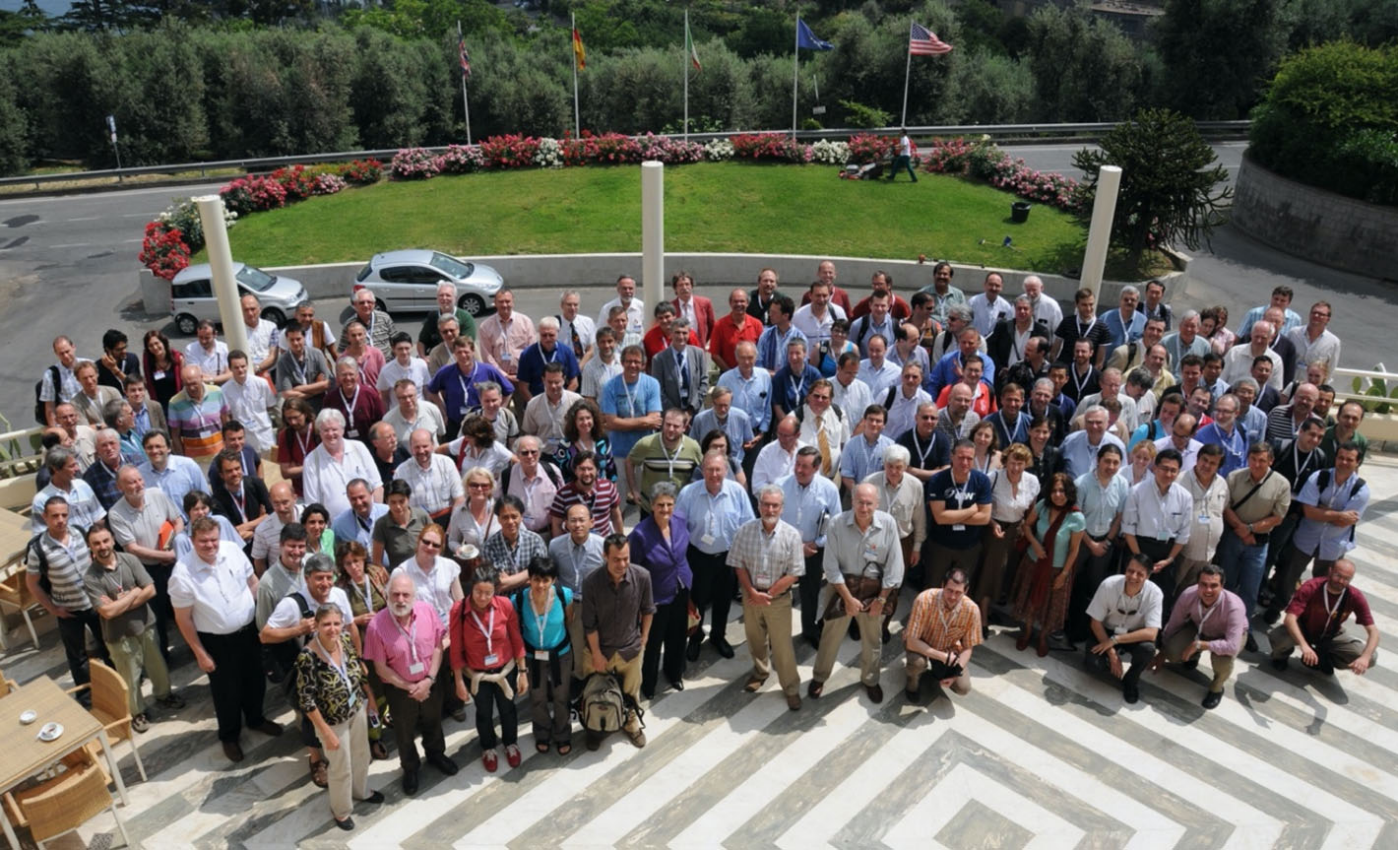
Third Solar Orbiter Workshop in Sorrento, May 24 – 29, 2009.

The formal selection of the Solar Orbiter instruments was announced in March 2009 when, as mentioned before, the mission was not yet fully blessed by ESA. This was of concern to the instrument principal investigators of the selected projects, and after a few telephone calls we decided to organize as soon as possible the third Solar Orbiter workshop in order to keep the attention of the agencies on our mission. I offered to organize the workshop at the end of May in Sorrento. Notwithstanding the fact that a few other international heliospheric meetings were held around that period and that the community was informed of the workshop at short notice, there was an unexpectedly large attendance, indicating once more that a wide community was interested in Solar Orbiter. At the meeting, there was ample time to present the mission and its payload and to discuss future plans and collaborations. Even the colleagues interested in participating in the ESA technological mission Proba 3, coordinated by Philippe Lamy of the Laboratoire d’Astrophysique de Marseille, were able to exchange ideas on the future proposal of a huge coronagraph constituted of two spacecraft flying in formation flight.^59^ The large attendance, the sun of Sorrento, the view of Vesuvius just in front of us, and perhaps also the good wine, gave us sufficient enthusiasm to face with optimism the future decisions of ESA (Figure 17).

### Future Solar Missions in the ESA Cosmic Vision Program

I had the opportunity to follow the vicissitudes of the Solar Orbiter in its transition phase from the Horizon 2000 to the next ESA scientific program in the role of one of the members of the ESA Space Science Advisory Committee (SSAC) in the years from 2004 to 2006. This was the period when the Committee was involved in the formulation of the program Cosmic Vision – Space Science for Europe 2015 – 2025.^60^ The new decade-long program envisioned a clear strategy for the theme ‘How does the Solar System Work?’, which included as a fundamental element for solar research the need to ‘chart the 3D magnetic field at the Sun’s visible surface using a Solar Polar Orbiter’. In the ESA plans this implied the development of a reliable solar-sailing system, and a preliminary study of the spacecraft for the polar mission based on such a technology was already underway at the European Space Research and Technology Centre (ESTEC) of ESA. At last, solar physics could take advantage of a mission primarily dedicated to the study of the polar regions of the Sun, foreseen to be observed at the minimum distance of 0.25 AU. Hence, one of the scientific objectives most dear to me was finding its optimal place in the ESA planning, thanks to the fact that Peter Cargill, also in the SSAC, and I shared the same view and the ESTEC engineers were interested in fulfilling the solar-sail technology. Later, the Cosmic Vision Program was superseded, and the present Voyage 2050 scientific plan identifies the solar polar science as one of the possible themes for medium missions, albeit with much less emphasis. My wish is that in the future the pre-eminent role assumed by ESA in space solar physics thanks to SOHO and Solar Orbiter will be maintained.

## Metis – the Multiwavelength Coronagraph for Solar Orbiter

When we were preparing the Solar Orbiter proposal, I was coordinating the Italian team working on the new concept of the Ultraviolet and Visible-light coronagraph envisioned for the mission. In addition to the historic nucleus comprising Giancarlo Noci and Giuseppe Tondello, this effort involved all our younger collaborators, who perfected their skills during the development, implementation, and operations of UVCS and, in turn, their students. The emphasis this time was on designing an instrument capable of simultaneously imaging the full solar corona in the resonantly scattered UV emission lines and in the polarized visible light, with the aim of measuring the plasma outflow speed with the same diagnostic techniques adopted for the UVCS data. The simultaneous imaging of the global corona at different wavelengths – obtained of course at the expense of the great spectroscopic capabilities that characterized UVCS – was preferred in order to be able to trace the propagation and evolution of the solar wind in the corona at high spatial and temporal resolution. The strawman payload described in the Solar Orbiter proposal included the design of a coronagraph imaging the EUV/UV emission of the He ii Ly$\alpha $ 304 Å, H i Ly$\alpha $ 1216 Å lines and measuring the polarized brightness of the visible K corona by using mirrors coated with ad hoc multilayers (Antonucci et al., 2000). The goal was to derive, on the basis of these data, global maps of the flow velocity of the two major components of the solar wind, the H and He components. Throughout the coronagraph development, the instrument design effort was mainly carried out by Silvano Fineschi, Giampiero Naletto, and Marco Romoli, whilst Daniele Spadaro was more involved in the definition of the scientific objectives and Vincenzo Andretta in the operation concept. Gianalfredo Nicolini joined the team at the time of the instrument proposal in 2007, and he was fully devoted to this project; his contribution and support were indispensable from then on.

### SCORE – the Solar Orbiter Coronagraph Prototype

The instrument envisioned for Solar Orbiter had never been tested before, and therefore in a meeting in Florence I proposed to fly a prototype on a rocket. Whilst exploring the suborbital flight opportunities, Russ Howard of the NRL suggested that I get in touch with Daniel Moses, who would shortly be attending the first Solar Orbiter workshop, ‘Solar Encounter’, held in Tenerife in May 2001.^61^ This event was a great opportunity to sort out how to proceed in order to fly the Solar Orbiter UV-VL coronagraph prototype within the NASA suborbital flight program. This was the first step of a long-term collaboration with Dan Moses, crucial for realizing the rocket project, which continued throughout the phase of design and development of the Solar Orbiter coronagraph.

The Helium Resonance Scattering in the Corona and Heliosphere sounding rocket (HERSCHEL),^62^ built to establish proof of concept for the Solar Orbiter coronagraph, was successfully launched on September 14, 2009 from the White Sands Missile range in New Mexico. The instrument package was composed of the multiwavelength Sounding-rocket Coronagraph Experiment (SCORE),^63^ the coronagraph prototype developed by the Solar Orbiter coronagraph team, the Helium Coronagraph (HECOR) developed by Frederic Auchère and collaborators of the IAS, and the HERSCHEL Extreme-ultraviolet Imaging Telescope (HEIT), instrument developed by Dan Moses and Jeff Newmark of the NRL. SCORE was designed to obtain simultaneous narrowband H i 1216 Å, He ii 304 Å and visible-light K-corona images from 1.5 R_⊙_ to 3.5 R_⊙_ (Fineschi et al., 2003; Romoli et al., 2007). HEIT observed the helium emission on the solar disk. It was much easier to develop the program and launch the instruments on the rocket than to write the HERSCHEL paper! The first results on the abundance of helium in the outer corona were obtained in a relatively short time, but because of the many commitments of the partners involved in this program, a full solar cycle elapsed from the rocket launch to the publication of the results in Nature Astronomy (Moses et al., 2020). The measurement of helium in the corona was obtained in correspondence with a period of anomalously quiet conditions during the solar minimum of Cycle 23 when the solar-wind speed in the heliosphere reached its lowest values.

### A Brief Participation in the Solar Dynamics Observatory with the Transition Region Spectroheliograph Project

Whilst the HERSCHEL proposal was taking shape, we briefly participated in the Solar Dynamics Observatory (SDO) mission studies. In August 2002 the Solar and Heliospheric Activity Research and Prediction Program (SHARPP) was selected by NASA for the phase-A study of the SDO mission. SHARPP was led by Russ Howard of the NRL, in collaboration with Pierre Rochus of the Centre Spatial de Liege, Belgium, and the Italian consortium led by the Observatory of Turin. SHARPP included a suite of EUV coronal imagers 64 and the visible-light coronagraph, KCOR. The Italian contribution to the EUV component of SHARPP consisted of the Spectroheliograph for the Transition Region (SPECTRE).^65^ The scientific objective of the SPECTRE experiment was to study the plasma in the transition region by obtaining high-resolution full-disk images of the solar atmosphere (1.2 arcsec) in the O v 629.7 Å line, emitted at about 2.5 × 10^5^ K. The requirements driving the optical design were satisfied by an innovative solution consisting of two spectrographs with opposite dispersion placed in tandem.

In September 2003, during the phase-A study, quite unexpectedly NASA decided not to proceed with the realization of the SHARPP suite of instruments, which was substituted with the EUV payload developed by the Lockheed Martin Solar and Astrophysics Laboratory (LMSAL). The work already done in the definition of SPECTRE’s optical design was reported in two papers by Naletto et al. (2004; 2005).

### The Metis Coronagraph

Several years elapsed between the preliminary studies of the UV coronagraph performed in view of the mission proposal, submitted at the beginning of 2000, and the solicitation of proposals for the Solar Orbiter instrumentation issued by ESA in September 2007. In the meantime, the coronagraph studies were progressing thanks to the Solar Orbiter Payload Accommodation – Heat Shield Study promoted by ESA. A letter of intent (LOI) to propose the Coronagraph for the Solar Orbiter Mission was submitted in September 2006 in response of the ESA Call for Submission of LOI, with a detailed description of the science objectives, the instrument design, and the management approach. In this phase the project was named Coronal Imaging Advanced Observations (CIAO).^66^ The aim of the ESA study, carried out from November 2006 to September 2007, was to address, among other matters, the definition of the interface of the remote-sensing instruments and the thick heat shield required to protect the platform of the instruments from the high flux of solar energy and thus mitigate the harsh thermal conditions expected when facing the Sun at perihelion. Never before had solar telescopes been designed to face the Sun at such a short distance. In particular, this was a study of crucial importance for the coronagraph, the only remote-sensing instrument protruding outside the feed-through of the shield protecting the spacecraft.

**Figure 18:**
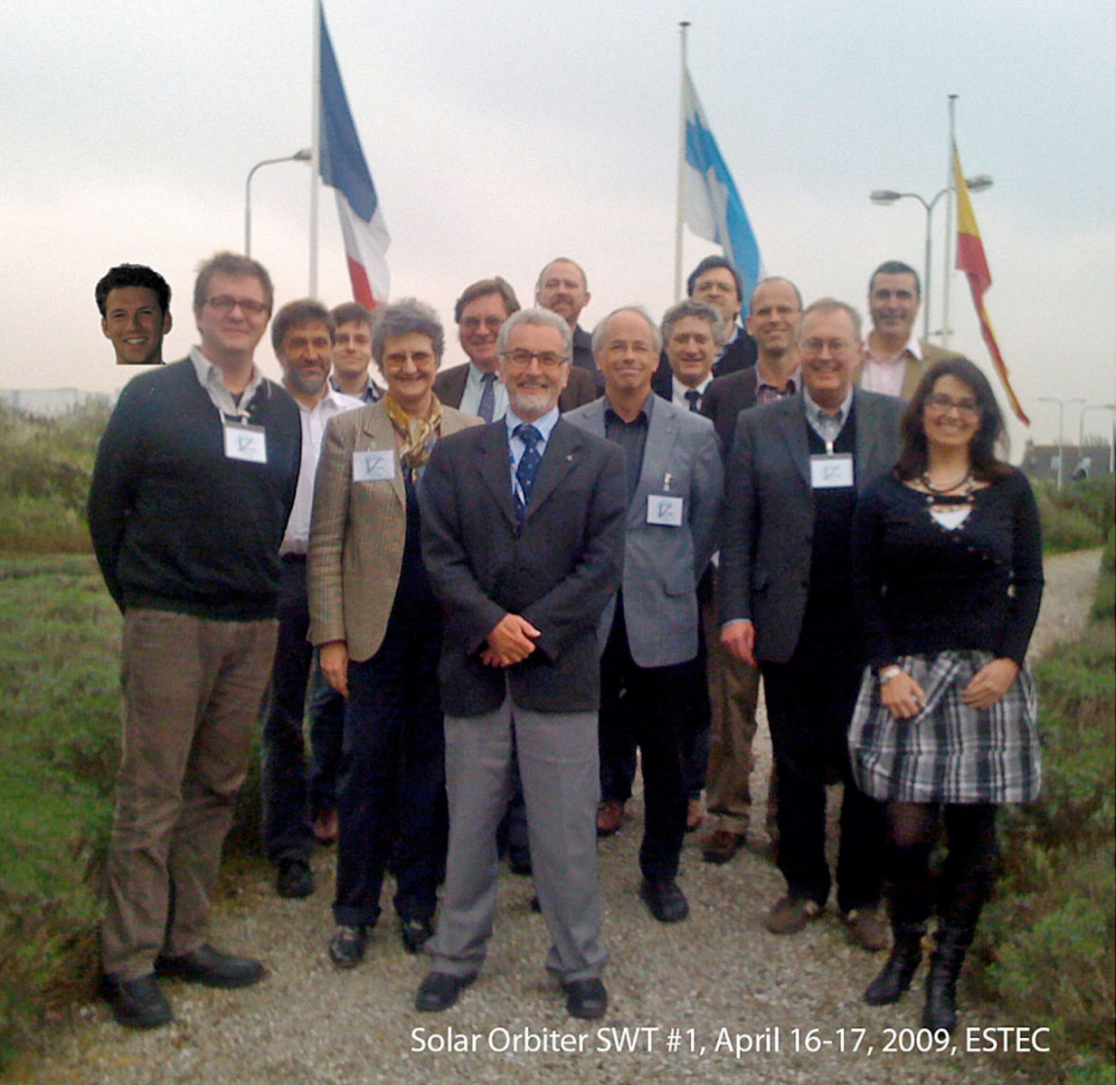
First Solar Orbiter Science Working Team meeting at ESTEC in April 2009. From the left, first row: Stuart Bale, Joachim Woch, Ester Antonucci, Richard Marsden (Solar Orbiter Project Scientist), Arnold Benz, Eckart Marsch, and Danielle Renton (Solar Orbiter Instrument System Engineer); second row: a ‘virtual’ Daniel Mueller (at present Solar Orbiter Project Scientist), Timothy Horbury, Pierre Rochus, Christopher Owen, Philippe Klentzkine (Solar Orbiter Project Manager), Milan Maksimovic, Robert Wimmer-Schweingruber, and Havier Rodriguez-Pacheco.

By the time of the final proposal submitted on January 15, 2008 the design of our instrument had evolved and become more and more ambitious. We indeed proposed a complex instrument composed of two elements, a EUV-UV coronal and disk spectrometer and a multiband visible/EUV/UV coronagraph, named ‘Multi Element Telescope for Imaging and Spectroscopy’ (METIS, the acronym coinciding with the name of the oceanid nymph, mother of wisdom and deep thought).^67^ However, the recommendation of the payload review committee, we received in July 2008, was to drastically descope METIS to a coronagraph-only experiment. Although the spectrometer was not approved, we were in any case pleased that the METIS coronagraph was going to be onboard such a challenging mission. The UV spectrometer for Solar Orbiter was later included in the payload as an ESA-provided instrument. At this point METIS was redesigned with the aim of imaging the H, He, and polarized VL coronal emission, while still maintaining some EUV spectroscopic capabilities, though limited to a coronal sector 35° wide. The payload selection was formally announced in March 2009; at the same time, I was appointed by ESA to be the Principal Investigator of the METIS project. In April 2009 the first Solar Orbiter Science Working Team meeting was held at ESTEC (Figure 18).

The need to solve instrument-accommodation problems and minimize the total energy transmitted into the instrument by minimizing its entrance aperture led the team to invent a new type of coronagraph. The new design, the result of a collaborative effort, was based on an inverted-occultation concept proposed by Dan Moses and turned into reality by the METIS team, which developed this idea and designed a brand-new coronagraph – first by Silvano Fineschi who was thinking of a similar approach but starting from the UVCS configuration, and then by Marco Romoli, who verified the feasibility of this concept. The refined design of the new coronagraph, with visible-light and EUV/UV paths, appeared in June 2009 in a preliminary technical report^68^ drafted in preparation for the Experiment Interface Document for the Instrument Design Status Review, in November 2009. The new design was first presented at the International Conference on Space Optics by G. Naletto et al. in 2010 (the paper was published as SPIE proceedings several years later, Naletto et al., 2019).

To make a long story short, at the end of phase B in 2012, the European and Italian space agencies asked us to consider the fact that the impact of the METIS repointing mechanism was adding complexity both at the instrument and spacecraft level. In addition, they asked us to reduce the instrument mass and the cost for developing the coronagraph. Difficult decisions had to be made; the only solution was a drastic descoping of the instrument. The contribution of the UV-detector assembly on the part of Sami Solanki of the Max Planck Institute, and a letter of support of the METIS advisory board, including prominent international colleagues, written in January 2013, certainly helped in the negotiations with ASI, which in the end approved a simplified version of the coronagraph. In the descoping process, METIS lost the He-imaging capability and the sector dedicated to spectroscopy, as well as the repointing mechanism. The phase-B structural design had included this mechanism to ensure that the coronagraph would constantly point at the Sun’s center, even when other remote-sensing instruments would be required to point at the selected target off-center and at the limb of the Sun. The loss of this mechanism meant that the entrance door of the instrument had to be closed each time Solar Orbiter pointed off-center when observing near perihelion, that is, when the highest spatial resolution in coronal imaging can be obtained. This was the price to pay. Thus, METIS became simply *Metis*, that is, the instrument’s name could no longer be an acronym, having lost the spectroscopy element. Notwithstanding the painful descoping, the coronal imaging in both the ultraviolet H i Lyman alpha and the polarized visible-light channels were maintained. Hence, the main goal of obtaining for the first time instantaneous global maps of the flow of the principal component, that is, the hydrogen component, of the solar wind in the corona was preserved. The descoping phase ended with the signature of the new industrial contract with a consortium formed by OHB Italia in Milan and Thales Alenia Space Italia in Turin, at the time of the kickoff of phase C/D. The simplified design and the new industrial organization were presented at the third Metis scientific meeting, organized by Vincenzo Andretta at the Astronomical Observatory of Naples in October 2013.

**Figure 19:**
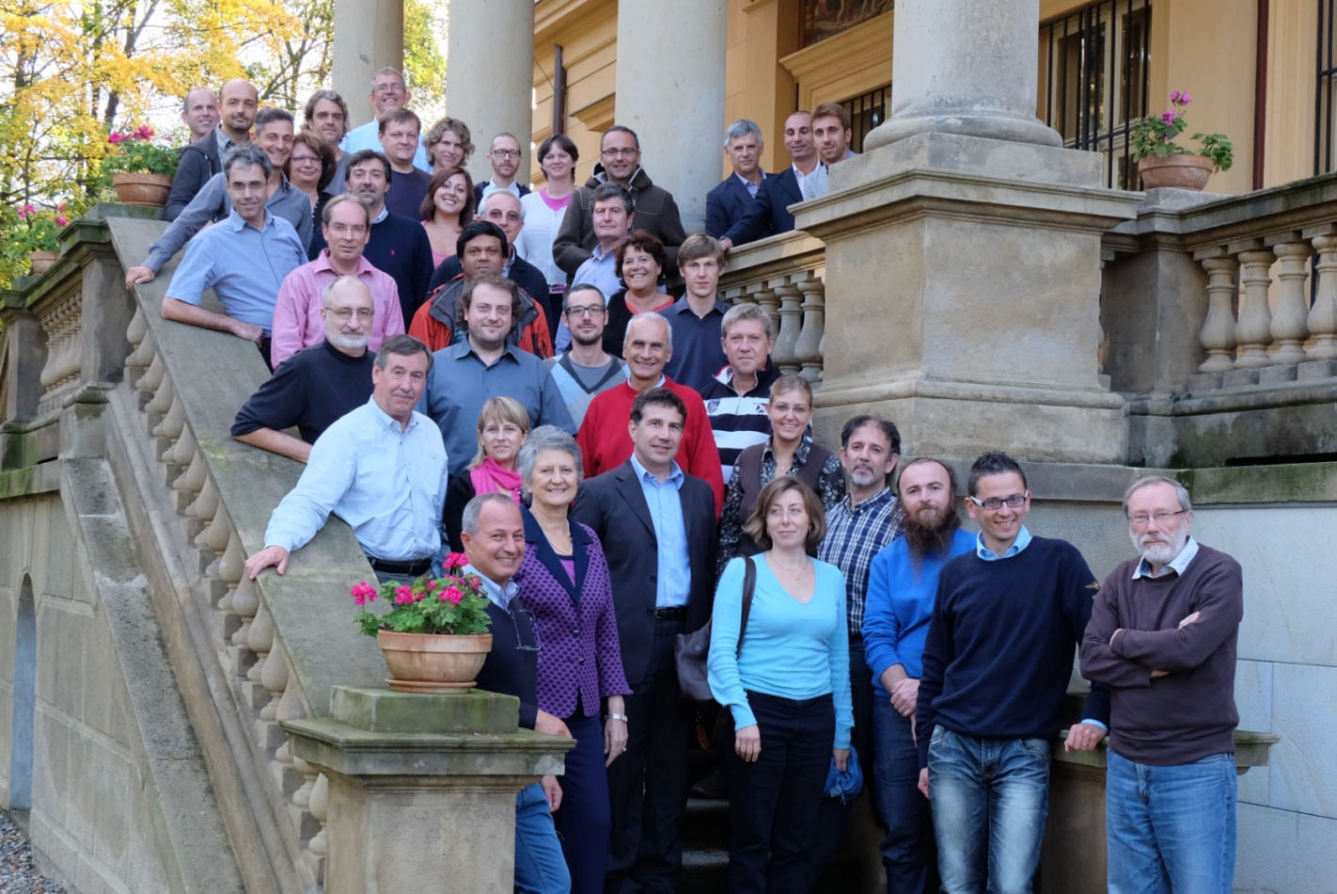
IV Metis Science Meeting in October 2014 in Prague at the Academy of Sciences, Vila Lanna.

**Figure 20:**
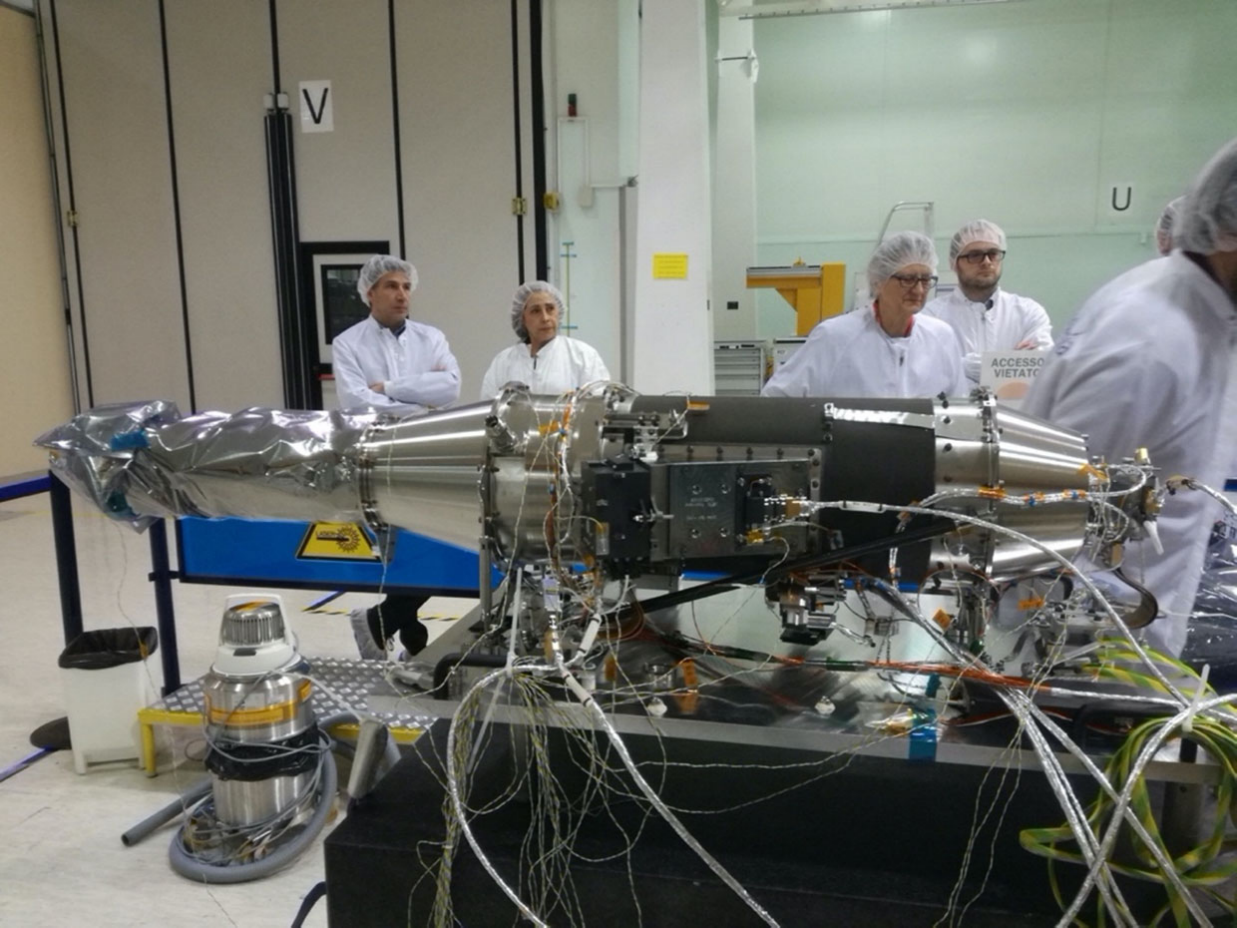
Metis prepared for the thermal tests at Thales Alenia Space, Rome, February 2017. From the left: Marco Castronuovo (ASI Metis Program Manager), Barbara Negri (ASI Scientific Programs Responsible), and Ester Antonucci.

**Figure 21:**
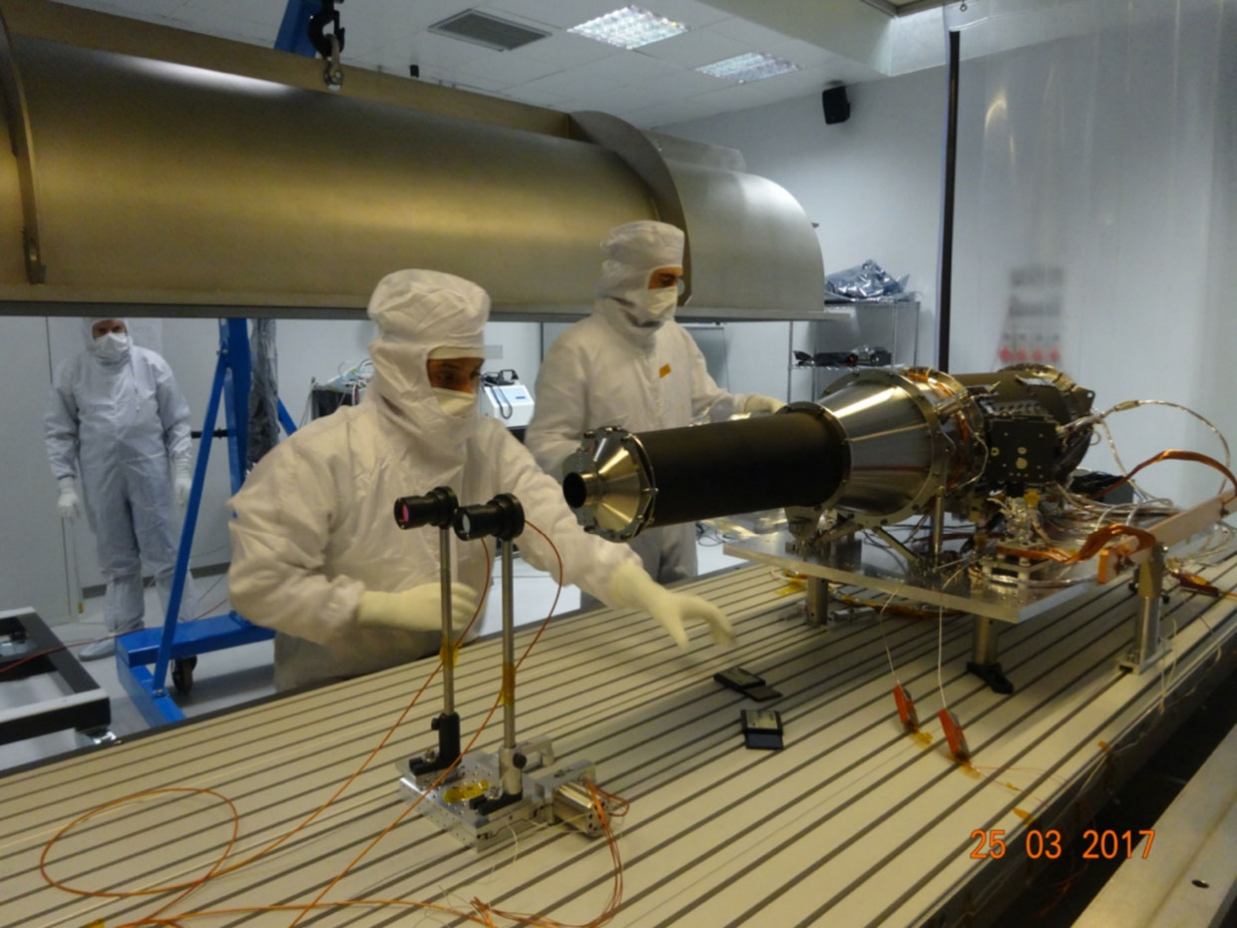
Metis during the calibration activities at the OPSYS facility at ALTEC, in Turin, March 2017 (picture taken by Silvano Fineschi).

**Figure 22:**
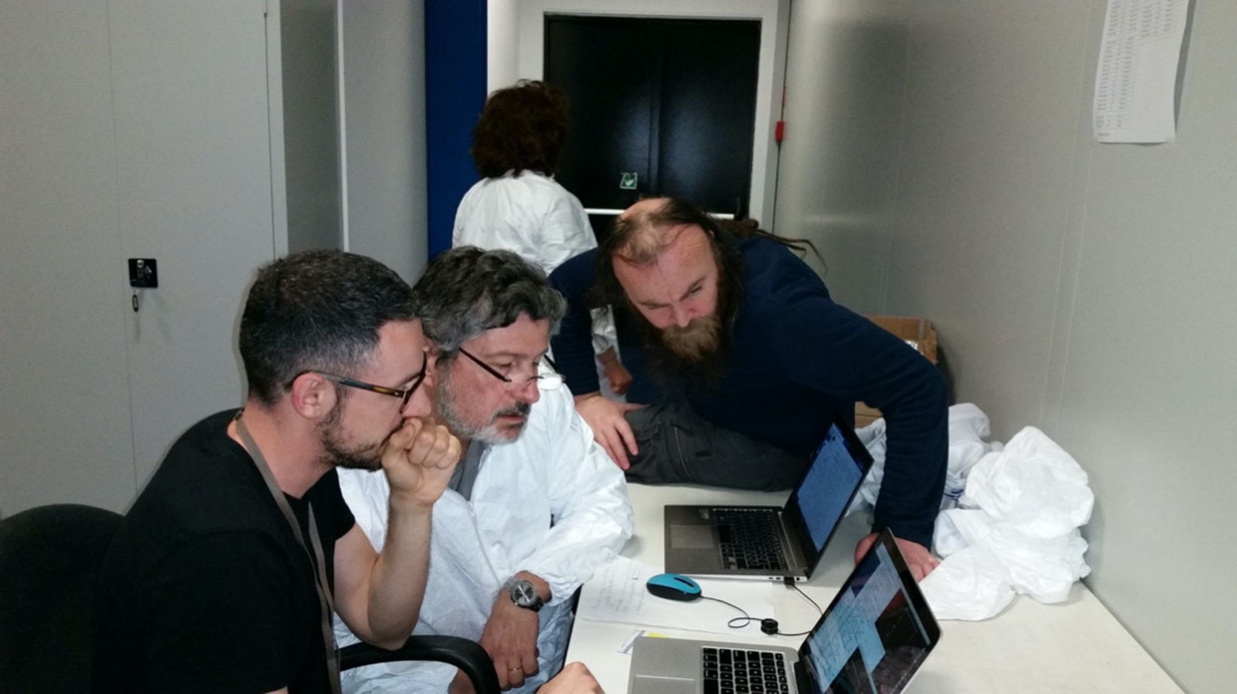
Metis calibration phase at the OPSYS facility, ALTEC, May 2017. From the left: Roberto Susino, Silvano Fineschi, and Gerardo Capobianco (picture taken by Ester Antonucci).

**Figure 23:**
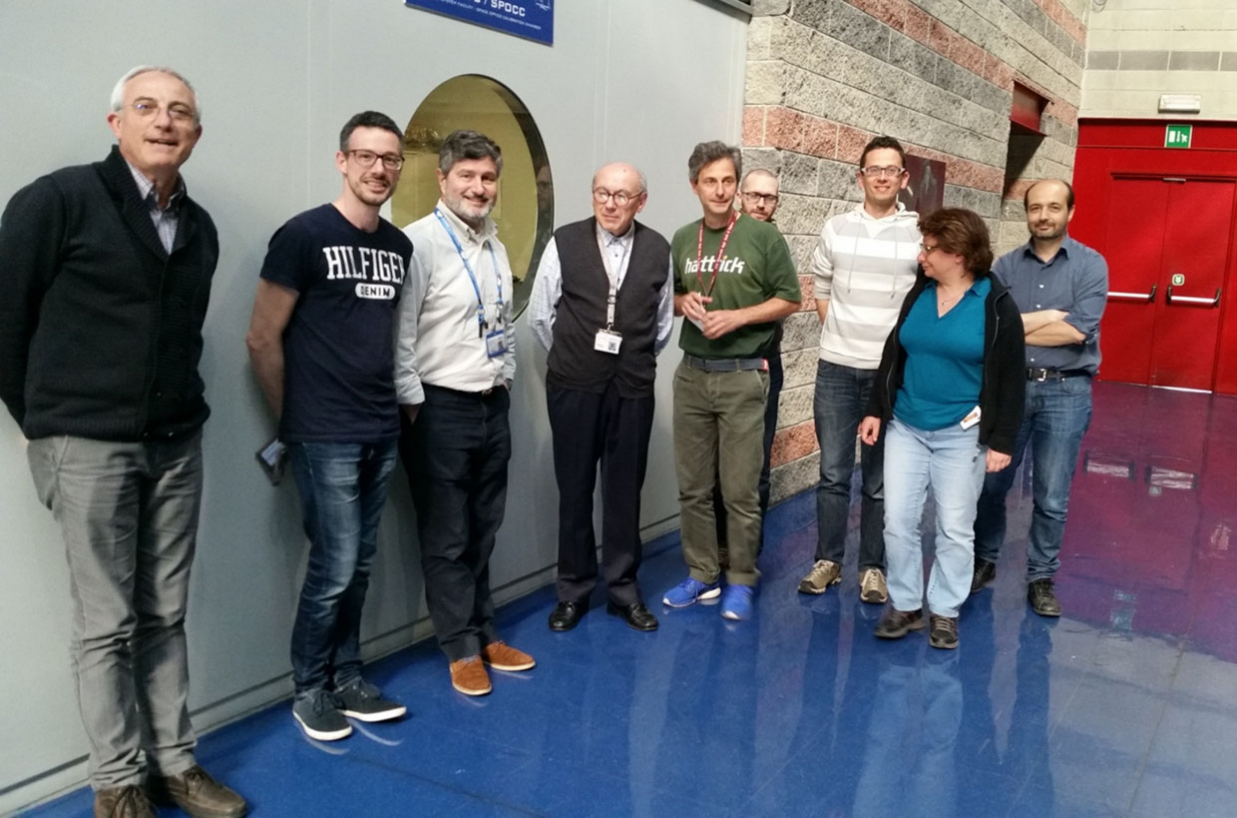
At the end of the Metis calibration phase at the OPSYS facility, ALTEC, May 2017. From the left: Daniele Spadaro, Roberto Susino, Silvano Fineschi, Giuseppe Massone, Gianalfredo Nicolini, Fabio Frassetto, Maurizio Pancrazzi, Michela Uslenghi, and Vincenzo Andretta (picture taken by Ester Antonucci).

The following years were extremely demanding but we were able to recover the time lost in the process of descoping and assigning the new industrial contract and to deliver the instrument on time. Many crucial decisions had to be made to ensure the optimal future performance of the instrument while respecting the time schedule, and many difficulties had to be overcome. All this was done in close contact with the industries involved in the fabrication of the instrument and with our partners manufacturing the mirrors, provided by Petr Heinzel of the Astronomical Institute of the Czech Academy of Sciences (Figure 19), and the detectors provided by the Max Planck Institute, with the full support of Filippo Marliani, ESA Solar Orbiter Payload Engineer, and the ASI managers Barbara Negri and Marco Castronuovo (Figure 20). In parallel with the development of SCORE and Metis we set up the OPSYS, a facility of the Astronomical Observatory of Turin (OATo) located on the premises of the Aerospace Logistics Technology Engineering Company (ALTEC S.p.A) in Turin.^69^ This facility was used to integrate and calibrate Metis (Figures 21, 22, 23).

### The Space-Weather KuaFu Mission

A white-light and H i Ly$\alpha $ 1216 Å coronagraph to obtain double-band images of the corona was also proposed for the Chinese KuaFu mission, envisioned to explore the space-weather phenomena by means of one spacecraft at L1 for Sun monitoring and two spacecraft orbiting the Earth to detect the magnetospheric effects of solar activity. In 2006, the Chinese National Space Administration (CNSA) authorized the prestudy of the mission led by Chuanyi Tu of Peking University. Considering this project to be a great opportunity for space-weather studies I proposed to provide the Ly-alpha Coronagraph (LyCo), to be developed in collaboration with Weiqun Gan of the Purple Mountain Observatory.^70^ Between 2006 and 2010 I attended four international symposia and workshops on the KuaFu project organized in China to discuss the mission scenario, the model payload and the international collaborations.^71^ The KuaFu project provided a great opportunity to discuss the solar and heliospheric science as well as visit beautiful sites, such as Sanya on the Hainan Island and Kunming in Yunnan, but it never became a reality.

## At the Astronomical Observatory of Turin

By the time of the Metis proposal, I had been appointed director of the Astronomical Observatory of Turin, now named the Astrophysical Observatory of Turin (OATo). Applying for a position at the Observatory as Senior Astronomer^72^ meant I could finally access, in 1995, the highest level of the academic/scientific profession. Whilst at the University it was extremely difficult to open new positions for young researchers, at the Observatory at last I had the possibility to gradually form a team dedicated in principle to solar-physics science and space projects. The projects dedicated to space pursued at the Observatory were the participation in the ESA mission Gaia, coordinated by Mario Lattanzi, and the participation in Solar Orbiter. In the role of astronomer, I continued to advise graduate students in their research work and for a few years I taught solar physics at the university. My last student was Daniele Telloni, who graduated in 2008. From the beginning he devoted his research to the solar wind both in the corona and in the heliosphere and in 2021 he published the first paper on the Parker Solar Probe – Metis Solar Orbiter joint science (Telloni et al., 2021).

### The International Heliophysical Year

During my five years as director of the Observatory, from 2005 to 2010, many exciting events and celebrations took place. The year 2007 was dedicated to the International Heliophysical Year (IHY) program sponsored by the United Nations. Our most important initiative that year was the organization in Turin of the Second European General Assembly of the IHY, June 18 – 22. It was a pleasure to host in Turin colleagues coming from countries not often represented at solar-physics meetings. Among many other initiatives in the frame of IHY, OATo also organized a program for high schools in North-West Italy, aimed at setting up a temporary space-weather network thanks to the distribution of a number of instruments for establishing small stations for space-weather forecasting run by students.

In the same year OATo and the Archivio di Stato di Torino,^73^ organized an exhibition, ‘*Nel Fuoco del Sole’* with the aim of displaying all the numerous documents relative to the history of the Astronomical Observatory. This occurred from September 25 to October 14, 2007.

### 250 Years of Astronomy in Turin

2009 was another special year marked by two important events: the celebrations of 250 years of astronomy in Turin and participation in the International Year of Astronomy, with a dense program of events and exhibitions.

According to tradition, astronomical studies in Turin date back to the first measurements of the Gradus Taurinensis, the degree of the meridian arc in Piedmont, by Giovanni Battista Beccaria in 1759. The scientific and cultural environment in the eighteenth century in Turin was very lively, enlightened by scholars such as Jean-Louis Lagrange, the famous mathematician and one of the founders of Turin’s Accademia delle Scienze, who spent the last part of his life in Paris. Lagrange attended the lectures of experimental physics held by Beccaria, who dedicated most of his studies to electricity. Beccaria’s interest in astronomy did initiate, under invitation of Charles Emmanuel III, Duke of Savoy and King of Sardinia, with the measurement of the meridian arc and the construction of a telescope, positioned in the royal gardens. The construction of the telescope was stimulated by the interest in astronomy aroused by the passage of the Halley comet. Although they never met, Beccaria was in touch with Benjamin Franklin for 30 years. Exchanging novel ideas on electricity and inventions, they corresponded first in Latin then each one in his own language. Thanks to Franklin one of the books on electricity written by Beccaria was translated into English and published in London. I suddenly became aware that the history of the Observatory was quite intriguing, thus to honor my predecessors I decided to restore the historic instruments, most of them still present in the Observatory, and to involve the astronomers interested in history in narrating episodes from the 250 years of astronomy in Turin. Their contributions were published in a book, *Osservar le stelle – 250 anni di Astronomia a Torino*,^74^ which also included the catalog of the restored instruments. Many enjoyable stories came to light during this search for the roots of the Observatory.

The historic instruments were displayed from October 1 to November 14, 2009 at Palazzo Bricherasio and Palazzo Lascaris^75^ in an exhibition with the same title as the book about the history of the Observatory. Laurent Levi-Strauss, representing UNESCO, was our guest during the inauguration of the exhibition. In 2009 Palazzo Bricherasio also hosted an exhibition dedicated to Akhenaton,^76^ the pharaoh who worshiped the Sun, with a contribution by part of the Observatory.

### The International Year of Astronomy

During the International Year of Astronomy, declared to celebrate the 400th anniversary of the first recorded astronomical observation by Galileo Galilei, Turin was the site of several other events that involved OATo. One of these was the ‘Torino Cosmology Colloquium: Latest News from the Universe’ of the Ecole Internationale Daniel Chalonge,^77^ held in October with the participation of the 2006 Physics Nobel Prize laureate George Smoot. Another was the day organized at the University by the Centro Unesco Torino to honor also the work of women astronomers, from Hypatia to Caroline Herschel. The rich program of events of 2009 took shape during conversations among friends and represented a nice parenthesis indeed between my daily duties as director of the Observatory and my involvement in Solar Orbiter. Lucia Abbo was of great help in the organization of the 2007 and 2009 conferences and events, managing to do this while not compromising her dedication to science. Her support was also crucial in the organization of the Third Solar Orbiter workshop in Sorrento.

I would also like to mention that during the International Year of Astronomy it was a great honor for me to be one of the astronomers invited to be received by Pope Ratzinger, Benedict XVI. We were guided by the Vatican astronomers on a very special visit of the Vatican City, the Accademia Pontificia, and Castel Gandolfo, the beautiful home of their headquarters close to Rome. 400 years after his first astronomical observations, Galileo was ‘definitely’ exonerated.^78^

**Figure 24:**
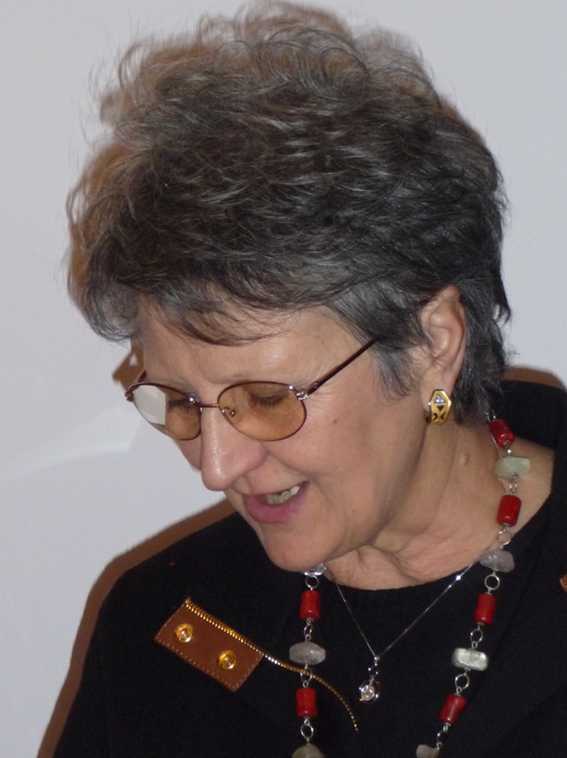
Astronomical Observatory of Turin, March 30, 2010. On the occasion of my retirement, looking at the presents and souvenirs and wearing two of them around my neck.

On the occasion of my ‘formal’ retirement in 2010 (Figure 24), when I was 65 years old, I received a very welcome and unexpected present. The planetologists of the Observatory proposed to assign my name to the minor planet ‘Esterantonucci’ – 1998 TB34 (22744). One of the motivations was: ‘…director of the Osservatorio Astronomico di Torino – the first woman to hold this position’.^79^

## Solar Orbiter on Its Way Toward the Sun

After I formally retired in 2010, when Solar Orbiter was not yet definitely approved and Metis still faced a long and sometimes difficult path, my time was almost exclusively devoted to the Metis project, until the delivery of the instrument to ESA. From 2013 to 2019, I also served on the European Space Sciences Committee, advising on solar physics and space-weather initiatives. After a while, my committee colleagues were erroneously convinced that I was a space-weather scientist since I was insisting that Europe should have a space-weather program as robust as, for instance, the Sentinels program for the observations of the Earth.

Once the Metis instrument was finally delivered in 2017, I resigned as principal investigator and Marco Romoli was appointed to lead the Metis activities. Although ASI kindly invited me to remain in that a role as long as possible, I deemed this to be the right moment to pass the responsibility to a younger person so that my successor would have sufficient time to get acquainted with his new role before the Orbiter launch. Moreover, he could rely on my advice, if needed, during the phase preceding the launch of Metis for the various tests and integration issues that were strictly related to my earlier activities and decisions. The Metis instrument, as delivered to ESA in mid-2017, is described in detail in the paper by Antonucci et al. (2020b) included in the special issue of *Astronomy and Astrophysics* dedicated to the Solar Orbiter mission (Müller et al., 2020).

Solar Orbiter was launched on February 10, 2020 just when the world was hit by the twenty-first century COVID-19 pandemic. This was the first mission – moreover, one of great complexity – to be operated almost from the beginning in the unforeseen anomalous conditions dictated by the recurrent lockdowns, that made it impossible to reach the usual work locations. I was absolutely impressed by the ability and the commitment of all scientists and engineers involved in the effort of operating the spacecraft and the payload in this totally new way, efforts that assured a fully successful commissioning and transition to the nominal mission.

Notwithstanding the difficulties during commissioning due to the COVID-19 pandemic, Solar Orbiter has already provided quite a rich harvest of scientific results, although the most important phases of the mission have yet to be reached. On May 15, 2020 Metis observed the solar corona for the first time and I was pleased to take part in the analysis and interpretation of Metis’s first light (Romoli et al., 2021). The first map of the solar wind in the corona was finally obtained.

## Conclusions

I have attempted to present my scientific contributions to solar physics over the course of half a century in the context of the development of space missions for solar physics and of the knowledge of the Sun and the heliosphere that we had at a given time and within the frame of the new information provided by the major solar space observatories flown from 1980 to the time of this writing. I have dealt in greater detail with the period preceding the SOHO launch, with the intent to illustrate the history and evolution through the years of our understanding of some of the physical processes and phenomena occurring in the solar atmosphere. With regards to SOHO and Solar Orbiter, I have tried to outline the history of these missions, as seen from my point of view and on the basis of my experience.

## Footnotes

1 Traditionally, Italian students were able to choose between two kinds of upper secondary schools: the classical lyceum, with an emphasis on the humanities, and the scientific lyceum, with an emphasis on the sciences.

2 The Italian Laurea was awarded upon completion of four to six years of university courses and the successful defense of a thesis.

3 This is a hill across the river Po, very close to the center of Turin, site of a beautiful church and crossed by many old underground tunnels used in the past for defense and as shelters.

4 The instruments of Mariner 2, launched in 1962, provided the decisive proof of the existence of the solar wind.

5 These results were reported in the theses successfully defended for the Laurea in Physics, Università degli Studi di Torino, on December 18, 1967 and for the Diploma di Specializzazione in Cosmic Physics, Università degli Studi di Torino, on March 25, 1972, entitled ‘Sull’effetto semidiurno a 70 m.a.e.’ and ‘Anisotropia Diurna Solare della Radiazione Cosmica’, respectively.

6 The lower-energy cosmic rays detected at ground level were also affected by the central meridian passage of the active regions, but preferentially when flares occurred close to the passage. In addition, the flux depression of high-energy particles was more extended, denoting a larger volume of the interplanetary medium involved in the solar modulation.

7 Special focus of the Physics Institutes was on the experiments with the Stanford Linear Accelerator, and the radio-astronomy observations obtained with the big dish in the Stanford foothills.

8 The Fraunhofer Institute, founded by Otto Kiepenheuer in 1943, was renamed as the Kiepenheuer Institute for Solar Physics after his death and at present is named the Leibniz Institute for Solar Physics.

9 I was appointed Professore Incaricato and Professore Associato in Physics in 1975 and 1983, respectively. At the same time, I was Assistente Ordinario from 1972 to 1983.

10 John A. Simpson of the Enrico Fermi Institute, University of Chicago, was kind enough to provide the Climax neutron monitor records.

11 Giovanni Perona, professor of Electromagnetic Fields at the Politecnico di Torino, earned a PhD degree as a student of Ian Axford at Cornell University, the reason why he was also interested in pursuing with us this cosmic-ray study.

12 Sunspot data were provided by Mario G. Fracastoro, who later became director at the Astronomical Observatory of Turin.

13 The satellite circled the Earth about every 96 minutes, with an eclipse period of approximately 30 minutes during each orbit (see ‘Solar Maximum Mission Flare Workshop Proceedings’, Editors: Kundu and Woodgate, 1986).

14 ATM was the first manned space observatory and carried eight instruments, each of which was the most advanced instrument of its type ever flown, covering the wavelength range from 7000 to 2 Å. The hot corona was imaged in the range from 2 to 60 Å with the X-ray telescope, one of the most successful Skylab instruments. The ATM scientific experiment payload weighed 900 kg.

15 Documents were typed by the indispensable and always very supportive secretaries and the fastest means to transmit papers and documents was the postal service; short messages were sent by telex.

16 Only the experiments detecting the electromagnetic spectrum above 20 keV and the instrument measuring the solar irradiance were not affected by this decision since they were integrating the signal over the full Sun.

17 The NOAA representative was David M. Speich.

18 M8: energy released during the flare equal to 8 × 10^−3^ erg cm^−2^; X1: energy released equal to 10^−2^ erg cm^−2^.

20 The two young ladies were supporting the preparation of the commands to be uploaded. Unfortunately, it was not possible to identify the young person between Ken Phillips and John Leibacher.

19 I was appointed XRP Deputy Principal Investigator at the beginning of August 1980.

21 Temporal resolution typically used: 1 s for BCS.

22 Including the associated Li-like Fe xxiv and Be-like Fe xxiii lines.

23 The numerous dielectronic recombination transition lines are completely unresolvable for quantum numbers 3<n<$\infty $ and the inner-shell excitation transitions are negligible for n>2.

24 Submitted to Solar Physics on April 10, 1981.

25 The resonance lines were well resolved since the flare occurred at 4 arcmin from the pointing direction.

26 See the chapter on ‘Flare Dynamics’ by E. Antonucci, D. Alexander, J.L. Culhane, C. de Jager, P. MacNeice, B.V. Somov, and D.M. Zarro.

27 See also Antonucci, 1989, with regard to the XRP results.

28 The industrial part of the phase-A study was conducted from July 1984 to October 1985, with final presentation in November 1985. The rider study to the phase-A study was performed between July and October 1985.

29 SOHO Solar and Heliospheric Observatory – Report on the Phase A Study, *ESA-SCI* (85)7, December 1985.

30 Roger Bonnet stepped down from this position in 2001.

31 President Ronald Reagan declared support of the international Solar Terrestrial Program on February 10, 1986.

32 See ‘The SOHO Mission – Scientific and Technical Aspects of the Instruments’ *ESA SP*-1104, Scientific Coordinator V. Domingo, November 1988.

33 Soon after the payload selection, I became part of the working group set up by ESA to study the ground segment and the operation scenario of SOHO. ESA was evaluating the possibility of developing a remote operation center in Europe, the European Science Data and Operations Centre (ESDOC); several countries, in particular France, Italy, and Spain, declared their interest and submitted a proposal to develop such a center. In the end only the Experiment Operation Facility, with the purpose of coordinating the observations and operating the payload instruments, was implemented at the GSFC, although scientific planning for limited periods was also carried on at MEDOC, at the Institut d’Astrophysique Spatiale (IAS) in Paris. In 1995 ESA granted to IAS, the University of Turin and RAL the right to host a SOHO data archive, the principal one being located at GSFC.

34 The detection of the location, orbital velocity, and spin rate of the SOHO spacecraft after its loss of control in 1998 was achieved with a bistatic radar configuration that utilized the Arecibo antenna as the transmitter and the 34-meter NASA Deep Space Network antenna at Goldstone (California) as the receiver.

35 ‘Promoting Research and Observations of Space Weather to Improve the Forecasting of Tomorrow’ Act: ‘In order to sustain current space-based observational capabilities, NASA shall: in cooperation with the European Space Agency, maintain operations of the Solar and Heliospheric Observatory/Large Angle and Spectrometric Coronagraph (SOHO/LASCO) for as long as it continues to deliver quality observations, prioritize the reception of LASCO data.’ https://www.congress.gov/bill/116th-congress/senate-bill/881/all-info.

36 At that time Giuseppe Vaiana, who had returned to Palermo after a long and successful career in the US as the leading scientist of the Soft X-ray telescope S-054 on Skylab, was the most experienced Italian solar scientist in space instrumentation.

37 Aeritalia, subsequently Alenia Space in 1990, and today Thales-Alenia Space – Italia.

38 Today Leonardo s.p.a.

39 The ASI was established in 1988 in place of the Space Office of CNR.

40 The selection letter arrived at CFA on March 11, 1988.

41 SAX was the first ASI mission devoted to astrophysics, for the study of the astronomical X-ray sources, launched on April 30, 1996, almost thirty years after the first Italian satellite ‘San Marco1’ was launched on December 15, 1964.

42 See Proceedings 267 AIP Conference, 1992.

43 ESA Report SCI (91)7, August 1991.

44 The Italian X-ray astronomy satellite, Beppo-SAX, has been named in his honor.

45 The comet was classified as C/1997 H2 (SOHO).

46 Due to its duration exceeding the week assigned to each lead observer, this global corona observation was run in agreement with the previous or successive lead observer.

47 Workshop proceedings published in P. Maltby and B. Battrick (eds.), ESA SP – 1157, 1993. Some years later, in 1997, the Oslo meeting was followed by the ESLAB symposium on ‘Correlated Phenomena at the Sun, in the Heliosphere and in Geospace’ at ESTEC, *ESA SP*-415 (scientific coordinator B. Fleck), aimed at discussing the progress desirable in solar and heliospheric physics, solar variability, climate, and space weather.

48 Space instrumentation and missions studied for solar physics in the mid-1990s are briefly summarized in Antonucci and Simnett (1996).

49 The proceedings of the conference are published in *ESA SP*-417, 1998.

50 ‘Horizon 2000 Plus – European Space Science in the 21st Century’, *ESA SP*-1180, 1995 (B. Battrick, ed.).

51 A Solar Polar Orbiter was already present in the NASA fund requirement assessment formulated in 1985 (*EOS Transactions*, AGU, 66, 50, 1985).

52 In 1974 the IV European Symposium on Cosmic Rays took place in Frascati.

53 At that time ESA had not yet been established.

54 The ‘Solar Orbiter - High-Resolution Mission to the Sun and Inner Heliosphere’, by E. Marsch, E. Antonucci, P. Bochsler, J.-L. Bougeret, R. Harrison, R. Schwenn, and J.-C. Vial, was not the only proposal for a solar physics mission, since there was also interest in a ‘Next Generation SOHO’ mission.

55 In the Pre-Assessment Report, a LASCO-C2 type coronagraph was foreseen.

56 The results of the assessment study are found in the ‘Solar Orbiter - A High-Resolution Mission to the Sun and Inner Heliosphere’, Assessment Study Report. *ESA-SCI* (2000)6, July 2000 – Study Team members: E. Marsch, E. Antonucci, P. Bochsler, J.-L. Bougeret, R. Harrison, R. Schwenn, and J.-C. Vial, ESA Study Scientists: B. Fleck and R. Marsden.

57 The second Assessment Study results are published in: ‘Solar Orbiter Assessment Study Report – Exploring the Sun-heliosphere connection’, *ESA*/*SRE* (2009) 5, December 2009.

58 ‘Solar Orbiter – Exploring the Sun-heliosphere connection’, Definition Study Report. *ESA*/*SRE* (2011) 14.

59 The preliminary idea of ASPICS, a formation flyer solar coronagraph, was formulated in response to a CNES call for ideas back in 2004 (e.g., Vivès et al., 2005) with design studies also by part of Frassetto et al. (2005). The final proposal for developing the ASPIICS (the acronym changed) coronagraph on Proba-3 was submitted in 2009.

60 ‘Cosmic Vision – Space Science for Europe 2015 – 2025’, *ESA-BR*-247, October 2005.

61 Proceedings of the First Solar Orbiter Workshop, ESA SP-493. During this workshop the Solar Orbiter payload was scrutinized by four payload splinter groups, one of which was dedicated to coronagraphic instrumentation (Antonucci, 2001a,b).

62 HERSCHEL Principal Investigator: Daniel J. Moses. The acronym HERSCHEL was chosen to honor John Herschel, whose observation of Halley’s comet tail led him to hypothesize the presence of a ‘repulsive force’ acting on it. The proposal was submitted to NASA in September 2002 and was awarded in March 2003.

63 SCORE Principal Investigator: Ester Antonucci.

64 Initially AIA (Atmospheric Imaging Assembly) was the term used for the baseline set of EUV coronal imagers in the SDO announcement of opportunity, it was then also adopted for the assembly of EUV instruments built by LMSAL, flown on SDO in place of the EUV set of instruments designed in Belgium during phase A, named MAGRITTE (Rochus et al., 2004). SPECTRE complemented the diagnostic capability of MAGRITTE.

65 Principal Investigator: Ester Antonucci, Deputy Principal Investigator: Silvano Fineschi.

66 Principal proposer: E. Antonucci, coproposers: M. Romoli, G. Naletto, D. Spadaro, S. Fineschi, S. Cesare, P. Lamy, and the CIAO scientific and instrument Team.

67 Proposers: E. Antonucci, G. Naletto, S. Fineschi, M. Romoli, D. Spadaro, and the Co-I team with the international contributors: D. Moses, P. Lamy, and S. Solanki.

68 ‘Metis inverted occultation coronagraph for Solar Orbiter’, *OATo Technical Report*, 10 June 2009, by the Metis Optical Working Group: Silvano Fineschi, Vania DaDeppo, J. Dan Moses, Giampiero Naletto, Marco Romoli, Luca Poletto, and Luca Zangrilli.

69 A colleague of the Politecnico di Torino suggested that I submit a technological proposal to the Piedmont Regional Government; it was selected and financed in 2005. The idea was to develop a facility to integrate and calibrate solar instrumentation (UV and VL), and Silvano Fineschi was responsible for the facility design and implementation.

70 The contribution to the Science Requirement Document during the prestudy phase of the Kuafu project is reported in ‘A white light and HI Ly alpha Coronagraph for the Kuafu Mission’, by Ester Antonucci, Marco Romoli, Fabio Frassetto, Giampiero Naletto, and Daniel Telloni, based on a first optical design by Frassetto and Naletto.

71 In July 2006 the KuaFu science committee meeting and the International Living with a Star (ILWS) meeting were held at the time of the 36th COSPAR Scientific Assembly in Beijing. The second and third international symposia on the KuaFu project were held in Sanya in January 2007 and in Kunming in September 2008, respectively. Finally, in November 2010, I attended the International KuaFu Project Workshop in Beijing.

72 Astronomo Ordinario

73 The Archivio di Stato (State Archive) di Torino is home to almost one thousand years of historical documents.

74 The book was also published in an English translation and will soon be available on the Observatory’s web site.

75 Home of the Piedmont Regional Council. Both Palazzo Bricherasio and Palazzo Lascaris were built in the seventeenth century.

76 The exhibition *Akhenaton, il Faraone del Sole*, was held from February 27 to June 14, 2009.

77 The main local organizer was my colleague Alba Zanini of the National Institute of Nuclear Physics, INFN.

78 Galileo Galilei was rehabilitated by Pope John Paul II in 1992.

79 See: https://www.minorplanetcenter.net/db_search/show_object?object_id=22744.
